# Mitogen-activated protein kinase pathway and four genes involved in the development of benign prostatic hyperplasia: *in vivo* and *vitro* validation

**DOI:** 10.3389/fimmu.2025.1606607

**Published:** 2025-11-11

**Authors:** Jia-Min Gu, Xing-Pei Guo, Lan Wu, Lu-Yao Li, Cong Zhu, Tong Deng, Shuang-Ying Wang, Cheng Fang, Xian-Tao Zeng

**Affiliations:** 1 Department of Urology, Institute of Urology, Zhongnan Hospital of Wuhan University, Wuhan, Hubei, China; 2 Center for Evidence-Based and Translational Medicine, Zhongnan Hospital of Wuhan University, Wuhan, China; 3 Institutes of Evidence-Based Medicine and Knowledge Translation, Henan University, Kaifeng, Henan, China

**Keywords:** benign prostatic hyperplasia, proteomics, MAPK pathway, genetic markers, immunology

## Abstract

**Background:**

Benign prostatic hyperplasia (BPH) is a common age-associated disorder characterized by non-malignant proliferation of prostate tissues. However, its underlying molecular mechanisms remain incompletely elucidated.

**Methods:**

A rat model of BPH was established via surgical castration followed by testosterone propionate administration. Proteomic profiling was performed on prostate tissues from BPH and sham-operated rats. Public datasets of BPH patients and healthy individuals were also analyzed to validate the translational relevance. In addition, qPCR was conducted on both rat tissues and human-derived prostate cell lines to confirm gene expression levels. Furthermore, immunohistochemistry was performed on prostate tissue samples from BPH patients to examine the expression and localization of the target proteins.

**Results:**

Proteomic analysis revealed significant upregulation of four proteins—QPCT, ARHGEF37, FLNC, and LGALS7—in BPH model rats compared with controls. KEGG pathway enrichment identified the MAPK signaling pathway as a potential key regulator of BPH progression. Similar expression patterns were observed in human datasets. qPCR results further validated the elevated expression of these four genes in both rat and human BPH-related samples. Immunohistochemistry further demonstrated that all four proteins were highly expressed in prostate tissues from BPH patients.

**Conclusions:**

The aberrant overexpression of QPCT, ARHGEF37, FLNC, and LGALS7, along with dysregulation of the MAPK signaling pathway, may contribute to BPH pathogenesis. These findings provide new insights into the molecular mechanisms of BPH and may inform the development of diagnostic markers or therapeutic targets.

## 1 Introduction

Benign prostatic hyperplasia (BPH) is a highly prevalent, non-malignant condition affecting aging men, characterized by the hyperproliferation of epithelial and stromal cells within the prostate (1). It is frequently associated with lower urinary tract symptoms (LUTS), including urinary frequency, nocturia, and incomplete bladder emptying, significantly impairing quality of life and imposing a substantial healthcare burden globally (2, 3). Although hormonal dysregulation (4), particularly androgen signaling (5), has long been recognized as a central driver of BPH, the precise molecular mechanisms remain incompletely understood (6).

Accumulating evidence suggests that chronic inflammation and dysregulated immune responses contribute to the pathogenesis and progression of BPH (7). Inflammatory infiltrates and elevated levels of cytokines such as IL-6 and TNF-α have been detected in hyperplastic prostate tissues (8–10), implying a pivotal role for immune-mediated processes. However, how these immune events are regulated at the protein and signaling pathway level is yet to be fully elucidated.

The mitogen-activated protein kinase (MAPK) signaling pathway plays a critical role in regulating cell proliferation, apoptosis, and inflammation (11). Dysregulation of MAPK signaling, particularly the ERK, JNK, and p38 sub-pathways, has been implicated in various proliferative and inflammatory disorders. Recent studies have also demonstrated that the MAPK pathway plays a critical role in the development and progression of BPH (12, 13), where inflammation induced by aberrant MAPK activation may constitute a key pathological mechanism. However, few studies have explored its involvement in BPH using proteomics-based approaches or validated findings in both animal and human models.

In this study, we established a rat model of BPH via castration combined with testosterone propionate injection and performed comparative proteomic analysis of prostate tissues from BPH and sham-operated rats. This is a well-established and widely accepted method for inducing BPH in rodents, which has also been employed in our previous studies (14, 15). We identified four significantly upregulated proteins—QPCT, ARHGEF37, FLNC, and LGALS7—that may contribute to BPH development. KEGG enrichment analysis suggested the MAPK signaling pathway as a central mechanism. Importantly, we validated these findings in public human datasets and further confirmed differential gene expression through qPCR analysis in both rat tissues and human-derived prostate cell lines. In addition, immunohistochemical analysis of prostate tissues from BPH patients revealed that all four proteins were abundantly expressed in hyperplastic prostate tissues. These findings offer new insights into the immune-related molecular pathogenesis of BPH and provide potential targets for diagnostic and therapeutic intervention.

## 2 Materials and methods

### 2.1 Animals and samples

Male Sprague–Dawley (SD) rats weighing 300–350 g were obtained from the Animal Experiment Center of Zhongnan Hospital of Wuhan University. Rats were maintained under controlled environmental conditions (humidity 55 ± 10%, temperature 22 ± 2°C) on a 12-h light/dark cycle with free access to food and water. All experimental procedures were conducted in accordance with the Guide for the Care and Use of Laboratory Animals in China and the ethical principles of the Declaration of Helsinki. The study protocol was approved by the Institutional Animal Care and Use Committee (IACUC) of Wuhan University (approval number: 2018119). Animal handling and experimental design followed the 3Rs principles (Replacement, Reduction, and Refinement) to minimize animal use and suffering. All animals were allowed to acclimate under standard laboratory conditions for 1 week prior to experimentation. Rats were then randomly assigned into experimental groups. Surgical procedures were performed under deep anesthesia using intraperitoneal injection of sodium pentobarbital (40 mg/kg). Bilateral orchiectomy was performed in the BPH group, whereas sham-operated rats underwent identical procedures without removal of the testes. Postoperative care included close monitoring and supportive measures to reduce pain and distress. One week after surgery, rats in the BPH group received daily subcutaneous injections of testosterone propionate (5 mg/kg) for four consecutive weeks. Rats in the sham group received equal volumes of physiological saline on the same schedule. Five weeks post-surgery, all animals were humanely sacrificed, and ventral prostate tissues were carefully collected, snap-frozen in liquid nitrogen, and stored at −80°C for subsequent proteomic analysis. The experimental timeline is shown in **Supplementary Table 1**.

### 2.2 Cell culture and treatment

The human benign prostatic hyperplasia epithelial cell line BPH-1 (Cat. #BNCC339850) was kindly provided by the Department of Urology, Institute of Urology, Zhongnan Hospital of Wuhan University. The human prostate stromal cell line WPMY-1 (Cat. #GNHu36) was obtained from the Stem Cell Bank of the Chinese Academy of Sciences (Shanghai, China). BPH-1 cells were maintained in RPMI-1640 medium (Gibco, C11875500BT) supplemented with 10% fetal bovine serum (FBS; Gibco, 10270-106) and 1% penicillin G sodium/streptomycin sulfate. WPMY-1 cells were cultured in Dulbecco’s Modified Eagle Medium (DMEM; HyClone, SH30022.01) containing 5% FBS and 1% penicillin G sodium/streptomycin sulfate. All cell lines were recently authenticated and cultured under standard conditions in a humidified incubator at 37°C with 5% CO_2_ and 95% air.

### 2.3 RNA extraction, complementary DNA synthesis, and quantitative real-time polymerase chain reaction

Total RNA was extracted from cells and tissues using an RNA extraction kit (Promega, USA) according to the manufacturer’s instructions. RNA concentration and purity were measured using a NanoDrop 2000 spectrophotometer (Thermo Scientific, USA). Complementary DNA (cDNA) was synthesized from total RNA using the PrimeScript™ RT Reagent Kit (Takara, RR047A, Japan). Quantitative real-time PCR (qPCR) was performed using the SYBR^®^ Premix Ex Taq™ II kit (Takara, Japan) on the CFX Connect™ Real-Time PCR Detection System (Bio-Rad, USA). Each reaction was carried out in triplicate. The amplification protocol and primer sequences are provided in **Supplementary Table 2**. Gene expression levels were calculated using the 2^–ΔΔCt method, with β-actin used as the internal control. Primers were designed using the NCBI Primer Design Tool and synthesized by a commercial supplier.

### 2.4 Protein extraction, digestion, and fractionation

Prostate tissue samples were cut into small pieces, lysed in buffer containing 2 mM EDTA, and treated with dithiothreitol (DTT) and iodoacetamide (IAM) for protein reduction and alkylation. After centrifugation, protein concentrations were determined by the Bradford assay, and integrity was assessed by SDS-PAGE. Proteins were digested with sequencing-grade trypsin, desalted, and vacuum-dried. Peptides (200 µg) were fractionated using high-pH reversed-phase high-performance liquid chromatography (HPLC) on a C18 column, and fractions were lyophilized for downstream mass spectrometry analysis.

### 2.5 Mass spectrometry acquisition and data analysis

Peptide samples were separated using nanoLC and analyzed on a Q Exactive HF mass spectrometer in both data-dependent acquisition (DDA) and data-independent acquisition (DIA) modes. DDA data were processed with MaxQuant against the UniProtKB/Swiss-Prot human database to build a spectral library, and DIA data were analyzed with Spectronaut using iRT calibration and mProphet scoring. Protein and peptide identifications were filtered at a false discovery rate (FDR) ≤1%. Differentially expressed proteins (DEPs) were determined using MSstats with thresholds of fold change ≥2.0 and p < 0.05. Data have been deposited in the ProteomeXchange Consortium (PXD032395).

### 2.6 Dataset selection and KEGG pathway analysis of human prostate tissues

Gene expression profiles of BPH and normal prostate tissues were obtained from the GEO database (accession number: GSE119195, n = 8). Differentially expressed genes (DEGs) were identified using the limma package in R (version 3.7.1). DEGs were defined based on the following criteria: adjusted p-value (Benjamini–Hochberg FDR) < 0.05 and |log_2_ fold change (log_2_FC)| ≥ 1. To explore the underlying biological functions and pathways, KEGG pathway enrichment analysis was conducted using the clusterProfiler package. This enabled comprehensive functional annotation of DEGs and their involvement in key molecular pathways.

### 2.7 Tissue immunohistochemical staining

Formalin-fixed, paraffin-embedded prostate tissues from BPH patients were sectioned at a thickness of 2–5 μm using a microtome (Leica RM2016, Leica Microsystems, Wetzlar, Germany), mounted onto glass slides, and baked at 60°C for 1 h. Sections were deparaffinized in xylene, rehydrated through graded ethanol, and subjected to antigen retrieval in citrate buffer (pH 6.0) using microwave heating (medium power for 8 min until boiling, 8 min hold, followed by medium-low power for 7 min). After cooling, endogenous peroxidase activity was blocked with 3% hydrogen peroxide for 25 min at room temperature in the dark. Non-specific binding was blocked with 3% bovine serum albumin (BSA) for 30 min at room temperature, followed by overnight incubation at 4°C with the primary antibodies against QPCT, FLNC, ARHGEF37, and LGALS7 (listed in **Supplementary Table 3**). After washing in PBS, sections were incubated with secondary antibodies (listed in **Supplementary Table 3**; 1:2,000, Abcam) for 50 min at room temperature. Immunoreactivity was visualized using 3,3′-diaminobenzidine (DAB) chromogen solution, and nuclei were counterstained with hematoxylin. Slides were dehydrated through graded ethanol, cleared in xylene, and mounted with neutral resin. Positive staining appeared as brown coloration in the cytoplasm or nucleus, with hematoxylin counterstaining nuclei blue.

### 2.8 Statistical analysis

All statistical analyses were performed using SPSS software version 22.0 (IBM Corp., USA). All experiments were conducted in triplicate unless otherwise stated. Data are presented as mean ± standard deviation (SD), except where the standard error of the mean (SEM) is explicitly indicated. The chi-square test and Student’s t-test were employed to evaluate statistical differences between groups. All tests were two-tailed, and a p-value < 0.05 was considered statistically significant.

## 3 Results

### 3.1 Identification of differentially expressed proteins in BPH and sham prostate tissues

**Figure 1:**
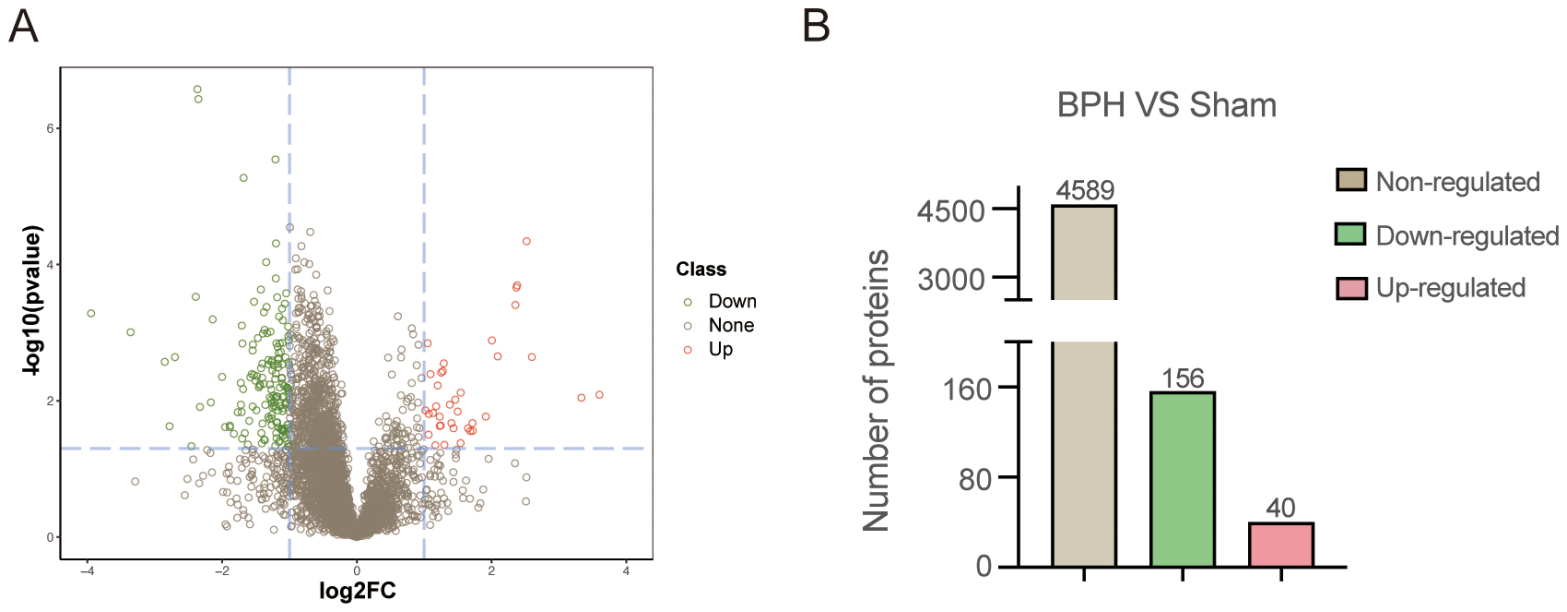
Proteomic profiling of prostate tissues in BPH and sham-operated rats. **(A)** Volcano plot illustrating differentially expressed proteins (DEPs) between the BPH and sham groups. The X-axis represents log_2_ fold change, and the Y-axis represents –log_10_ (p-value). Red dots indicate significantly upregulated proteins, green dots indicate significantly downregulated proteins, and gray dots represent non-significantly changed proteins. **(B)** Quantification of identified proteins in BPH and sham groups. Among all DEPs, 40 were upregulated, 156 were downregulated, and 4,589 showed no significant regulation.

To identify differentially expressed proteins (DEPs) associated with BPH, conventional data-dependent acquisition (DDA) mass spectrometry was first employed to construct a spectral library using prostate tissue samples obtained from seven rats (three BPH and four sham-operated controls). Subsequently, data-independent acquisition (DIA) mass spectrometry was applied for quantitative proteomic analysis. Protein abundance data were processed and statistically analyzed using the MSstats package. A total of 4,785 proteins were identified. Proteins exhibiting a fold change ≥2.0 and a P-value <0.05 were defined as significantly differentially expressed. Based on these criteria, 196 DEPs were identified in the BPH group compared with the sham group (**Figure 1**). Among these, 40 proteins were significantly upregulated, whereas 156 proteins were significantly downregulated. Detailed UniProt annotations for the upregulated and downregulated proteins are provided in **Table 1** and **Supplementary Table 4**, respectively.

**Table 1:** Quantitative differential results of up-regulated proteins and protein-coding genes for comparison group. .

Protein	Description	P value	Gene symbol	log2FC
P97590	Galectin-7	0.00005	Lgals7	2.52071
P24464	Cytochrome P450 4A12	0.00017	Cyp4a12	4.58585
A1IGU3	Rho guanine nucleotide exchange factor 37	0.00020	Arhgef37	2.37975
D3ZW27	Mitogen-activated protein kinase kinasekinase 5	0.00022	Map3k5	2.36828
D4A2K1	4-hydroxy-2-oxoglutarate aldolase 1	0.00039	Hoga1	2.35328
Q6AYC4	Macrophage-capping protein	0.00130	Capg	2.00481
D3ZHA0	Filamin-C	0.00143	Flnc	1.05136
D4A9Q5	Carboxypeptidase M	0.00223	Cpm	2.09145
D4AC85	Glutaminyl-peptide cyclotransferase	0.00227	Qpct	2.59760
D3ZCV0	Actinin alpha 2	0.00282	Actn2	1.29027
Q6IFV1	Keratin, type I cytoskeletal 14	0.00367	Krt14	1.27638
F1LRH4	Laminin subunit gamma 2	0.00385	Lamc2	1.25180
Q62662	Tyrosine-protein kinase FRK	0.00406	Frk	1.09383
D3ZCR4	Protein phosphatase 4, regulatory subunit 3B	0.00502	Ppp4r3b	5.74577
D3ZZE3	Armadillo-like helical domain-containing 3	0.00597	Armh3	1.20040
D3ZCG2	Kinesin family member 21A	0.00759	Kif21a	1.54148
Q9R1T5	Aspartoacylase	0.00816	Aspa	3.60032
A0A1W2Q6H4	Cytochrome P450, family 4, subfamily f, polypeptide 17 (Fragment)	0.00902	Cyp4f17	3.33378
Q9Z122	Acyl-CoA 6-desaturase	0.00963	Fads2	1.46187
O70597	Peroxisomal membrane protein 11A	0.01141	Pex11a	1.38021
A0A0G2JY11	Tetraspanin	0.01203	Tspan9	1.17692
P70490	Lactadherin	0.01383	Mfge8	1.01702
P23562	Band 3 anion transport protein	0.01437	Slc4a1	1.49700
Q5XID7	Armadillo repeat-containing X-linked protein 3	0.01511	Armcx3	1.13071
P15205	Microtubule-associated protein 1B	0.01556	Map1b	1.06760
A0A0G2JWD0	Prominin 1	0.01706	Prom1	1.91537
D3ZPN5	Mitochondrial poly(A) polymerase	0.01708	Mtpap	1.22954
P32232	Cystathionine beta-synthase	0.02127	Cbs	1.71582
Q8CJ11	Adhesion G-protein coupled receptor G2	0.02128	Adgrg2	1.40415
Q5BKC6	HSPB1-associated protein 1	0.02306	Hspbap1	1.24457
P31214	3-oxo-5-alpha-steroid 4-dehydrogenase 2	0.02343	Srd5a2	1.23084
G3V8C0	Dynactin subunit 5	0.02531	Dctn5	1.43532
O08701	Arginase-2, mitochondrial	0.02539	Arg2	1.65066
M0R9N6	2 -5 oligoadenylate synthetase 1K	0.02763	Oas1k	1.72000
Q63279	Keratin, type I cytoskeletal 19	0.02773	Krt19	1.68246
Q4TU93	C-type mannose receptor 2	0.03139	Mrc2	1.06486
A0A096MKF1	Inositol hexakisphosphate and diphosphoinositol-pentakisphosphate kinase (Fragment)	0.04073	Ppip5k2	5.62064
A0A0G2JU12	Microsomal glutathione S-transferase 2	0.04187	Mgst2	1.54048
P07150	Annexin A1	0.04437	Anxa1	1.29916
D3ZBN3	Eph receptor A2	0.04501	Epha2	1.16481

### 3.2 GO enrichment analysis

**Figure 2:**
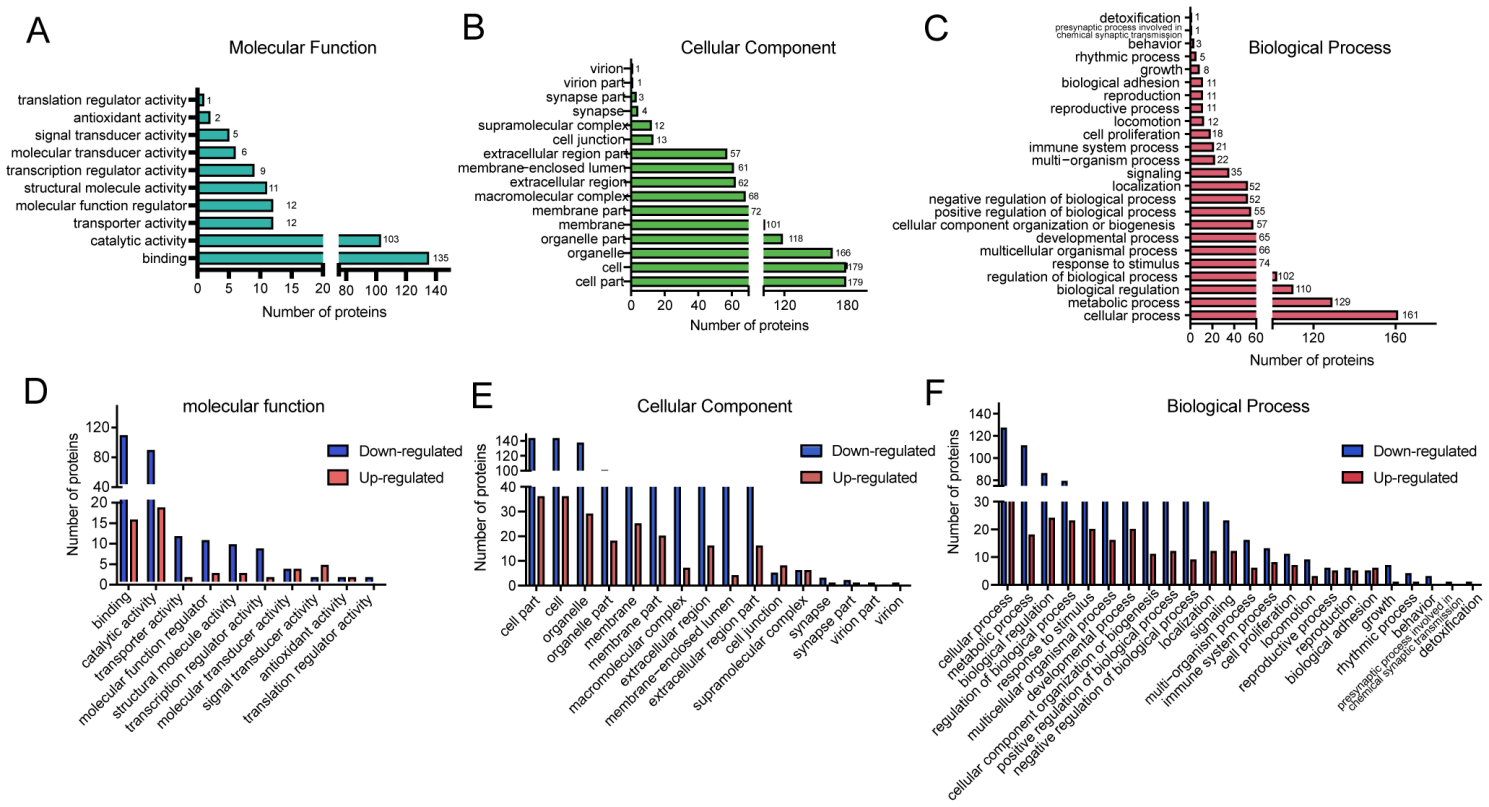
Gene Ontology (GO) classification of DEPs in BPH and sham rats. **(A–C)** GO functional classification of DEPs according to three major categories: molecular function (MF), cellular component (CC), and biological process (BP). The X-axis indicates the number of DEPs, and the Y-axis denotes the corresponding GO terms. **(D–F)** GO classification of upregulated and downregulated DEPs. The X-axis indicates GO annotation terms, and the Y-axis represents the number of DEPs in each category.

To investigate the functional significance of DEPs, Gene Ontology (GO) annotation was conducted using Blast2GO software. The corresponding protein information and enrichment visualization results are presented in **Figure 2** and **Supplementary Table 5**. In terms of molecular function (**Figure 2A**), the most significantly enriched GO categories were binding and catalytic activity. For cellular components (**Figure 2B**), DEPs were predominantly associated with cell, cell part, and organelle. The most enriched biological process terms included cellular process, metabolic process, biological regulation, regulation of biological process, and response to stimulus (**Figure 2C**). To further illustrate the distribution of DEPs, GO functional classification maps were generated to distinguish upregulated and downregulated proteins (**Figures 2D–F**). Overall, both upregulated and downregulated proteins were involved in similar structural and functional pathways. However, distinct enrichment patterns were observed between the two groups for certain GO terms within the biological process, cellular component, and molecular function categories, suggesting differential biological roles.

### 3.3 KOG classification

**Figure 3:**
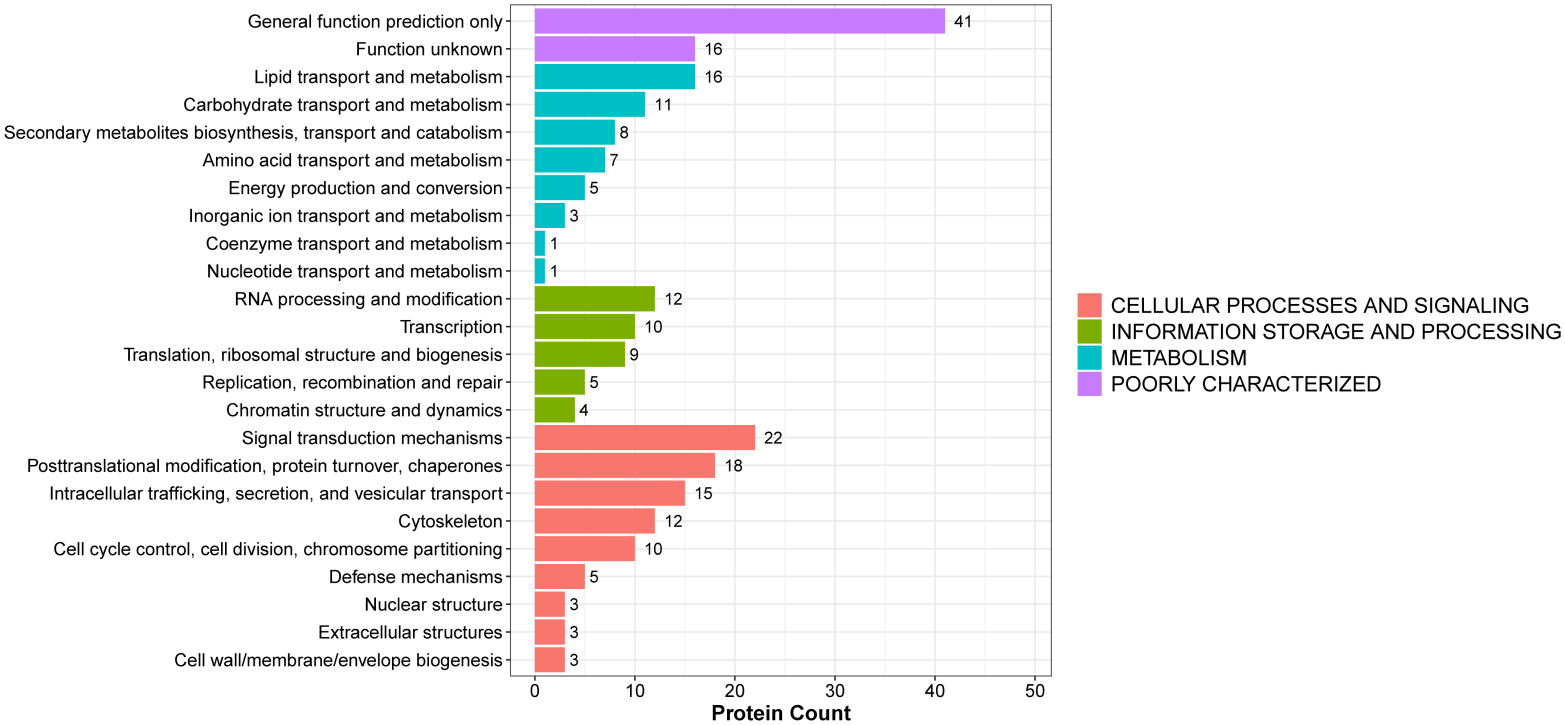
KOG functional annotation of DEPs in BPH and sham rats. The histogram displays the classification of DEPs according to the eukaryotic orthologous group (KOG) categories: cellular processes and signaling, information storage and processing, metabolism, and poorly characterized functions. The Y-axis shows the KOG categories, and the X-axis indicates the number of proteins per category. Different colors represent different functional groups.

To further explore the potential functions of the identified DEPs, KOG (Eukaryotic Orthologous Groups) classification analysis was performed (**Figure 3** and **Supplementary Table 6**). The DEPs were categorized based on functional orthology using the KOG database. The most prominently enriched KOG category was cellular processes and signaling, with a significant proportion of DEPs involved in posttranslational modification, protein turnover, chaperone activity, intracellular trafficking, secretion, and vesicular transport. In addition, a considerable number of DEPs were classified under information storage and processing, metabolism, and poorly characterized functional groups. These findings suggest that the biological alterations associated with BPH may be closely related to disruptions in protein processing, intracellular communication, and fundamental metabolic activities.

### 3.4 KEGG pathway analysis

**Figure 4:**
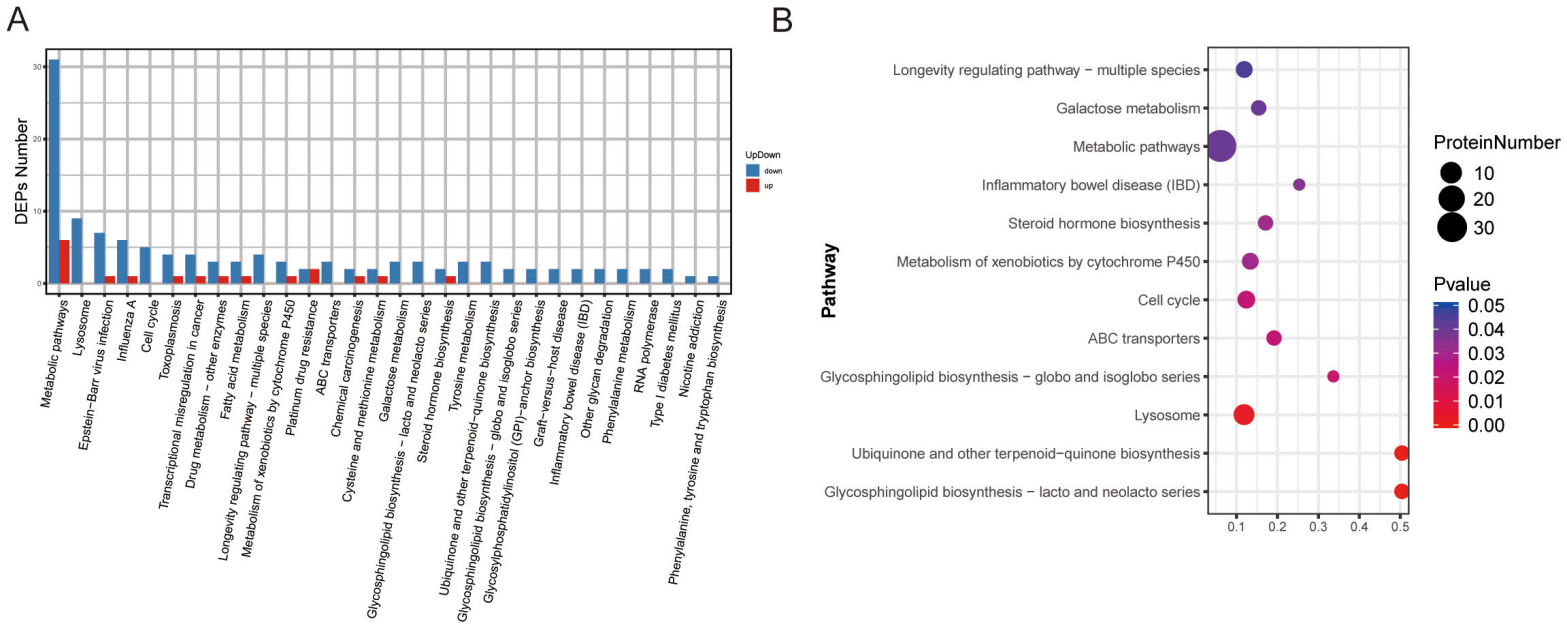
KEGG pathway classification and enrichment analysis of DEPs in BPH and sham rats. **(A)** KEGG pathway classification of DEPs. The X-axis represents KEGG pathway terms, and the Y-axis indicates the number of upregulated or downregulated proteins assigned to each pathway. **(B)** KEGG pathway enrichment analysis. The X-axis shows the enrichment factor (number of DEPs in a pathway divided by total proteins identified in the same pathway), whereas the Y-axis denotes the pathway terms. Dot size indicates the number of DEPs, and color intensity reflects statistical significance.

To further elucidate the biological roles of the identified DEPs in the context of BPH, KEGG (Kyoto Encyclopedia of Genes and Genomes) pathway enrichment analysis was performed (**Supplementary Table 7**). A total of 30 significantly enriched pathways were identified among all DEPs. The most prominently enriched pathways included metabolic pathways and lysosome-related pathways. Specifically, the upregulated DEPs were annotated to 12 major pathways, with metabolic pathways and platinum drug resistance emerging as the most significantly enriched categories (**Figure 4A**). The top 12 enriched pathways based on statistical significance are presented in **Figure 4B**. Within the metabolic pathway category, several age-associated proteins were notably enriched, including Arginase-2 (O08701), lysosomal thioesterase PPT2 (O70489), carboxylesterase 1D (P16303), and lysosomal acid phosphatase (P20611). These proteins have previously been implicated in age-related metabolic remodeling and lysosomal activity, both of which are known to contribute to prostate tissue hypertrophy and dysfunction in BPH (**Supplementary Table 7**).

These results suggest that altered metabolic and lysosomal pathways may play a critical role in the molecular pathogenesis of BPH and represent potential targets for therapeutic intervention.

### 3.5 Subcellular localization and protein–protein interaction network analyses

**Figure 5:**
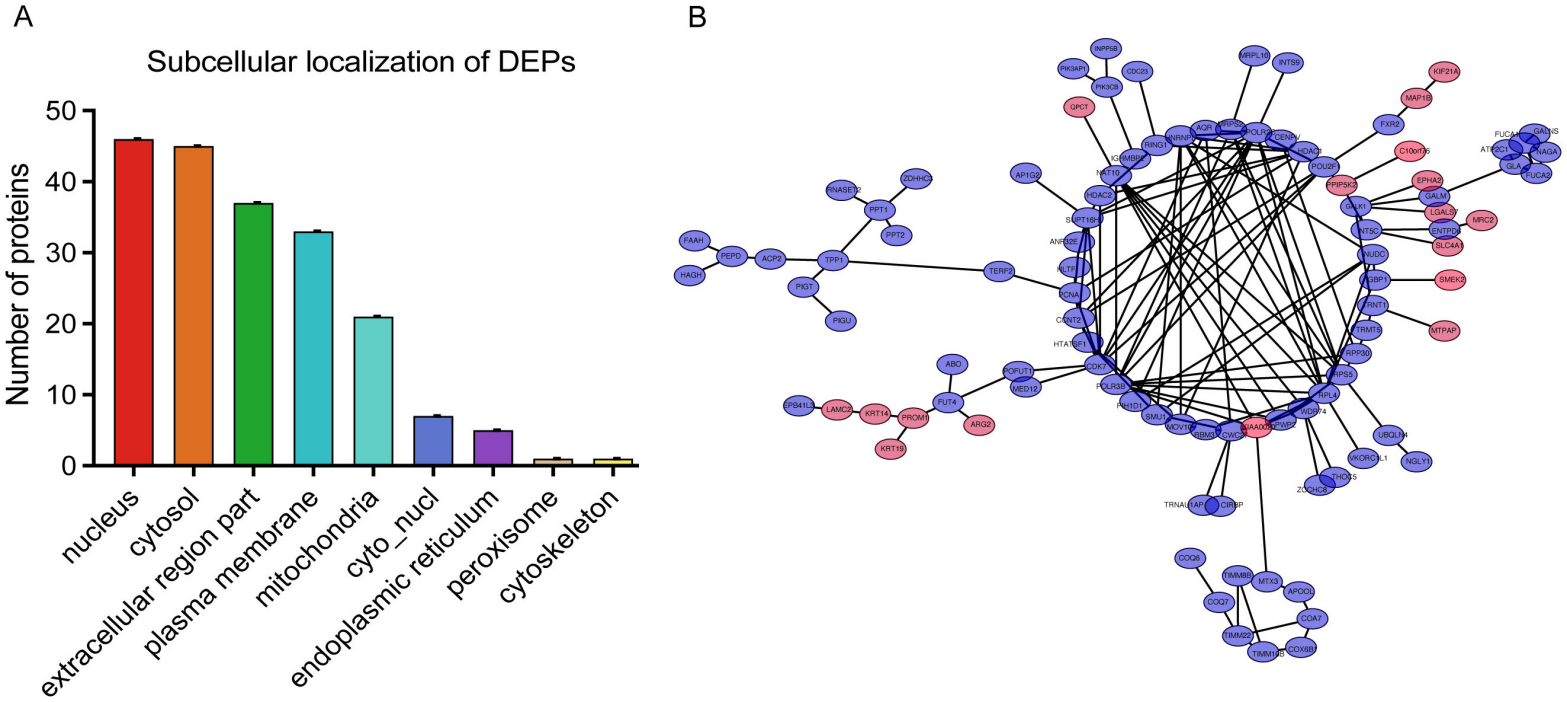
Subcellular localization and protein–protein interaction (PPI) network of DEPs. **(A)** Subcellular localization analysis of DEPs. The X-axis shows subcellular compartments, and the Y-axis indicates the number of proteins localized to each compartment. **(B)** PPI network visualization of DEPs. Pink and purple nodes represent upregulated and downregulated proteins, respectively.

To gain insights into the cellular context of the DEPs identified in BPH, subcellular localization prediction was performed using the WoLF PSORT algorithm (**Figure 5A**). The results revealed that the DEPs were predominantly localized in the nucleus, cytosol, extracellular space, and plasma membrane, suggesting that BPH-related protein alterations are involved in a wide range of cellular compartments and may influence both intracellular signaling and extracellular interactions (**Supplementary Table 8**). To further explore the potential functional relationships among the DEPs, a protein–protein interaction (PPI) network was constructed using the STRING database (version 11.5), applying a high-confidence interaction score threshold (≥0.700) and excluding disconnected nodes to improve network clarity (**Figure 5B**). The resulting interaction network was visualized and analyzed using Cytoscape software (version 3.9.0). Network topology analysis identified Rps5, a key ribosomal protein involved in translation, ribosomal structure, and biogenesis, as the top hub protein based on node degree (**Supplementary Table 9**). This finding suggests that alterations in protein synthesis machinery may play a central role in the molecular pathogenesis of BPH, potentially contributing to aberrant cell proliferation and hypertrophy observed in prostatic tissues.

### 3.6 Microarray analysis of mRNA expression in human BPH and normal prostate tissues

**Figure 6:**
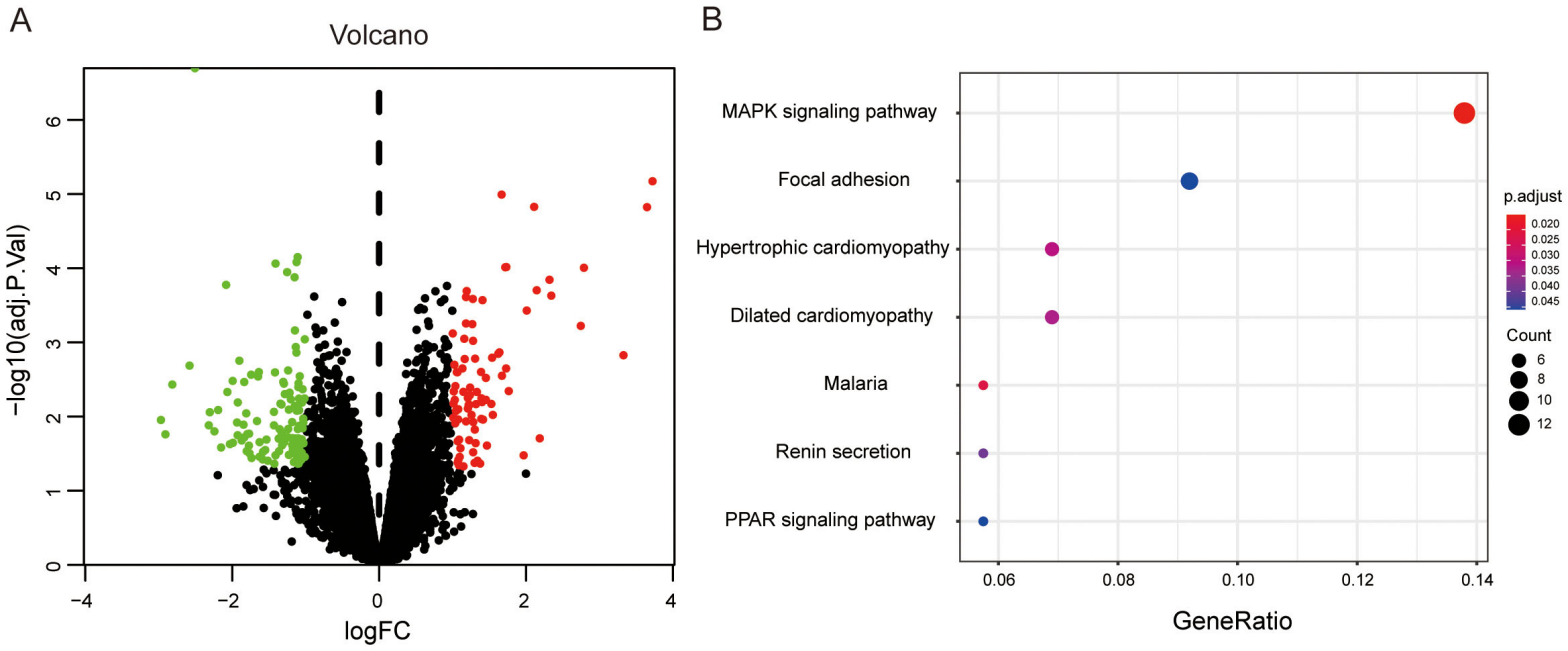
Differential gene expression analysis in human BPH versus normal prostate tissues. **(A)** Volcano plot of DEGs between BPH and normal prostate tissues. Red dots denote upregulated genes, and green dots denote downregulated genes. **(B)** KEGG pathway enrichment analysis of 201 DEGs. The X-axis represents the ratio of DEGs in a given pathway relative to the total number of genes annotated in that pathway. The Y-axis shows the pathway terms. Dot size corresponds to the number of DEGs involved in each pathway.

To complement the proteomic findings and further elucidate the molecular mechanisms underlying BPH, we performed a bioinformatics analysis of transcriptomic data retrieved from the Gene Expression Omnibus (GEO) database (accession number: GSE119195). This dataset comprises mRNA expression profiles from three normal prostate tissues and five BPH samples. A total of 201 differentially expressed genes (DEGs) were identified through microarray analysis (**Figure 6**). Functional enrichment indicated that these DEGs are predominantly involved in the MAPK signaling pathway, which is well known for its regulatory roles in cell proliferation, differentiation, and apoptosis in prostate tissue. This aligns with the established view that aberrant activation of MAPK signaling contributes to prostatic epithelial and stromal hyperplasia. In addition, a significant subset of DEGs was enriched in the PPAR-γ signaling pathway, which has been increasingly recognized as a critical molecular link between systemic metabolic disorders and BPH pathogenesis. Notably, this observation is consistent with our proteomic KEGG analysis, which also highlighted dysregulation in metabolic pathways as a key feature of BPH. Taken together, these transcriptomic findings provide further evidence that the MAPK and metabolic signaling pathways play central roles in the development of BPH at both the mRNA and protein levels. This cross-platform consistency strengthens the validity of the proposed molecular mechanisms and offers potential targets for future therapeutic intervention.

### 3.7 Validation of DEG expression in human cell lines and rat models

**Figure 7:**
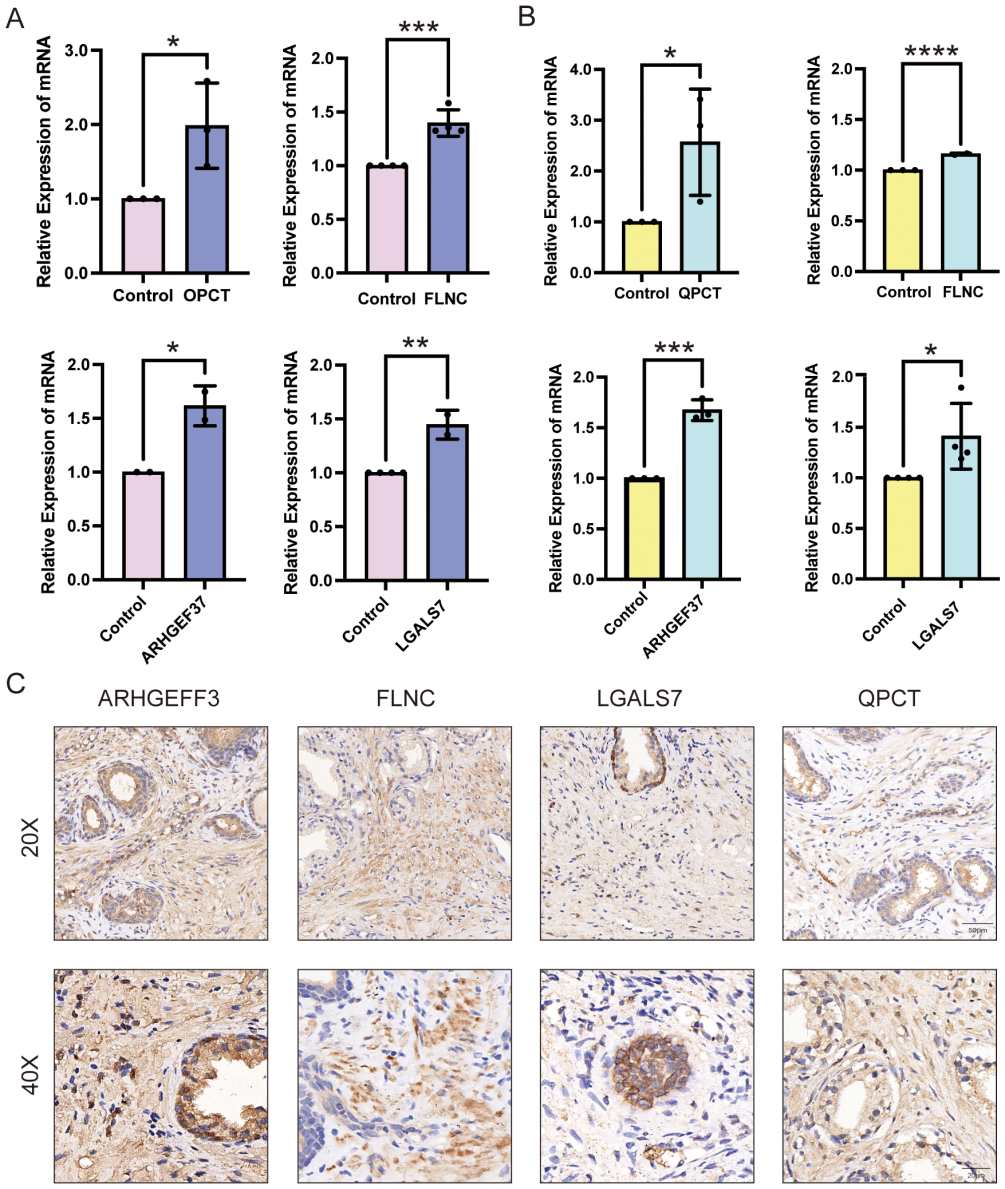
Expression validation of QPCT, FLNC, ARHGEF37, and LGALS7 in BPH samples. **(A)** Relative mRNA expression levels of QPCT, FLNC, ARHGEF37, and LGALS7 in BPH-1 epithelial cells compared with WPMY-1 stromal cells. **(B)** Relative mRNA expression levels of the same genes in prostate tissues from BPH versus sham-operated rats. **(C)** Immunohistochemical staining of ARHGEF37, FLNC, LGALS7, and QPCT for BPH human prostate. Scale bar = 50 μm and 20μm. Data were analyzed using unpaired two-tailed t-tests. P < 0.05 (*), P < 0.01 (**), P < 0.001 (***), P < 0.0001 (****).

To validate the expression patterns of key upregulated proteins identified in BPH, qPCR was employed to assess mRNA levels both *in vivo* and *in vitro*. Specifically, we examined the expression of four candidate genes—FLNC, QPCT, ARHGEF37, and LGALS7—in a rat model of BPH as well as in human prostate cell lines. The results demonstrated that all four genes were significantly upregulated in the BPH rat prostate tissues compared with sham-operated controls. Similarly, elevated expression levels were observed in the BPH-1 human prostatic epithelial cell line relative to the normal WPMY-1 stromal cell line (**Figures 7A, B**). These findings provide consistent molecular evidence across species and models, further supporting the involvement of these genes in the pathogenesis of BPH. Together, these data corroborate the proteomic findings and reinforce the notion that FLNC, QPCT, ARHGEF37, and LGALS7 may serve as potential molecular markers or functional mediators of BPH.

### 3.8 Immunohistochemical analysis of BPH patient tissues

Immunohistochemistry was performed on prostate tissues obtained from three BPH patients to examine the protein expression patterns of ARHGEF37, FLNC, LGALS7, and QPCT. As shown in **Figure 7C**, all four proteins exhibited strong positive staining in hyperplastic prostate tissues, with immunoreactivity detected in both glandular epithelial cells and stromal cells. Both low- (20×) and high- (40×) magnification images consistently revealed intense brown DAB staining, indicating high expression levels, whereas nuclei were counterstained blue with hematoxylin. Similar staining patterns were observed across all three clinical samples, confirming that these proteins are abundantly expressed in BPH patient tissues. Whole-slide scanned images of all three specimens are provided in the **Supplementary Figure 1**.

## 4 Discussion

Chronic inflammation has been widely recognized as a major contributor to the pathogenesis of BPH. Infiltration of immune cells, secretion of inflammatory cytokines, and stromal remodeling forms a permissive microenvironment for sustained prostatic overgrowth (16, 17). Among the various signaling pathways implicated, the MAPK signaling pathway acts as a central mediator, integrating extracellular inflammatory stimuli with intracellular responses such as proliferation, apoptosis, and fibrosis. Accumulating evidence has demonstrated that the MAPK signaling pathway may play a pivotal role in the progression of BPH (18, 19). In this study, four upregulated proteins—QPCT, ARHGEF37, FLNC, and LGALS7—were identified in the prostates of BPH model rats and qPCR experiments confirmed their upregulation in BPH-related samples including rat and human. These proteins are closely associated with immune regulation and may modulate MAPK signaling activity.

QPCT is considered attractive therapeutic targets in many human disorders, such as neurodegenerative diseases, and a range of inflammatory conditions, as well as for cancer immunotherapy, because of their capacity to modulate cancer immune checkpoint proteins (20, 21). Interestingly, emerging evidence suggests that senescent cells may suppress inflammatory responses via QPCT upregulation. Given that BPH is an age-associated condition, QPCT may also play a critical role in mediating the effects of cellular senescence on prostatic tissue homeostasis (22).

Rho family guanine nucleotide exchange factors (Rho-GEFs/ARHGEFs) are a family of proteins implicated in the activation of small GTPases by cycling from the inactive GDP-bound state to the active GTP-bound state (23). ARHGEF37, a member of the Rho guanine nucleotide exchange factor family, can increase the Cdc42-GTP level in HCC cells, and enhanced the extravasation and lung metastatic capability of HCC cells through promoting the formation of invadopodia, consequently resulting in disruption of the interaction between endothelial cells and pericytes (24) and also identified as key biomarkers for fibromyalgia, which is implicated in critical processes such as ion homeostasis, cell signaling, and neurobiological functions (25).

Vertebrates have three filamin proteins, FLNA, FLNB, and FLNC. Only mutations in FLNC result in muscle disorders, including muscular dystrophies, myofibrillar myopathy, distal myopathy, and cardiomyopathy (26). FLNC, traditionally considered a structural protein, plays an emerging role in mechanosensitive signaling and inflammatory cell adhesion. Recent studies have shown that FLNC interacts with Dishevelled-2 (Dvl2) to activate the Wnt/β-catenin signaling pathway and regulate skeletal muscle development, whereas FLNC deficiency induces autophagy or mitosis and contributes to muscle atrophy (26), processes which have recently been implicated as key factors in the progression of BPH and the development of LUTS (18, 27).

Galectins, a family of evolutionarily conserved glycan-binding proteins, play key roles in diverse biological processes including tissue repair, adipogenesis, immune cell homeostasis, angiogenesis, and pathogen recognition (28). It can directly activate MAPK and JNK pathways in epithelial cells under inflammatory conditions. Rita et al. reported that Galectin-7 is frequently overexpressed in various cancer types (29). While its precise function appears to be cancer-type dependent, Galectin-7 is broadly implicated in regulating cell adhesion, apoptosis, proliferation, and immune modulation (30, 31). Interestingly, Chen et al. demonstrated that LGALS7 exerts anti-inflammatory effects by suppressing the MAPK pathway through downregulation of miR-146a (32); however, although our study confirmed its upregulated expression in BPH samples, the functional significance and underlying mechanisms in the context of BPH remain to be elucidated.

Together, these proteins may form a functional inflammatory axis that integrates immune activation, mechanical stress, and MAPK pathway signaling, driving persistent prostatic hyperplasia. In the aging prostate, where immune surveillance is compromised, this axis may operate as a positive feedback loop: Inflammation activates MAPK signaling, which in turn amplifies immune and fibrotic responses. This may underlie the persistence and progression of BPH, particularly in inflammation-dominant subtypes.

Furthermore, it is worth exploring whether these proteins may represent potential therapeutic targets. QPCT has been identified as a potential therapeutic target due to its role in modulating immune cell–mediated phagocytosis of tumor cells, and considerable efforts have been made to develop small-molecule inhibitors against this enzyme (33, 34). Galectins play multifaceted roles in tissue fibrosis and cancer and have been identified as promising therapeutic targets (35) for the treatment of these pathologies (36). Although these small-molecule inhibitors have been validated in various disease models, they have not yet been applied to BPH, despite the potential of immune and inflammation-targeted therapies to inhibit its onset and progression. Therefore, our findings provide preliminary evidence and a valuable reference for future research in this direction.

Despite these important findings, several limitations should be acknowledged. First, both the proteomic analysis in rats and the transcriptomic data from human databases were based on relatively small sample sizes, which may limit the generalizability of the results. Second, the use of a rat model, while mechanistically valuable, cannot fully replicate the anatomical and hormonal complexity of the human prostate. Third, no *in vitro* functional validation was performed to confirm the causal roles of the identified proteins in MAPK activation or BPH progression. Nevertheless, these limitations also suggest future research directions.

In summary, this study provides novel insights into the inflammation–MAPK–proliferation axis in BPH. By identifying inflammation-related proteins that may modulate MAPK signaling, our findings offer potential molecular targets and deepen the understanding of BPH pathogenesis. Despite certain limitations, these results have significant translational relevance and lay a foundation for future mechanistic and therapeutic research.

## 5 Conclusion

This study identifies QPCT, ARHGEF37, FLNC, and LGALS7 as four key proteins that are abnormally upregulated in BPH and potentially involved in the activation of the MAPK signaling pathway. These molecules are associated with inflammatory regulation, cytoskeletal remodeling, and immune cell dynamics and may collectively contribute to the chronic inflammatory microenvironment that drives prostatic overgrowth. Our integrative analysis—combining a rat BPH model, proteomic profiling, human dataset validation, qPCR confirmation, and immunohistochemical analysis of clinical BPH specimens—provides new mechanistic insights into the pathogenesis of BPH. While further functional studies are necessary, our findings highlight novel candidate targets for diagnosis and therapy, particularly for patients with inflammation-dominant BPH phenotypes. Targeting the identified proteins or modulating the MAPK signaling axis may represent a promising direction for non-hormonal therapeutic intervention. Overall, this work lays a molecular foundation for future translational research into the immune and signaling pathways underlying BPH.

## Data Availability

The datasets presented in this study can be found in online repositories. The names of the repository/repositories and accession number(s) can be found in the article/**Supplementary Material**.
