# Supplementary material for: Mitogen-activated protein kinase pathway and four genes involved in the development of benign prostatic hyperplasia: in vivo and vitro validation
Source: Front Immunol. 2025 Nov 11;16:1606607. doi: 10.3389/fimmu.2025.1606607 (PMC12644057; doi:10.3389/fimmu.2025.1606607)
Supplement: Supplementary file 1 [file DataSheet1.docx]

**Supplementary Figure 1. Immunohistochemical staining of prostate tissues from three patients with benign prostatic hyperplasia (BPH).**

Representative serial sections of prostate tissues obtained from three BPH patients (Patient 1–3) were stained using immunohistochemistry (IHC) with antibodies against ARHGEF3, FLNC, Galectin-7, and QPCT, as indicated on the left. For each patient, consecutive tissue sections from the same specimen were used for staining with different antibodies to ensure consistency in histological context. Differential expression patterns of the indicated proteins are observed across patients and antibodies. All images are shown at the same magnification.
