# Supplementary material for: Mitogen-activated protein kinase pathway and four genes involved in the development of benign prostatic hyperplasia: in vivo and vitro validation
Source: Front Immunol. 2025 Nov 11;16:1606607. doi: 10.3389/fimmu.2025.1606607 (PMC12644057; doi:10.3389/fimmu.2025.1606607)
Supplement: Supplementary file 3 [file Table1.docx]

| **Time Point** | **Procedure** | **Details/Notes** |
| --- | --- | --- |
| Day -7 to 0 | **Acclimatization** | Rats were acclimated under standard laboratory conditions (22 ± 2°C, 55 ± 10% humidity, 12-h light/dark cycle) with free access to food and water for 1 week. |
| Day 0 | **Grouping + Surgery** | Under deep anesthesia (intraperitoneal sodium pentobarbital, 40 mg/kg), BPH group underwent bilateral orchiectomy; sham group underwent identical procedures without testis removal. Postoperative monitoring and analgesia were provided. |
| Days 1–7 | **Postoperative Recovery** | Close health monitoring and supportive care to minimize pain and distress. |
| Days 7–35 | **Drug Intervention (4 weeks)** | BPH group received daily subcutaneous injections of testosterone propionate (5 mg/kg); sham group received equal volumes of physiological saline. |
| Day 35 | **Sacrifice and Tissue Collection** | Five weeks post-surgery, rats were humanely sacrificed. Ventral prostate tissues were rapidly collected, snap-frozen in liquid nitrogen, and stored at −80°C for proteomic analysis. |

**Supplementary** **Table 1. Experimental timeline for the establishment of the BPH rat model**

Reference:

1. Li LY, Han J, Wu L, et al. Alterations of gut microbiota diversity, composition and metabonomics in testosterone-induced benign prostatic hyperplasia rats. Mil Med Res. 2022;9(1):12. doi:10.1186/s40779-022-00373-4.

2. Wang SY, Cai Y, Hu X, et al. P. gingivalis in oral-prostate axis exacerbates benign prostatic hyperplasia via IL-6/IL-6R pathway. Mil Med Res. 2024;20;11(1):30. doi: 10.1186/s40779-024-00533-8.

3. Zhang J, Zhang M, Tang J, et al. Animal models of benign prostatic hyperplasia. Prostate Cancer Prostatic Dis. 2021;24(1):49-57. doi: 10.1038/s41391-020-00277-1.
