# Supplementary material for: Mitogen-activated protein kinase pathway and four genes involved in the development of benign prostatic hyperplasia: in vivo and vitro validation
Source: Front Immunol. 2025 Nov 11;16:1606607. doi: 10.3389/fimmu.2025.1606607 (PMC12644057; doi:10.3389/fimmu.2025.1606607)
Supplement: Supplementary file 4 [file Table2.docx]

**Supplementary Table 2. Primers used for qRT-PCR.**

| Gene | Forward primer sequence (5‘-3’) | Reverse primer sequence (5‘-3’) | Blast |
| --- | --- | --- | --- |
| β-actin | CCCAGCACAATGAAGATCAAGATCAT | ATCTGCTGGAAGGTGGACAGCGA | <https://www.ncbi.nlm.nih.gov/tools/primer-blast/primertool.cgi?ctg_time=1752240720&job_key=mZNGOe6v4wfEOfk89FzdDo5HzDyjVNchog> |
| QPCT | AAATTGCAGAAGGCACCAGT | CTGAATTCGCTGCATGATGT | <https://www.ncbi.nlm.nih.gov/tools/primer-blast/primertool.cgi?ctg_time=1752240768&job_key=DwXR3safyzfsDVsIVmh_OixzbggBYHUVAA> |
| ARHGEF37 | TTCAGAAGAAAACCTGAACCTGC | ACGTGTCCACCAGTAGCAAG | <https://www.ncbi.nlm.nih.gov/tools/primer-blast/primertool.cgi?ctg_time=1752240829&job_key=7uQxTpmvlAezOY48g1yqDvlHuzzUVKAh1Q> |
| FLNC | CATCTACTACACAGCGCCCG | CTGTGGCACTTCAGAGGGC | <https://www.ncbi.nlm.nih.gov/tools/primer-blast/primertool.cgi?ctg_time=1752178166&job_key=d32ppoOijgqpMB41E1U6B2lOKzVEXTAoRQ> |
| LGALS7 | TAAACCTGCTATGCGGCGAG | TGCCTTGCTGTTTGGTGTTG | <https://www.ncbi.nlm.nih.gov/tools/primer-blast/primertool.cgi?ctg_time=1752177298&job_key=MDrvkJN7ntO57YToiYig2vOTsejegKr13w> |

The following PCR amplification procedure was used: Using a Bio-Rad sequence detector, the following conditions were used: 95 °C for 3 min, 40 cycles at 95 °C for 10 s, 60 °C for 20 s, and 72 °C for 20 s. The statistical evaluation of mRNA levels was conducted using the mean normalized cycle threshold (ΔΔCt) with β-actin serving as the internal control.
