# Supplementary material for: Mitogen-activated protein kinase pathway and four genes involved in the development of benign prostatic hyperplasia: in vivo and vitro validation
Source: Front Immunol. 2025 Nov 11;16:1606607. doi: 10.3389/fimmu.2025.1606607 (PMC12644057; doi:10.3389/fimmu.2025.1606607)
Supplement: Supplementary file 5 [file Table3.docx]

| Antigens | Supplier | Species antibodies | Dilution used |
| --- | --- | --- | --- |
| LGALS7 | 16730-1-AP | Rabbit | 1:200 |
| QPCT | A6711 | Rabbit | 1:200 |
| ARHGEF37 | A8490 | Rabbit | 1:200 |
| FLNC | AB244284 | Rabbit | 1:400 |

**Supplementary Table 3. List of primary antibodies.**

**Secondary antibodies:**

Supplier: ab205718.

Dilution used: 1:2000.
