# Supplementary material for: Mitogen-activated protein kinase pathway and four genes involved in the development of benign prostatic hyperplasia: in vivo and vitro validation
Source: Front Immunol. 2025 Nov 11;16:1606607. doi: 10.3389/fimmu.2025.1606607 (PMC12644057; doi:10.3389/fimmu.2025.1606607)
Supplement: Supplementary file 6 [file Table4.docx]

**Supplementary Table 4. Quantitative differential results of down-regulated proteins and protein-coding genes for comparison group.**

| **Protein** | **Description** | **P value** | **Gene symbol** | **log2FC** |
| --- | --- | --- | --- | --- |
| Q9ET32 | Histo-blood group ABO system transferase 1 | 2.67E-07 | Abo | -2.366304527 |
| Q8CFC4 | Histo-blood group ABO system transferase 2 | 3.73E-07 | Abo2 | -2.35226638 |
| Q9Z1L0 | Phosphatidylinositol 4,5-bisphosphate 3-kinase catalytic subunit beta isoform | 2.87E-06 | Pik3cb | -1.207590538 |
| O08836 | Immunoglobulin-binding protein 1 | 5.34E-06 | Igbp1 | -1.681393773 |
| Q6EV70 | GDP-fucose protein O-fucosyltransferase 1 | 4.90E-05 | Pofut1 | -1.20046934 |
| D4A3P1 | Ubiquilin-4 | 9.22E-05 | Ubqln4 | -1.345710282 |
| D3ZWR1 | 5'(3')-deoxyribonucleotidase, cytosolic type | 0.000160247 | Nt5c | -1.202960741 |
| Q63525 | Nuclear migration protein nudC | 0.000232959 | Nudc | -1.424620276 |
| Q5XI55 | Peptide-N(4)-(N-acetyl-beta-glucosaminyl)asparagine amidase | 0.000264324 | Ngly1 | -1.048740957 |
| D3ZNK1 | Metaxin-3 | 0.000297999 | Mtx3 | -2.391031392 |
| A0A0G2K402 | Myelin expression factor 2 | 0.000302256 | Myef2 | -1.182251574 |
| P20611 | Lysosomal acid phosphatase | 0.000351601 | Acp2 | -1.525920111 |
| P25093 | Fumarylacetoacetase | 0.000375513 | Fah | -1.068890177 |
| Q66HG4 | Aldose 1-epimerase | 0.000419787 | Galm | -1.340411615 |
| Q5HZA9 | Transmembrane protein 126A | 0.000443945 | Tmem126a | -1.110203035 |
| Q8CG45 | Aflatoxin B1 aldehyde reductase member 2 | 0.000502238 | Akr7a2 | -1.380090854 |
| Q5MYT7 | 2'-5'-oligoadenylate synthase 3 | 0.000519849 | Oas3 | -3.945382808 |
| Q32KJ6 | N-acetylgalactosamine-6-sulfatase | 0.00058097 | Galns | -1.152969076 |
| Q9R1B | Mitochondrial import inner membrane translocase subunit Tim10 B | 0.000638258 | Timm10b | -2.140240568 |
| Q99068 | Alpha-2-macroglobulin receptor-associated protein | 0.000659772 | Lrpap1 | -1.097697826 |
| D4A604 | GPI transamidase component PIG-T | 0.000787521 | Pigt | -1.70798103 |
| D4A4S6 | Olfactory receptor 1L4 | 0.0008138 | Olr1507 | -1.019918763 |
| D4ACK7 | Metal transporter CNNM3 | 0.000909569 | Cnnm3 | -1.37065794 |
| B0BNB5 | Nucleoporin Nup43 | 0.000967121 | Nup43 | -1.281923537 |
| Q9ER28 | Cytochrome b-245 heavy chain | 0.000986579 | Cybb | -3.358513559 |
| Q2TGK3 | Palmitoyltransferase ZDHHC3 | 0.001013927 | Zdhhc3 | -1.330963944 |
| F1LXT8 | Dynein heavy chain 6, axonemal | 0.001028754 | Dnah6 | -1.394264505 |
| Q4VBH2 | CCA tRNA nucleotidyltransferase 1, mitochondrial | 0.001128503 | Trnt1 | -1.017370474 |
| D3ZJF9 | Alpha-galactosidase A | 0.001199314 | Gla | -1.469406795 |
| P11466 | Peroxisomal carnitine O-octanoyltransferase | 0.001303208 | Crot | -1.01473542 |
| P17164 | Tissue alpha-L-fucosidase | 0.001410508 | Fuca1 | -1.102546409 |
| P31503 | POU domain, class 2, transcription factor 1 (Fragment) | 0.001436091 | Pou2f1 | -1.193771846 |
| B1WBY8 | Histone deacetylase 2 | 0.001438916 | Hdac2 | -1.699068236 |
| D4A4J0 | FACT complex subunit SPT16 | 0.001440922 | Supt16h | -1.143661852 |
| D4AC65 | Cytochrome c oxidase assembly factor 7 | 0.00149402 | Coa7 | -1.543194668 |
| Q8CHJ1 | Phosphatidylinositol glycan anchor biosynthesis class U protein | 0.001611432 | Pigu | -1.175396867 |
| Q99M63 | WD40 repeat-containing protein SMU1 | 0.001829725 | Smu1 | -1.546925242 |
| B2RYP8 | Gamma-tubulin complex component 2 | 0.001913728 | Tubgcp2 | -1.127145582 |
| D4AE02 | Protein FAM98B | 0.001985884 | Fam98b | -1.159046629 |
| Q5UT80 | Rano class II histocompatibility antigen, B alpha chain | 0.002285722 | BA1 | -2.700035371 |
| D3ZV54 | Periodic tryptophan protein 2 homolog | 0.002306032 | Pwp2 | -1.056900515 |
| Q64566 | Calcium-transporting ATPase type 2C member 1 | 0.002404464 | Atp2c1 | -1.171374648 |
| F1LQI1 | Hydroxyacylglutathione hydrolase, mitochondrial | 0.002435004 | Hagh | -1.159888473 |
| P97612 | Fatty-acid amide hydrolase 1 | 0.002513986 | Faah | -1.312263509 |
| Q9JJ46 | 3-beta-hydroxysteroid-Delta(8),Delta(7)-isomerase | 0.002539088 | Ebp | -1.258941167 |
| A0A0G2K4N5 | 25-hydroxycholesterol 7-alpha-hydroxylase (Fragment) | 0.002682935 | Cyp7b1 | -2.851668601 |
| D4A626 | Calmin | 0.00270356 | Clmn | -1.265569948 |
| O88588 | Phosphofurin acidic cluster sorting protein 1 | 0.002705607 | Pacs1 | -1.013685438 |
| B5DFG9 | Phosphoinositide 3-kinase adapter protein 1 | 0.002846045 | Pik3ap1 | -1.402393157 |
| F1LR52 | Multidrug resistance-associated protein 4 | 0.00286416 | Abcc4 | -1.174278097 |
| D3ZR49 | Mannosyl-oligosaccharide 1,2-alpha-mannosidase IB | 0.002918819 | Man1a2 | -1.315137782 |
| Q05820 | Putative lysozyme C-2 | 0.002932792 | Lyz2 | -1.107443515 |
| Q6AYS4 | Plasma alpha-L-fucosidase | 0.002994721 | Fuca2 | -1.004561584 |
| Q68FU7 | Ubiquinone biosynthesis monooxygenase COQ6, mitochondrial | 0.003032666 | Coq6 | -1.232640845 |
| O70489 | Lysosomal thioesterase PPT2 | 0.003210834 | Ppt2 | -1.166297976 |
| A3KNA0 | Intron-binding protein aquarius | 0.003325561 | Aqr | -1.422907415 |
| Q5U3Z3 | Isochorismatase domain-containing protein 2 | 0.003582742 | Isoc2 | -1.255490251 |
| P45479 | Palmitoyl-protein thioesterase 1 | 0.003989207 | Ppt1 | -1.20345394 |
| A0A0G2K2P4 | Cytochrome P450 2F3 | 0.004046893 | Cyp2t1 | -1.558962849 |
| D3ZEA0 | Fibronectin type-III domain-containing protein 3A | 0.004179181 | Fndc3a | -1.527069578 |
| D3ZMN2 | Histone PARylation factor 1 | 0.00435695 | Hpf1 | -1.481637611 |
| D3ZGW2 | AP-1 complex subunit gamma-like 2 | 0.004458808 | Ap1g2 | -2.000478044 |
| A0A0G2K1B6 | Nuclear factor 1 X-type | 0.004512157 | Nf1x | -1.581568877 |
| Q5U204 | Ragulator complex protein LAMTOR3 | 0.004583281 | Lamtor3 | -1.40449796 |
| D3ZUX7 | Acyl-CoA synthetase family member 3, mitochondrial | 0.004616647 | Acsf3 | -1.110414425 |
| D3ZU51 | Ribonuclease P protein subunit p30 | 0.004692256 | Rpp30 | -1.114478815 |
| Q62931 | Golgi SNAP receptor complex member 1 | 0.004771188 | Gosr1 | -1.21122265 |
| Q9R1T3 | Cathepsin Z | 0.005076335 | Ctsz | -1.50494473 |
| D4AA35 | N-acetylserotonin O-methyltransferase-like protein | 0.005468819 | Asmtl | -1.722855871 |
| F1LQC8 | Cyclin-dependent kinase 7 | 0.005547377 | Cdk7 | -1.107111895 |
| P0C2C4 | 39S ribosomal protein L10, mitochondrial | 0.005637921 | Mrpl10 | -1.381776029 |
| Q9ER31 | Ectonucleoside triphosphate diphosphohydrolase 6 | 0.005697525 | Entpd6 | -1.439369718 |
| Q5EB90 | DNA-directed RNA polymerase II subunit RPB3 | 0.005781017 | Polr2c | -1.291520153 |
| D3ZKR8 | Protein kish-A | 0.006019328 | Tmem167a | -1.437788348 |
| A0A096MKF8 | Mediator of RNA polymerase II transcription subunit 12 | 0.006106643 | Med12 | -1.077606063 |
| Q5I0D7 | Xaa-Pro dipeptidase | 0.006342059 | Pepd | -1.455561553 |
| F1LRQ6 | Cell division cycle protein 23 homolog | 0.006370531 | Cdc23 | -1.142493045 |
| B1WBY7 | Erlin-1 | 0.006399167 | Erlin1 | -1.034569693 |
| Q6MGB6 | E3 ubiquitin-protein ligase RING1 | 0.006441878 | Ring1 | -1.030515569 |
| B5DF79 | Ribonuclease T2-B | 0.006619189 | Rnaset2 | -1.019795102 |
| P24050 | 40S ribosomal protein S5 | 0.006737269 | Rps5 | -1.687822979 |
| B1WC35 | Transmembrane protein 161A | 0.006813667 | Tmem161a | -1.270966823 |
| B5DEI2 | L-amino-acid oxidase | 0.007043545 | Lao1 | -5.548708727 |
| P84039 | Ectonucleotide pyrophosphatase/phosphodiesterase family member 5 | 0.007046392 | Enpp5 | -1.019806366 |
| Q66H12 | Alpha-N-acetylgalactosaminidase | 0.007359307 | Naga | -1.001367525 |
| Q6TEK3 | Vitamin K epoxide reductase complex subunit 1-like protein 1 | 0.008373993 | Vkorc1l1 | -1.240350192 |
| F1LQ48 | Heterogeneous nuclear ribonucleoprotein L | 0.008559889 | Hnrnpl | -1.196925367 |
| Q5RKH2 | Galactokinase | 0.008606089 | Galk1 | -1.087916172 |
| D3ZML4 | TraB domain-containing protein | 0.008680408 | Trabd | -1.176303927 |
| D4A9A3 | Centromere protein V | 0.008860324 | Cenpv | -1.10560423 |
| D3ZUL8 | Zinc finger CCHC domain-containing protein 8 | 0.008956269 | Zcchc8 | -1.294116358 |
| A0A0G2JV51 | RNA cytidine acetyltransferase | 0.009140091 | Nat10 | -1.208310832 |
| P04961 | Proliferating cell nuclear antigen | 0.009542658 | Pcna | -1.268102736 |
| Q63619 | 5-demethoxyubiquinone hydroxylase, mitochondrial (Fragment) | 0.010495502 | Coq7 | -1.045902276 |
| B5DEL5 | Kelch-like protein 9 | 0.010560479 | Klhl9 | -2.167040671 |
| A0A0G2K1Q9 | Band 4.1-like protein 3 | 0.010640402 | Epb41l3 | -1.223824042 |
| P62078 | Mitochondrial import inner membrane translocase subunit Tim8 B | 0.01072462 | Timm8b | -1.260168741 |
| Q9Z339 | Glutathione S-transferase omega-1 | 0.010854622 | Gsto1 | -1.056970074 |
| A0A0G2JVH5 | Helicase-like transcription factor | 0.011387733 | Hltf | -1.060750584 |
| P60825 | Cold-inducible RNA-binding protein | 0.011405163 | Cirbp | -1.721125169 |
| B1H2A6 | Fragile X mental retardation syndrome-related protein 2 | 0.011483251 | Fxr2 | -1.358303278 |
| A0A0G2K8M7 | Tumor protein D53 | 0.011873678 | Tpd52l1 | -1.230860713 |
| F1M365 | Integrator complex subunit 9 | 0.011881596 | Ints9 | -1.132096765 |
| P30904 | Macrophage migration inhibitory factor | 0.012118214 | Mif | -1.546162479 |
| Q68FX7 | THO complex subunit 5 homolog | 0.012169681 | Thoc5 | -1.200305971 |
| A0A0G2KAP1 | ERO1-like protein beta | 0.012306044 | Ero1b | -2.329251189 |
| F1LR42 | RUN and FYVE domain-containing protein 1 | 0.012404571 | Rufy1 | -1.252764457 |
| Q4QQW4 | Histone deacetylase 1 | 0.012855844 | Hdac1 | -1.098390432 |
| Q5HZE4 | Methylthioribose-1-phosphate isomerase | 0.01377589 | Mri1 | -1.492037601 |
| D3ZHR2 | ATP-binding cassette sub-family D member 1 | 0.013863629 | Abcd1 | -1.169829759 |
| D3ZJF7 | Telomeric repeat-binding factor 2 | 0.014160995 | Terf2 | -1.102558187 |
| Q9EQN5 | DNA-binding protein SMUBP-2 | 0.014669742 | Ighmbp2 | -1.710398899 |
| A0A0G2K3D7 | Zinc finger and BTB domain-containing protein 1 | 0.014718589 | Zbtb1 | -1.767682696 |
| P0C588 | Metal transporter CNNM4 | 0.016148565 | Cnnm4 | -1.264795394 |
| Q5U1W6 | MICOS complex subunit Mic27 | 0.017274947 | Apool | -1.313515948 |
| Q9EQV6 | Tripeptidyl-peptidase 1 | 0.018150632 | Tpp1 | -1.262356415 |
| Q498C9 | BUB3-interacting and GLEBS motif-containing protein ZNF207 | 0.018520314 | Zfp207 | -1.299028299 |
| B4F7C2 | Tubulin beta-4A chain | 0.019714322 | Tubb4a | -1.597983329 |
| Q9QZI7 | tRNA selenocysteine 1-associated protein 1 | 0.020664811 | Trnau1ap | -1.182551342 |
| D3ZEL0 | Pre-mRNA-splicing factor CWC22 homolog | 0.020986329 | LOC500684 | -1.193112246 |
| A9CMA7 | Cyclin-T2 | 0.021613156 | Ccnt2 | -1.399893719 |
| D3ZG54 | WW domain-containing oxidoreductase | 0.022420059 | Wwox | -1.027335373 |
| Q5XIB2 | Peptidyl-prolyl cis-trans isomerase CWC27 homolog | 0.022557094 | Cwc27 | -1.025822005 |
| D4A2N2 | Type II inositol 1,4,5-trisphosphate 5-phosphatase | 0.022793367 | Inpp5b | -1.261830648 |
| D3ZD09 | Cytochrome c oxidase subunit 6B1 | 0.023158036 | Cox6b1 | -1.884439384 |
| D3Z9U8 | Protein S100-A7 | 0.023670812 | S100a7l2 | -2.780097081 |
| Q562C7 | Pumilio homolog 3 | 0.024269602 | Pum3 | -1.956109647 |
| D3ZUC2 | Putative helicase MOV-10 | 0.024277725 | Mov10 | -1.884518144 |
| P16303 | Carboxylesterase 1D | 0.024490864 | Ces1d | -1.164307622 |
| G3V757 | Alpha-(1,3)-fucosyltransferase 4 | 0.025135912 | Fut4 | -1.025176933 |
| Q3MHU5 | Reticulophagy regulator 2 | 0.025410623 | Retreg2 | -1.072556996 |
| D3ZJB2 | Glycoprotein integral membrane protein 1 | 0.025894903 | Ginm1 | -1.104529391 |
| A0A0G2JU45 | tRNA (guanine(37)-N1)-methyltransferase | 0.026237744 | Trmt5 | -1.129215504 |
| Q9JKW1 | Mitochondrial import inner membrane translocase subunit Tim22 | 0.026847983 | Timm22 | -1.109054337 |
| Q5FVG2 | Band 4.1-like protein 5 | 0.027234383 | Epb41l5 | -1.089806566 |
| A0A140TAA1 | Immunoglobulin superfamily member 8 | 0.028077465 | Igsf8 | -1.079822399 |
| Q4V7F5 | PIH1 domain-containing protein 1 | 0.029739141 | Pih1d1 | -1.417045219 |
| M0R3V4 | Myeloid-derived growth factor | 0.029749046 | Mydgf | -1.195680107 |
| Q9WVK3 | Peroxisomal trans-2-enoyl-CoA reductase | 0.029841468 | Pecr | -1.66305271 |
| P70584 | Short/branched chain specific acyl-CoA dehydrogenase, mitochondrial | 0.030552835 | Acadsb | -1.824907719 |
| P50878 | 60S ribosomal protein L4 | 0.031145478 | Rpl4 | -1.046453155 |
| Q5I0H9 | Protein disulfide-isomerase A5 | 0.032589783 | Pdia5 | -1.094071749 |
| Q5XIE0 | Acidic leucine-rich nuclear phosphoprotein 32 family member E | 0.035341383 | Anp32e | -1.191581368 |
| D3ZY44 | 28S ribosomal protein S2, mitochondrial | 0.035374695 | Mrps2 | -1.070369072 |
| P18211 | Rano class II histocompatibility antigen, D-1 beta chain | 0.035914131 | RT1-Db1 | -1.703162531 |
| D4A997 | HIV Tat-specific factor 1 homolog | 0.037091717 | Htatsf1 | -1.376832522 |
| Q78EG7 | Protein tyrosine phosphatase type IVA 1 | 0.037776726 | Ptp4a1 | -1.365659529 |
| G3V6P6 | RNA-binding protein 3 | 0.038475525 | Rbm3 | -1.138723307 |
| D3ZV30 | DNA-directed RNA polymerase III subunit RPC2 | 0.040441062 | Polr3b | -1.158212038 |
| Q64380 | Sarcosine dehydrogenase, mitochondrial | 0.042230814 | Sardh | -1.411293912 |
| D3ZQ77 | Keratinocyte-associated transmembrane protein 2 | 0.043257049 | RGD1310352 | -1.021264919 |
| B0BNI2 | WD repeat-containing protein 74 | 0.043720465 | Wdr74 | -1.618230845 |
| B2GUX7 | Protein CREG1 | 0.046046355 | Creg1 | -1.210580318 |
| F1LY14 | Anoctamin-9 | 0.046483393 | Ano9 | -2.455222703 |
| A0A0G2K1Q8 | ATP-binding cassette sub-family A member 3 | 0.046946547 | Abca3 | -1.006997935 |
| Q64550 | UDP-glucuronosyltransferase 1-1 | 0.047223494 | Ugt1a1 | -1.022406979 |
|  |  |  |  |  |
