# Supplementary material for: Mitogen-activated protein kinase pathway and four genes involved in the development of benign prostatic hyperplasia: in vivo and vitro validation
Source: Front Immunol. 2025 Nov 11;16:1606607. doi: 10.3389/fimmu.2025.1606607 (PMC12644057; doi:10.3389/fimmu.2025.1606607)
Supplement: Supplementary file 7 [file Table5.docx]

**Supplementary Table 5. GO analysis of differentially proteins in BPH and sham rats according to biological progress, cellular component and molecular function.**

| **Category** | **GO term** | **Number of EDPs** | **Protein members** |
| --- | --- | --- | --- |
| biological process | behavior | 3 | P45479, A0A0G2K4N5, B1WBY8 |
| biological process | biological adhesion | 11 | O08701, P23562, P70490, P97590, Q5FVG2, Q9Z1L0, A0A0G2K3D7, D3ZBN3, D3ZCV0, D3ZEA0, Q5UT80 |
| biological process | biological regulation | 110 | A1IGU3, F1LQ48, O08701, O08836, O70597, O88588, P04961, P07150, P0C588, P15205, P16303, P23562, P24050, P30904, P31214, P31503, P32232, P45479, P50878, P60825, P70490, P97612, Q4QQW4, Q4V7F5, Q562C7, Q5FVG2, Q5I0H9, Q5MYT7, Q5U204,Q5U3Z3,Q5XID7, Q5XIE0, Q62662, Q62931, Q63279, Q63619, Q64380, Q64550, Q68FX7, Q6AYC4, Q6AYS4, Q6EV70, Q6MGB6, Q78EG7,Q8CFC4, Q8CHJ1, Q8CJ11, Q99068, Q99M63, Q9EQN5, Q9EQV6, Q9QZI7, Q9R1T3, Q9R1T5, Q9Z1L0, Q9Z339, A0A096MKF8, A0A0G2JV51, A0A0G2JVH5, A0A0G2JWD0, A0A0G2JY11, A0A0G2K1B6, A0A0G2K1Q9, A0A0G2K3D7, A0A0G2K402, A0A0G2K4N5, A0A0G2K8M7, A0A0G2KAP1, A0A140TAA, A9CMA7, B1H2A6, B1WBY7, B1WBY8, B1WC35, B2GUX7, B2RYP8, B4F7C2, B5DFG9, D3Z9U8, D3ZBN3, D3ZCR4, D3ZCV0, D3ZG54, D3ZJF7, D3ZMN2, D3ZPN5, D3ZUC2, D3ZV30, D3ZW27, D4A2N2, D4A3P1, D4A4J0, D4A4S6, D4A626, D4A997, D4A9A3, D4AE02, F1LQC8, F1LR42, F1LR52, F1LRH4, F1LRQ6, F1LY14, G3V6P6, M0R3V4, M0R9N6, Q498C9, Q5EB90, Q5UT80, Q9ER28 |
| biological process | cell proliferation | 18 | O08701, P23562, P30904, P70490, Q4QQW4, Q62662, Q8CFC4, Q9R1T3, Q9Z1L0, A0A0G2K4N5, B1WBY8, D3ZBN3, D3ZW27, D4A626, D4AE02, F1LR52, F1LRH4, M0R3V4 |
| biological process | cellular component organization or biogenesis | 57 | D3ZHR2, O70597, P04961, P07150, P15205, P16303, P20611, P23562, P24050, P30904, P45479, P50878, P60825, P62078, P70490, Q4QQW4, Q4V7F5, Q5FVG2, Q5XIE0, Q62931, Q63279, Q63619, Q68FX7, Q6AYC4, Q6IFV1, Q6MGB6, Q99068, Q9EQN5, Q9EQV6, Q9JKW1, Q9R1T3, A0A0G2JV51, A0A0G2JVH5, A0A0G2K1Q9, A0A0G2K3D7, A0A0G2KAP1, B0BNI2, B1WBY8, B2RYP8, B4F7C2, D3ZBN3, D3ZCV0, D3ZJF7, D3ZNK1, D3ZV54, D4A3P1, D4A4J0, D4A626, D4A9A3, F1LR42, F1LR52, F1LRH4, F1LRQ6, F1LXT8, F1LY14, Q498C9, Q5U1W6 |
| biological process | cellular process | 161 | A1IGU3, D3ZHA0, D3ZHR2, F1LQ48, O08701, O08836, O70597, P04961, P07150, P0C2C4, P11466, P15205, P16303, P20611, P23562, P24050, P24464, P25093, P30904, P31214, P31503, P32232, P45479, P50878, P60825, P62078, P70490, P70584, P84039, P97590, P97612, Q05820, Q4QQW4, Q4TU93, Q4V7F5, Q562C7, Q5FVG2, Q5HZE4, Q5I0H9, Q5MYT7, Q5U204, Q5XI55, Q5XIB2, Q5XID7, Q5XIE0, Q62662, Q62931, Q63279, Q63525, Q63619, Q64380, Q64550, Q66H12, Q68FU7, Q68FX7, Q6AYC4, Q6EV70, Q6IFV1, Q6MGB6, Q6TEK3, Q78EG7, Q8CFC4, Q8CG45, Q8CHJ1, Q8CJ11, Q99068, Q99M63, Q9EQN5, Q9EQV6, Q9ET32, Q9JKW1, Q9QZI7, Q9R1T3, Q9R1T5, Q9WVK3, Q9Z122, Q9Z1L0, Q9Z339, A0A096MKF8, A0A0G2JU12, A0A0G2JU45, A0A0G2JV51, A0A0G2JVH5, A0A0G2JWD0, A0A0G2JY11, A0A0G2K1B6, A0A0G2K1Q9, A0A0G2K3D7, A0A0G2K402, A0A0G2K4N5, A0A0G2K8M7, A0A0G2KAP1, A0A140TAA1, A3KNA0, A9CMA7, B0BNI2, B1H2A6, B1WBY7, B1WBY8, B1WC35, B2GUX7, B2RYP8, B4F7C2, B5DEI2, B5DEL5, B5DF79, B5DFG9, D3Z9U8, D3ZBN3, D3ZCR4, D3ZCV0, D3ZEA0, D3ZG54, D3ZJF7, D3ZKR8, D3ZMN2, D3ZNK1, D3ZPN5, D3ZR49, D3ZU51, D3ZUC2, D3ZUL8, D3ZUX7, D3ZV30, D3ZV54, D3ZW27, D3ZWR1, D3ZY44, D4A2K1, D4A2N2, D4A3P1, D4A4J0, D4A4S6, D4A604, D4A626, D4A997, D4A9A3, D4A9Q5, D4AC85, D4AE02, F1LQC8, F1LQI1, F1LR42, F1LR52, F1LRH4, F1LRQ6, F1LXT8, F1LY14, F1M365, G3V6P6, G3V757, M0R3V4, M0R9N6, Q2TGK3, Q498C9, Q4VBH2, Q5EB90, Q5RKH2, Q5U1W6, Q5UT80, Q9ER28 |
| biological process | detoxification | 1 | Q9Z339 |
| biological process | developmental process | 65 | D3ZHA0, O08701, O70597, P04961, P07150, P0C588, P15205, P16303, P20611, P24464, P30904, P31214, P31503, P32232, P45479, P50878, P70490, Q4QQW4, Q4TU93, Q4V7F5, Q5FVG2, Q5HZA9, Q62662, Q63279, Q63525, Q63619, Q64550, Q68FX7, Q6EV70, Q6IFV1, Q6MGB6, Q78EG7, Q9EQN5, Q9EQV6, Q9JJ46, Q9R1T3, Q9R1T5, Q9Z1L0, A0A096MKF8, A0A0G2JWD0, A0A0G2K1B6, A0A0G2K1Q9, A0A0G2K3D7, A0A0G2K402, A0A0G2K4N5, A9CMA7, B0BNI2, B1WBY8, D3ZBN3, D3ZCV0, D3ZD09, D3ZEA0, D3ZG54, D3ZJF7, D3ZR49, D3ZW27, D4A2N2, D4A604, D4A626, F1LRH4, G3V8C0, M0R3V4, Q5EB90, Q5UT80, Q9ER28 |
| biological process | growth | 8 | P15205, P45479, P50878, P60825, Q5FVG2, A0A096MKF8, A0A0G2K1Q9, B2GUX7 |
| biological process | immune system process | 21 | O08701, O08836, P07150, P18211, P23562, P30904, Q5MYT7, Q62662, Q68FX7, Q9JJ46, Q9Z1L0, A0A0G2K3D7, A0A0G2K4N5, B5DFG9, D3ZBN3, D3ZV30, D3ZW27, G3V8C0, M0R9N6, Q5UT80, Q9ER28 |
| biological process | localization | 52 | D3ZHR2, O08701, O08836, O88588, P07150, P0C588, P11466, P15205, P23562, P24050, P30904, P45479, P62078, P70490, Q4TU93, Q4V7F5, Q5FVG2, Q5U204, Q5XID7, Q5XIE0, Q62662, Q62931, Q63525, Q68FX7, Q78EG7, Q99068, Q9JKW1, Q9R1B1, Q9Z1L0, Q9Z339, A0A0G2K1Q8, A0A0G2K1Q9, A0A0G2K4N5, A0A140TAA1, B4F7C2, D3ZBN3, D3ZCV0, D3ZGW2, D3ZJF7, D3ZKR8, D3ZNK1, D4A2N2, D4A9A3, D4ACK7, F1LR42, F1LR52, F1LRH4, F1LRQ6, F1LXT8, F1LY14, G3V8C0, Q2TGK3 |
| biological process | locomotion | 12 | P30904, Q5FVG2, Q62662, Q78EG7, Q9Z1L0, A0A0G2K4N5, A0A140TAA1, D3ZBN3, D4A2N2, D4A9A3, F1LRH4, F1LXT8 |
| biological process | metabolic process | 129 | D3ZHR2, F1LQ48, O08701, O08836, P04961, P0C2C4, P11466, P16303, P17164, P24050, P24464, P25093, P30904, P31214, P31503, P32232, P45479, P50878, P60825, P70584, P84039, P97612, Q05820, Q32KJ6, Q4QQW4, Q4TU93, Q4V7F5, Q562C7, Q5FVG2, Q5HZE4, Q5I0D7, Q5MYT7, Q5U204, Q5U3Z3, Q5XI55, Q5XID7, Q5XIE0, Q62662, Q63619, Q64380, Q64550\|UD11, Q66H12, Q66HG4, Q68FU7, Q68FX7, Q6AYS4, Q6EV70, Q6MGB6, Q6TEK3, Q8CFC4, Q8CG45, Q8CHJ1, Q99068, Q99M63, Q9EQN5, Q9EQV6, Q9ET32, Q9JJ46, Q9QZI7, Q9R1T3, Q9R1T5, Q9WVK3, Q9Z122, Q9Z1L0, Q9Z339, A0A096MKF8, A0A0G2JU12, A0A0G2JU45, A0A0G2JV51, A0A0G2JVH5, A0A0G2K1B6, A0A0G2K3D7, A0A0G2K402, A0A0G2K4N5, A0A0G2K8M7, A0A0G2KAP1, A3KNA0, A9CMA7, B0BNI2, B1H2A6, B1WBY7, B1WBY8, B1WC35, B2GUX7, B4F7C2, B5DEI2, B5DEL5, B5DF79, D3ZBN3, D3ZCR4, D3ZG54, D3ZJF7, D3ZJF9, D3ZMN2, D3ZPN5, D3ZR49, D3ZU51, D3ZUC2, D3ZUL8, D3ZUX7, D3ZV30, D3ZV54, D3ZW27, D3ZWR1, D3ZY44, D4A2K1, D4A2N2, D4A3P1, D4A4J0, D4A604, D4A997, D4A9A3, D4A9Q5, D4AC85, D4AE02, F1LQC8, F1LQI1, F1LRQ6, F1M365, G3V6P6, G3V757, M0R3V4, M0R9N6, Q2TGK3, Q498C9, Q4VBH2, Q5EB90, Q5RKH2, Q9ER28 |
| biological process | multi organism process | 22 | O08701, P24050, P30904, P32232, P70490, Q05820, Q4QQW4, Q5MYT7, Q64550, Q68FX7, Q6AYS4, Q8CFC4, Q9ET32, A0A0G2JU12, A9CMA7, D3ZBN3, D3ZEA0, D3ZV30, D4A2N2, F1LQI1, M0R9N6, Q5EB90 |
| biological process | multicellular organismal process | 66 | O08701, P04961, P07150, P0C588, P15205, P16303, P20611, P24464, P30904, P31214, P31503, P32232, P45479, P50878, P62078, P70490, P97612, Q4QQW4, Q4TU93, Q5FVG2, Q5HZA9, Q5I0D7, Q63279, Q63619, Q64550, Q68FX7, Q6EV70, Q6IFV1, Q6MGB6, Q78EG7, Q99068, Q9EQN5, Q9EQV6, Q9JJ46, Q9R1T3, Q9R1T5, Q9Z1L0, A0A096MKF8, A0A0G2JWD0, A0A0G2K1B6, A0A0G2K1Q9, A0A0G2K3D7, A0A0G2K402, A0A0G2K4N5, A9CMA7, B0BNI2, B1WBY8, D3ZBN3, D3ZCV0, D3ZD09, D3ZEA0, D3ZG54, D3ZJF7, D3ZR49, D3ZV30, D4A2N2, D4A4S6, D4A604, D4A626, F1LQI1, F1LRH4, G3V8C0, M0R3V4, Q5EB90, Q5UT80, Q9ER28 |
| biological process | negative regulation of biological process | 52 | F1LQ48, O08701, O08836, P04961, P15205, P24050, P30904, P31503, P32232, P45479, P60825, Q4QQW4, Q4V7F5, Q5FVG2, Q5MYT7, Q62662, Q63619, Q64550, Q68FX7, Q6AYC4, Q6MGB6, Q8CFC4, Q99068, Q9EQN5, Q9R1T3, Q9Z339, A0A096MKF8, A0A0G2JV51, A0A0G2K1B6, A0A0G2K3D7, A0A0G2K402, A0A0G2K4N5, B1H2A6, B1WBY7, B1WBY8, B1WC35, B4F7C2, B5DFG9, D3ZBN3, D3ZCV0, D3ZG54, D3ZJF7, D3ZPN5, D3ZUC2, D4A3P1, D4A626, F1LY14, G3V6P6, M0R3V4, M0R9N6, Q498C9, Q5EB90 |
| biological process | positive regulation of biological process | 55 | F1LQ48, O08701, O08836, P04961, P15205, P23562, P30904, P31503, P45479, P50878, P60825, P70490, Q4QQW4, Q4V7F5, Q5FVG2, Q5U204, Q5XID7, Q63619, Q68FX7, Q78EG7, Q8CFC4, Q9R1T3, Q9R1T5, Q9Z1L0, Q9Z339, A0A096MKF8, A0A0G2JVH5, A0A0G2JWD0, A0A0G2K1B6, A0A0G2K3D7, A0A0G2K4N5, A0A0G2K8M7, A9CMA7, B1WBY7, B1WBY8, B1WC35, B5DFG9, D3ZBN3, D3ZCR4, D3ZCV0, D3ZG54, D3ZJF7, D3ZV30, D3ZW27, D4A4J0, D4A9A3, D4AE02, F1LQC8, F1LR52, F1LRH4, G3V6P6, M0R3V4, Q5EB90, Q5UT80, Q9ER28 |
| biological process | presynaptic process involved in chemical synaptic transmission | 1 | P45479 |
| biological process | regulation of biological process | 102 | A1IGU3, F1LQ48, O08701, O08836, O70597, P04961, P07150, P15205, P16303, P23562, P24050, P30904, P31503, P32232, P45479, P50878, P60825, P70490, Q4QQW4, Q4V7F5, Q562C7, Q5FVG2, Q5I0H9, Q5MYT7, Q5U204, Q5XID7, Q5XIE0, Q62662, Q62931, Q63279, Q63619, Q64550, Q68FX7, Q6AYC4, Q6AYS4, Q6EV70, Q6MGB6, Q78EG7, Q8CFC4, Q8CHJ1, Q8CJ11, Q99068, Q99M63, Q9EQN5, Q9QZI7, Q9R1T3, Q9R1T5, Q9Z1L0, Q9Z339, A0A096MKF8, A0A0G2JV51, A0A0G2JVH5, A0A0G2JWD0, A0A0G2JY11, A0A0G2K1B6, A0A0G2K1Q9, A0A0G2K3D7, A0A0G2K402, A0A0G2K4N5, A0A0G2K8M7, A0A0G2KAP1, A0A140TAA1, A9CMA7, B1H2A6, B1WBY7, B1WBY8, B1WC35, B2GUX7, B2RYP8, B4F7C2, B5DFG9, D3ZBN3, D3ZCR4, D3ZCV0, D3ZG54, D3ZJF7, D3ZMN2, D3ZPN5, D3ZUC2, D3ZV30, D3ZW27, D4A2N2, D4A3P1, D4A4J0, D4A4S6, D4A626, D4A997, D4A9A3, D4AE02, F1LQC8, F1LR42, F1LR52, F1LRH4, F1LRQ6, F1LY14, G3V6P6, M0R3V4, M0R9N6, Q498C9, Q5EB90, Q5UT80, Q9ER28 |
| biological process | reproduction | 11 | O08701, P04961, P31214, P32232, P70490, Q63279, A0A0G2K4N5, B2RYP8, D3ZEA0, D4A2N2, F1LQI1 |
| biological process | reproductive process | 11 | O08701, P04961, P31214, P32232, P70490, Q63279, A0A0G2K4N5, B2RYP8, D3ZEA0, D4A2N2, F1LQI1 |
| biological process | response to stimulus | 74 | A1IGU3, F1LQ48, O08701, O08836, O70597, P04961, P07150, P0C588, P11466, P15205, P18211, P23562, P24464, P30904, P31214, P32232, P50878, P60825, P70490, Q05820, Q4QQW4, Q4V7F5, Q5FVG2, Q5I0H9, Q5MYT7, Q5U204, Q62662, Q63279, Q63525, Q63619, Q64550, Q68FX7, Q6AYS4, Q6EV70, Q6IFV1, Q6TEK3, Q8CFC4, Q8CHJ1, Q8CJ11, Q9ER31, Q9ET32, Q9Z1L0, Q9Z339, A0A096MKF8, A0A0G2JU12, A0A0G2JY11, A0A0G2K1Q8, A0A0G2K3D7, A0A0G2K4N5, A0A0G2K8M7, B1WBY7, B1WBY8, B1WC35, B2RYP8, B5DFG9, D3Z9U8, D3ZBN3, D3ZCV0, D3ZG54, D3ZJF7, D3ZMN2, D3ZV30, D3ZW27, D4A2N2, D4A4J0, D4A4S6, F1LQC8, F1LR52, G3V6P6, M0R3V4, M0R9N6, Q5EB90, Q5UT80, Q9ER28 |
| biological process | rhythmic process | 5 | F1LQ48, P04961, P23562, Q4QQW4, A0A0G2K4N5 |
| biological process | signaling | 35 | A1IGU3, O08836, O70597, P07150, P15205, P30904, P32232, P45479, Q4QQW4, Q4V7F5, Q5U204, Q62662, Q63279, Q6EV70, Q8CHJ1, Q8CJ11, Q9Z1L0, Q9Z339, A0A096MKF8, A0A0G2JY11, A0A0G2K4N5, A0A0G2K8M7, B1WBY7, B1WC35, B2RYP8, B5DFG9, D3ZBN3, D3ZCV0, D3ZG54, D3ZW27, D4A2N2, D4A4S6, M0R3V4, Q5EB90, Q9ER28 |
| cellular component | cell | 179 | A1IGU3, D3ZHA0, D3ZHR2, F1LQ48, O08701, O08836, O70489, O70597, O88588, P04961, P07150, P0C2C4, P0C588, P11466, P15205, P16303, P17164, P18211, P20611, P23562, P24050, P24464, P30904, P31214, P31503, P32232, P45479, P50878, P60825, P62078, P70490, P70584, P84039, P97590, P97612, Q32KJ6, Q4QQW4, Q4TU93, Q4V7F5, Q562C7, Q5BKC6, Q5FVG2, Q5HZA9, Q5HZE4, Q5I0H9, Q5MYT7, Q5U204, Q5U3Z3, Q5XI55, Q5XIB2, Q5XID7, Q5XIE0, Q62662, Q62931, Q63279, Q63525, Q63619, Q64380, Q64550, Q66H12, Q66HG4, Q68FU7, Q68FX7, Q6AYC4, Q6EV70, Q6IFV1, Q6MGB6, Q6TEK3, Q78EG7, Q8CFC4, Q8CG45, Q8CHJ1, Q8CJ11, Q99068, Q99M63, Q9EQN5, Q9EQV6, Q9ER31, Q9ET32, Q9JJ46, Q9JKW1, Q9QZI7, Q9R1B1, Q9R1T3, Q9R1T5, Q9WVK3, Q9Z122, Q9Z1L0, Q9Z339, A0A096MKF1, A0A096MKF8, A0A0G2JU12, A0A0G2JU45, A0A0G2JV51, A0A0G2JVH5, A0A0G2JWD0, A0A0G2JY11, A0A0G2K1B6, A0A0G2K1Q8, A0A0G2K1Q9, A0A0G2K3D7, A0A0G2K402, A0A0G2K4N5, A0A0G2K8M7, A0A0G2KAP1, A0A140TAA1, A3KNA0, A9CMA7, B0BNB5, B0BNI2, B1H2A6, B1WBY7, B1WBY8, B2GUX7, B2RYP8, B4F7C2, B5DEL5, B5DF79, B5DFG9, D3Z9U8, D3ZBN3, D3ZCR4, D3ZCV0, D3ZD09, D3ZEA0, D3ZG54, D3ZGW2, D3ZJF7, D3ZJF9, D3ZKR8, D3ZMN2, D3ZNK1, D3ZPN5, D3ZR49, D3ZU51, D3ZUC2, D3ZUL8, D3ZUX7, D3ZV30, D3ZV54, D3ZW27, D3ZWR1, D3ZY44, D4A2K1, D4A2N2, D4A3P1, D4A4J0, D4A4S6, D4A604, D4A626, D4A997, D4A9A3, D4A9Q5, D4AA35, D4AC65, D4ACK7, D4AE02, F1LQC8, F1LQI1, F1LR42, F1LR52, F1LRH4, F1LRQ6, F1LXT8, F1LY14, F1M365, G3V6P6, G3V757, G3V8C0, M0R3V4, M0R9N6, Q2TGK3, Q498C9, Q4VBH2, Q5EB90, Q5RKH2, Q5U1W6, Q5UT80, Q9ER28 |
| cellular component | cell junction | 13 | D3ZHA0, P07150, P15205, P23562, P24050, P50878, Q4TU93, Q5FVG2, Q5U204, A0A0G2JY11, A0A0G2K1Q9, D3ZBN3, D3ZCV0 |
| cellular component | cell part | 179 | A1IGU3, D3ZHA0, D3ZHR2, F1LQ48, O08701, O08836, O70489, O70597, O88588, P04961, P07150, P0C2C4, P0C588, P11466, P15205, P16303, P17164, P18211, P20611, P23562, P24050, P24464, P30904, P31214, P31503, P32232, P45479, P50878, P60825, P62078, P70490, P70584, P84039, P97590, P97612, Q32KJ6, Q4QQW4, Q4TU93, Q4V7F5, Q562C7, Q5BKC6, Q5FVG2, Q5HZA9, Q5HZE4, Q5I0H9, Q5MYT7, Q5U204, Q5U3Z3, Q5XI55, Q5XIB2, Q5XID7, Q5XIE0, Q62662, Q62931, Q63279, Q63525, Q63619, Q64380, Q64550, Q66H12, Q66HG4, Q68FU7, Q68FX7, Q6AYC4, Q6EV70, Q6IFV1,Q6MGB6, Q6TEK3, Q78EG7, Q8CFC4, Q8CG45, Q8CHJ1, Q8CJ11, Q99068, Q99M63, Q9EQN5, Q9EQV6, Q9ER31, Q9ET32, Q9JJ46, Q9JKW1, Q9QZI7, Q9R1B1, Q9R1T3, Q9R1T5, Q9WVK3, Q9Z122, Q9Z1L0, Q9Z339, A0A096MKF1, A0A096MKF8, A0A0G2JU12, A0A0G2JU45, A0A0G2JV51, A0A0G2JVH5, A0A0G2JWD0 , A0A0G2JY11, A0A0G2K1Q8, A0A0G2K1Q9, A0A0G2K3D7, A0A0G2K402, A0A0G2K4N5, A0A0G2K8M7, A0A0G2KAP1, A0A140TAA1\|, A3KNA0, A9CMA7, B0BNB5, B0BNI2, B1H2A6, B1WBY7, B1WBY8, B2GUX7, B2RYP8, B4F7C2, B5DEL5, B5DF79, B5DFG9, D3Z9U8, D3ZBN3, D3ZCR4, D3ZCV0, D3ZD09, D3ZEA0, D3ZG54, D3ZGW2, D3ZJF7, D3ZJF9, D3ZKR8, D3ZMN2, D3ZNK1, D3ZPN5, D3ZR49, D3ZU51, D3ZUC2, D3ZUL8\|D3ZUL8 ; D3ZUX7, D3ZV30, D3ZV54, D3ZW27, D3ZWR1, D3ZY44, D4A2K1, D4A2N2, D4A3P1, D4A4J0, D4A4S6, D4A604, D4A626, D4A997, D4A9A3, D4A9Q5, D4AA35, D4AC65, D4ACK7, D4AE02, F1LQC8, F1LQI1, F1LR42, F1LR52, F1LRH4, F1LRQ6, F1LXT8, F1LY14, F1M365, G3V6P6, G3V757, G3V8C0, M0R3V4, M0R9N6, Q2TGK3, Q498C9, Q4VBH2, Q5EB90, Q5RKH2, Q5U1W6, Q5UT80, Q9ER28 |
| cellular component | extracellular region | 62 | F1LQ48, O70489, P04961, P17164, P20611, P23562, P24050, P24464, P25093, P30904, P45479, P50878, P62078, P70490, P70584, P84039, P97590, Q05820, Q32KJ6, Q5I0D7, Q5MYT7, Q5U204, Q62662, Q63279, Q66H12, Q66HG4, Q6AYC4, Q6AYS4, Q6IFV1, Q78EG7, Q8CFC4, Q8CG45, Q8CJ11, Q99068, Q9EQV6, Q9ER31, Q9ET32, Q9R1T3, Q9R1T5, Q9Z339, A0A0G2JWD0, A0A0G2K1Q8, A0A140TAA1, B1H2A6, B2GUX7, B4F7C2, B5DEI2, B5DF79, D3Z9U8, D3ZCV0, D3ZJB2, D3ZJF9, D3ZR49, D3ZUC2, D3ZWR1, D4A2K1, D4A9Q5, D4AC85, F1LQI1, F1LRH4, M0R3V4, Q5RKH2 |
| cellular component | extracellular region part | 57 | F1LQ48, O70489, P04961, P17164, P20611, P23562, P24050, P24464, P25093, P30904, P45479, P50878, P62078, P70490, P70584, P97590, Q05820, Q32KJ6, Q5I0D7, Q5MYT7, Q5U204, Q62662, Q63279, Q66H12, Q66HG4, Q6AYC4, Q6AYS4, Q6IFV1, Q78EG7, Q8CG45, Q8CJ11, Q9EQV6, Q9ER31, Q9R1T3, Q9R1T5, Q9Z339, A0A0G2JWD0, A0A0G2K1Q8, A0A140TAA1, B1H2A6, B2GUX7, B4F7C2, B5DF79, D3Z9U8, D3ZCV0, D3ZJB2, D3ZJF9, D3ZR49, D3ZUC2, D3ZWR1, D4A2K1, D4A9Q5, D4AC85, F1LQI1, F1LRH4, M0R3V4, Q5RKH2 |
| cellular component | macromolecular complex | 68 | F1LQ48, O70597, P04961, P0C2C4, P0C588, P15205, P18211, P24050, P31503, P50878, P60825, P62078, Q4QQW4, Q4V7F5, Q5U204, Q5XIB2, Q5XIE0, Q62931, Q63279, Q64550, Q68FX7, Q6MGB6, Q8CHJ1, Q99M63, Q9EQN5, Q9JKW1, Q9R1B1, Q9Z1L0, A0A096MKF8, A0A0G2JV51, A3KNA0, A9CMA7, B0BNB5, B0BNI2, B1H2A6, B1WBY7, B1WBY8, B2GUX7, B2RYP8, B5DEL5, D3ZCR4, D3ZG54, D3ZGW2, D3ZJF7, D3ZU51, D3ZUC2, D3ZUL8, D3ZV30, D3ZV54, D3ZW27, D3ZY44, D4A3P1, D4A4J0, D4A604, D4A997, D4A9A3, D4AE02, F1LQC8, F1LRH4, F1LRQ6, F1LXT8, F1M365, G3V8C0, Q498C9, Q5EB90, Q5U1W6, Q5UT80, Q9ER28 |
| cellular component | membrane | 101 | D3ZHA0, D3ZHR2, F1LQ48, O70597, P07150, P0C588, P15205, P18211, P20611, P23562, P24050, P24464, P31214, P45479, P50878, P62078, P70490, P84039, P97612, Q3MHU5, Q4TU93, Q5FVG2, Q5HZA9, Q5MYT7, Q5U204, Q5XID7, Q62662, Q62931, Q63279, Q63619, Q64550, Q64566, Q68FU7, Q6EV70, Q6TEK3, Q78EG7, Q8CFC4, Q8CHJ1, Q8CJ11, Q99068, Q9EQN5, Q9ER31, Q9ET32, Q9JJ46, Q9JKW1, Q9R1B1, Q9WVK3, Q9Z122, Q9Z1L0, A0A096MKF8, A0A0G2JU12, A0A0G2JV51, A0A0G2JVH5, A0A0G2JWD0, A0A0G2JY11, A0A0G2K1Q8, A0A0G2K1Q9, A0A0G2K3D7, A0A0G2K4N5, A0A0G2KAP1, A0A140TAA1, A0A1W2Q6H4, A3KNA0, A9CMA7, B1H2A6, B1WBY7, B1WC35, B2RYP8, B5DF79, B5DFG9, D3ZBN3, D3ZCV0, D3ZD09, D3ZEA0, D3ZG54, D3ZGW2, D3ZJB2, D3ZKR8, D3ZNK1, D3ZQ77, D3ZR49, D3ZW27, D3ZZE3, D4A2N2, D4A3P1, D4A4S6, D4A604, D4A626, D4A9Q5, D4ACK7, F1LR42, F1LR52, F1LRH4, F1LY14, G3V757, G3V8C0, Q2TGK3, Q5RKH2, Q5U1W6, Q5UT80, Q9ER28 |
| cellular component | membrane part | 72 | D3ZHR2, O70597, P07150, P0C588, P18211, P20611, P23562, P24464, P31214, P45479, P70490, P84039, P97612, Q3MHU5, Q4TU93, Q5FVG2, Q5HZA9, Q5U204, Q5XID7, Q62662, Q62931, Q63279, Q63619, Q64550, Q64566, Q6EV70, Q6TEK3, Q78EG7, Q8CFC4, Q8CHJ1, Q8CJ11, Q9ER31, Q9ET32, Q9JJ46, Q9JKW1, Q9R1B1, Q9Z122, Q9Z1L0, A0A0G2JU12, A0A0G2JWD0, A0A0G2JY11, A0A0G2K1Q8, A0A0G2K4N5, A0A0G2KAP1, A0A140TAA1, A0A1W2Q6H4, B1WBY7, B1WC35, B5DF79, D3ZBN3, D3ZEA0, D3ZGW2, D3ZJB2, D3ZKR8, D3ZQ77, D3ZR49, D3ZW27, D3ZZE3, D4A2N2, D4A3P1, D4A4S6, D4A604, D4A626, D4A9Q5, D4ACK7, F1LR52, F1LY14, G3V757, Q2TGK3, Q5U1W6, Q5UT80, Q9ER28 |
| cellular component | membrane enclosed lumen | 61 | F1LQ48, P04961, P0C2C4, P16303, P20611, P24050, P30904, P31503, P50878, P60825, P62078, P70584, Q4QQW4, Q4V7F5, Q562C7, Q5HZE4, Q5I0H9, Q5MYT7, Q5XIE0, Q62662, Q64380, Q68FX7, Q6AYC4, Q6MGB6, Q99068, Q99M63, Q9R1B1, Q9Z1L0, A0A096MKF8, A0A0G2JU45, A0A0G2JV51, A0A0G2JVH5, A0A0G2K3D7, A3KNA0, A9CMA7, B0BNI2, B1WBY8, B2RYP8, B5DF79, D3ZCR4, D3ZD09, D3ZJF7, D3ZU51, D3ZUL8, D3ZV30, D3ZV54, D3ZY44, D4A3P1, D4A4J0, D4A997, D4A9A3, D4AC65, D4AE02, F1LQC8, F1LQI1, F1LR42, F1M365, G3V6P6, G3V8C0, Q498C9, Q5EB90 |
| cellular component | organelle | 166 | D3ZHA0, D3ZHR2, F1LQ48, O08701, O08836, O70489, O70597, O88588, P04961, P0C2C4, P11466, P15205, P16303, P17164\|, P20611, P23562, P24050, P24464, P25093, P30904, P31214, P31503, P32232, P45479, P50878, P60825, P62078, P70490, P70584, P97590, P97612, Q32KJ6, Q4QQW4, Q4V7F5, Q562C7, Q5FVG2, Q5HZA9, Q5HZE4, Q5I0D7, Q5I0H9, Q5MYT7, Q5U204, Q5U3Z3, Q5XI55, Q5XIB2, Q5XID7, Q5XIE0, Q62662, Q62931, Q63279, Q63525, Q63619, Q64380, Q64550, Q66H12, Q66HG4, Q68FU7, Q68FX7, Q6AYC4, Q6AYS4, Q6EV70, Q6IFV1, Q6MGB6, Q6TEK3, Q78EG7, Q8CFC4, Q8CG45, Q8CHJ1, Q8CJ11, Q99068, Q99M63, Q9EQN5, Q9EQV6, Q9ER31, Q9ET32, Q9JJ46, Q9JKW1, Q9QZI7, Q9R1B1, Q9R1T3, Q9R1T5, Q9WVK3, Q9Z122, Q9Z1L0, Q9Z339, A0A096MKF8, A0A0G2JU12, A0A0G2JU45, A0A0G2JV51, A0A0G2JVH5, A0A0G2JWD0, A0A0G2K1B6, A0A0G2K1Q8, A0A0G2K1Q9, A0A0G2K3D7, A0A0G2K402, A0A0G2K4N5, A0A0G2KAP1,; A0A140TAA1, A3KNA0, A9CMA7, B0BNB5, B0BNI2, B1H2A6, B1WBY7, B1WBY8, B2GUX7, B2RYP8, B4F7C2, B5DF79, D3Z9U8, D3ZCR4, D3ZCV0, D3ZD09, D3ZEA0, D3ZG54, D3ZGW2, D3ZJB2, D3ZJF7, D3ZJF9, D3ZKR8, D3ZMN2, D3ZNK1, D3ZPN5, D3ZR49, D3ZU51, D3ZUC2, D3ZUL8, D3ZUX7, D3ZV30, D3ZV54, D3ZW27, D3ZWR1, D3ZY44, D4A2K1, D4A2N2, D4A3P1, D4A4J0, D4A604, D4A997, D4A9A3, D4A9Q5, D4AA35, D4AC65, D4AC85, D4AE02, F1LQC8, F1LQI1, F1LR42, F1LR52, F1LRQ6, F1LXT8, F1M365, G3V6P6, G3V757, G3V8C0, M0R3V4, M0R9N6, Q2TGK3, Q498C9, Q4VBH2, Q5EB90, Q5RKH2, Q5U1W6, Q5UT80, Q9ER28 |
| cellular component | organelle part | 118 | D3ZHA0, D3ZHR2, F1LQ48, O08836, O70597, P04961, P0C2C4, P15205, P16303, P20611, P23562, P24050, P24464, P30904, P31214, P31503, P50878, P60825, P62078, P70584, P97612, Q4QQW4, Q4V7F5, Q562C7, Q5HZA9, Q5HZE4, Q5I0H9, Q5MYT7, Q5U204, Q5XIB2, Q5XID7, Q5XIE0, Q62662, Q62931, Q63279, Q63525, Q63619, Q64380, Q64550, Q68FU7, Q68FX7, Q6AYC4, Q6EV70, Q6IFV1, Q6MGB6, Q6TEK3, Q78EG7, Q8CFC4, Q8CG45, Q8CHJ1, Q99068, Q99M63, Q9ER31, Q9ET32, Q9JJ46, Q9JKW1, Q9R1B1, Q9WVK3, Q9Z122, Q9Z1L0, A0A096MKF8, A0A0G2JU12, A0A0G2JU45, A0A0G2JV51, A0A0G2JVH5, A0A0G2JWD0, A0A0G2K1Q8, A0A0G2K3D7, A0A0G2K4N5, A0A0G2KAP1, A3KNA0, A9CMA7, B0BNB5, B0BNI2, B1WBY7, B1WBY8, B2RYP8, B4F7C2, B5DF79, D3ZCR4, D3ZCV0, D3ZD09, D3ZEA0, D3ZG54, D3ZGW2, D3ZJF7, D3ZKR8, D3ZNK1, D3ZR49, D3ZU51, D3ZUL8, D3ZV30, D3ZV54, D3ZW27, D3ZY44, D4A2N2, D4A3P1, D4A4J0, D4A604, D4A997, D4A9A3, D4AC65, D4AE02, F1LQC8, F1LQI1, F1LR42, F1LR52, F1LRQ6, F1LXT8, F1M365, G3V6P6, G3V757, G3V8C0, Q2TGK3, Q498C9, Q5EB90, Q5U1W6, Q9ER28 |
| cellular component | supramolecular complex | 12 | D3ZHA0, O08836, P15205, P23562, P50878, Q63279, Q63525, Q6IFV1, B2RYP8, B4F7C2, D3ZCV0, Q498C9 |
| cellular component | synapse | 4 | P15205, P45479, A0A0G2K1Q9, A0A140TAA1 |
| cellular component | synapse part | 3 | P15205, P45479, A0A0G2K1Q9 |
| cellular component | virion | 1 | F1LQ48 |
| cellular component | virion part | 1 | F1LQ48 |
| molecular function | antioxidant activity | 2 | Q9Z339, A0A0G2JU12 |
| molecular function | binding | 135 | A1IGU3, D3ZHA0, D3ZHR2, F1LQ48, O08701, O08836, O70597, O88588, P04961, P07150, P0C2C4, P11466, P15205, P17164, P23562, P24050, P24464, P25093, P30904, P31214, P31503, P32232, P50878, P60825, P62078, P70490, P70584, P84039, P97590, P97612, Q32KJ6, Q4QQW4, Q4TU93, Q4V7F5, Q562C7, Q5FVG2, Q5HZE4, Q5I0D7, Q5MYT7, Q5U204, Q5XI55, Q5XIE0, Q62662, Q62931, Q63279, Q63525, Q63619, Q64380, Q64550, Q64566, Q66H12, Q66HG4, Q68FU7, Q68FX7, Q6AYC4, Q6IFV1, Q6MGB6, Q6TEK3, Q8CFC4, Q8CHJ1, Q99068, Q9EQN5,Q9EQV6, Q9ET32, Q9JKW1, Q9QZI7, Q9R1B1, Q9R1T5, Q9WVK3, Q9Z1L0, A0A096MKF1, A0A096MKF8, A0A0G2JV51, A0A0G2JVH5, A0A0G2JWD0, A0A0G2K1B6, A0A0G2K1Q8, A0A0G2K1Q9, A0A0G2K2P4, A0A0G2K3D7, A0A0G2K402, A0A0G2K4N5, A0A0G2K8M7, A0A1W2Q6H4, A3KNA0, A9CMA7, B1H2A6, B1WBY7, B1WBY8, B2GUX7, B2RYP8, B4F7C2, B5DF79, B5DFG9, D3Z9U8, D3ZBN3, D3ZCV0, D3ZEA0, D3ZG54, D3ZJF7, D3ZJF9, D3ZMN2, D3ZPN5, D3ZR49, D3ZU51, D3ZUC2, D3ZUL8, D3ZUX7, D3ZV30, D3ZV54, D3ZW27, D3ZWR1, D4A2K1, D4A2N2, D4A3P1, D4A4J0, D4A626, D4A997, D4A9Q5, D4AC85, D4AE02, F1LQC8, F1LQI1, F1LR42, F1LR52, F1LRH4, F1LXT8, G3V6P6, M0R9N6, Q498C9, Q4VBH2, Q5EB90, Q5RKH2, Q5UT80, Q9ER28 |
| molecular function | catalytic activity | 103 | D3ZHR2, O08701, O70489, P04961, P07150, P11466, P16303, P17164, P20611, P24464, P25093, P30904, P31214, P32232, P45479, P70584, P84039, P97612, Q05820, Q32KJ6, Q4QQW4, Q5HZE4, Q5I0D7, Q5I0H9, Q5MYT7, Q5U3Z3, Q5XI55, Q5XIB2, Q62662, Q63619, Q64380, Q64550, Q64566, Q66H12, Q66HG4, Q68FU7, Q6AYS4, Q6EV70, Q6MGB6, Q6TEK3, Q78EG7, Q8CFC4, Q8CG45, Q8CHJ1, Q9EQN5, Q9EQV6, Q9ER31, Q9ET32, Q9JJ46, Q9R1T3, Q9R1T5, Q9WVK3, Q9Z122, Q9Z1L0, Q9Z339, A0A096MKF1, A0A096MKF8, A0A0G2JU12, A0A0G2JU45, A0A0G2JV51, A0A0G2JVH5, A0A0G2K1Q8, A0A0G2K2P4, A0A0G2K4N5, A0A0G2KAP1, A0A1W2Q6H4, B1WBY8, B2RYP8, B4F7C2, B5DEI2, B5DEL5, B5DF79,B5DFG9, D3ZBN3, D3ZG54, D3ZJF7, D3ZJF9, D3ZPN5, D3ZR49, D3ZU51, D3ZUC2, D3ZUX7, D3ZV30, D3ZW27, D3ZWR1, D4A2K1, D4A2N2, D4A9A3, D4A9Q5, D4AA35, D4AC85, D4AE02, F1LQC8, F1LQI1, F1LR52, F1LXT8, G3V757, M0R9N6, Q2TGK3, Q4VBH2, Q5EB90, Q5RKH2, Q9ER28 |
| molecular function | molecular function regulator | 12 | A1IGU3, O08836, P04961, P30904, Q5U204, Q5XIE0, Q64550, Q6MGB6, Q99068, A0A0G2JU12, A9CMA7, D4A2N2 |
| molecular function | molecular transducer activity | 6 | Q4TU93, Q8CJ11, A0A096MKF8, D3ZBN3, D4A4S6, F1LQC8 |
| molecular function | signal transducer activity | 5 | Q4TU93, Q8CJ11, D3ZBN3, D3ZW27, D4A4S6 |
| molecular function | structural molecule activity | 11 | P0C2C4, P24050, P50878, Q5FVG2, Q5U204, Q63279, Q6IFV1, A0A0G2K1Q9, B2RYP8, B4F7C2, D3ZY44 |
| molecular function | transcription regulator activity | 9 | P31503, Q4QQW4, Q9EQN5, A0A096MKF8, A0A0G2JVH5, A0A0G2K1B6, A0A0G2K402, D3ZCV0, Q498C9 |
| molecular function | translation regulator activity | 1 | P60825 |
| molecular function | transporter activity | 12 | D3ZHR2, P0C588, P23562, P62078, Q64566, Q9JKW1, A0A0G2K1Q8, D3ZGW2, F1LR42, F1LR52, F1LY14, Q9ER28 |
