# Supplementary material for: Mitogen-activated protein kinase pathway and four genes involved in the development of benign prostatic hyperplasia: in vivo and vitro validation
Source: Front Immunol. 2025 Nov 11;16:1606607. doi: 10.3389/fimmu.2025.1606607 (PMC12644057; doi:10.3389/fimmu.2025.1606607)
Supplement: Supplementary file 8 [file Table6.docx]

| **Supplementary Table 6. KOG function analysis of differentially proteins in BPH and sham rats.** | | | | |  |
| --- | --- | --- | --- | --- | --- |
| **Code** | **Functional-categories** | **Family** | **Number of EDPs** | **Protein members** | |
| A | RNA processing and modification | INFORMATION STORAGE AND PROCESSING | 12 | P50878, P60825, A0A0G2JU45,A3KNA0,F1M365,A0A0G2K402,Q9EQN5,B0BNB5,B5DF79,D3ZUC2,D3ZV54,F1LQ48 | |
| B | Chromatin structure and dynamics | INFORMATION STORAGE AND PROCESSING | 4 | Q4QQW4, Q5BKC6, B0BNB5, B1WBY8 | |
| C | Energy production and conversion | METABOLISM | 5 | Q9Z122, Q68FU7, Q64550, D3ZD09,Q8CG45 | |
| D | Cell cycle control, cell division, chromosome partitioning | CELLULAR PROCESSES AND SIGNALING | 10 | D3ZPN5,F1LQC8,Q63279,D4AA35,Q6IFV1,B0BNB5,A9CMA7,F1LRQ6,Q5XIE0,F1LY14 | |
| E | Amino acid transport and metabolism | METABOLISM | 7 | D4A4J0,Q64380,G3V757,O08701,P70584,Q5I0D7,P32232 | |
| F | Nucleotide transport and metabolism | METABOLISM | 1 | Q9ER31 | |
| G | Carbohydrate transport and metabolism | METABOLISM | 11 | Q66H12,Q5RKH2,Q64550,D3ZR49,Q6AYS4,Q66HG4,G3V757,P25093,D3ZJF9,P17164,D3ZCR4 | |
| H | Coenzyme transport and metabolism | METABOLISM | 1 | Q68FU7 | |
| I | Lipid transport and metabolism | METABOLISM | 16 | A0A1W2Q6H4,Q9Z122,P24464,D3ZUX7,Q99068,P45479,D3ZHR2,P20611,P97612,P70584,P11466,A0A0G2K1Q8,O70489,Q9JJ46,P31214,F1LR42 | |
| J | Translation, ribosomal structure and biogenesis | INFORMATION STORAGE AND PROCESSING | 9 | Q4VBH2,D3ZY44,P97612,A0A0G2K402,P0C2C4,Q562C7,P24050,Q5HZE4,D3ZU51 | |
| K | Transcription | INFORMATION STORAGE AND PROCESSING | 10 | Q5EB90,F1LQC8,P31503,A0A0G2K1B6,A0A096MKF8,A0A0G2JVH5,D4A997,B2GUX7,D3ZV54,D3ZV30 | |
| L | Replication, recombination and repair | INFORMATION STORAGE AND PROCESSING | 5 | F1LQC8,A3KNA0,A0A0G2JVH5,Q9EQN5,P04961 | |
| M | Cell wall/membrane/envelope biogenesis | CELLULAR PROCESSES AND SIGNALING | 3 | D4A604,D3ZNK1,D4AC65 | |
| O | Posttranslational modification, protein turnover, chaperones | CELLULAR PROCESSES AND SIGNALING | 18 | Q99068,D4AC85,P45479,Q5I0H9,D3ZG54,Q5XIB2,D4A3P1,Q9R1T3,D4A604,Q6MGB6,Q6EV70,Q9Z339,A0A0G2KAP1,F1LRQ6,O70489,Q6TEK3,Q5XI55,D4AC65 | |
| P | Inorganic ion transport and metabolism | METABOLISM | 3 | P23562,Q64566,Q9ER28 | |
| Q | Secondary metabolites biosynthesis, transport and catabolism | METABOLISM | 8 | A0A1W2Q6H4,P24464,D3ZG54,Q9ER28,A0A0G2K2P4,F1LR52,A0A0G2K4N5,B5DEI2 | |
| R | General function prediction only | POORLY CHARACTERIZED | 41 | B5DEL5,Q9WVK3,Q63619,A0A0G2JWD0,A0A0G2K1Q9,P60825,Q32KJ6,F1LQC8,A0A0G2K8M7,Q63279,A0A0G2JU45,G3V6P6,Q6IFV1,D3ZHR2,Q2TGK3,Q99M63,D4A3P1,D4A9Q5,Q9R1T3,B1WBY7,F1LQI1,D3ZEL0,Q5U3Z3,A0A0G2K402,P84039,Q498C9,A0A0G2K1Q8,Q5FVG2,P70490,D4A4S6,A0A0G2JV51,D3ZV54,Q9QZI7,B0BNI2,Q5I0D7,Q5XIE0,P16303,A0A0G2JY11,A0A0G2K3D7,Q8CHJ1,F1LR42 | |
| S | Function unknown | POORLY CHARACTERIZED | 16 | D4A9A3,B1WC35,Q4V7F5,D3ZKR8,Q6MGB6,D4ACK7,Q5U1W6,Q68FX7,D3ZMN2,P0C588\|,D3ZUL8,D3ZZE3,D4AE02,B0BNI2,F1LY14,D4AC65 | |
| T | Signal transduction mechanisms | CELLULAR PROCESSES AND SIGNALING | 22 | B5DEL5,A0A0G2K1Q9,Q62662,Q4TU93,A1IGU3,Q99068,D3ZBN3,O08836,D3ZEL0,P97612,Q5BKC6,D3ZEA0,D3ZML4,P70490,Q8CJ11,D3ZW27,Q9Z1L0,Q78EG7,P16303,Q63525,D4AC65,F1LR42 | |
| U | Intracellular trafficking, secretion, and vesicular transport | CELLULAR PROCESSES AND SIGNALING | 15 | Q99068,Q63279,D3ZGW2,Q6IFV1,O88588,P07150,Q9JKW1,O70597,D4A2N2,Q62931,P62078,A0A0G2KAP1,D3ZNK1,Q9R1B1,F1LR42 | |
| V | Defense mechanisms | CELLULAR PROCESSES AND SIGNALING | 5 | Q4TU93,Q99068,30904,P70490,Q78EG7 | |
| W | Extracellular structures | CELLULAR PROCESSES AND SIGNALING | 3 | F1LRH4,P97590,P70490 | |
| Y | Nuclear structure | CELLULAR PROCESSES AND SIGNALING | 3 | Q63279,Q6IFV1,B0BNB5 | |
| Z | Cytoskeleton | CELLULAR PROCESSES AND SIGNALING | 12 | P15205,Q6AYC4,A0A096MKF1,D4A626,D3ZCV0,F1LXT8,G3V8C0,D3ZEA0,B4F7C2,D3ZHA0,B2RYP8,D3ZCG2 | |
