# Supplementary material for: Mitogen-activated protein kinase pathway and four genes involved in the development of benign prostatic hyperplasia: in vivo and vitro validation
Source: Front Immunol. 2025 Nov 11;16:1606607. doi: 10.3389/fimmu.2025.1606607 (PMC12644057; doi:10.3389/fimmu.2025.1606607)
Supplement: Supplementary file 9 [file Table7.docx]

| **Supplementary Table 7. KEGG pathway analysis of differentially proteins in BPH and sham rats.** | | | | |
| --- | --- | --- | --- | --- |
| **Pathway** | **Gene name** | **Level 1** | **Level 2** | **P value** |
| Glycosphingolipid biosynthesis - lacto and neolacto series | Q8CFC4, Q9ET32, G3V757 | Metabolism | Glycan biosynthesis and metabolism | 0.00106005 |
| Ubiquinone and other terpenoid-quinone biosynthesis | Q63619, Q68FU7, Q6TEK3 | Metabolism | Metabolism of cofactors and vitamins | 0.00106005 |
| Lysosome | O70489, P20611, P45479, Q32KJ6, Q66H12, Q9EQV6, Q9R1T3, D3ZGW2, D3ZJF9 | Cellular Processes | Transport and catabolism | 0.003562315 |
| Glycosphingolipid biosynthesis - globo and isoglobo series | Q66H12, D3ZJF9 | Metabolism | Glycan biosynthesis and metabolism | 0.020334 |
| ABC transporters | D3ZHR2, A0A0G2K1Q8, F1LR52 | Environmental information Processing | Membrane transport | 0.02227426 |
| Cell cycle | P04961, Q4QQW4, B1WBY8, F1LQC8, F1LRQ6 | Cellular Processes | Cell growth and death | 0.02228306 |
| Metabolism of xenobiotics by cytochrome P450 | Q64550, Q8CG45, Q9Z339, A0A0G2JU12 | Metabolism | Xenobiotics biodegradation and metabolism | 0.03062166 |
| Steroid hormone biosynthesis | P31214, Q64550, A0A0G2K4N5 | Metabolism | Lipid metabolism | 0.03066674 |
| Inflammatory bowel disease (IBD) | P18211, Q5UT80 | Human Diseases | Immune diseases | 0.03606191 |
| Metabolic pathways | O08701, O70489, P16303, P20611, P24464, P25093, P32232, P45479, P70584, Q32KJ6, Q5HZE4, Q63525, Q63619, Q64380, Q64550, Q66H12, Q66HG4, Q68FU7, Q6TEK3, Q8CFC4, Q8CHJ1, Q9ET32, Q9JJ46, Q9R1T5, Q9Z1L0, A0A1W2Q6H4, B1WBY7, B5DEI2, D3ZD09, D3ZJF9, D3ZR49, D3ZWR1, D4A2K1, D4A2N2, D4A604, G3V757, Q5RKH2 | Metabolism | Global and overview maps | 0.04023972 |
| Galactose metabolism | Q66HG4, D3ZJF9, Q5RKH2 | Metabolism | Carbohydrate metabolism | 0.04048994 |
| Longevity regulating pathway - multiple species | Q4QQW4, Q9Z1L0, B1WBY8, B1WC35 | Organismal Systems | Aging | 0.04523349 |
