# Supplementary material for: Mitogen-activated protein kinase pathway and four genes involved in the development of benign prostatic hyperplasia: in vivo and vitro validation
Source: Front Immunol. 2025 Nov 11;16:1606607. doi: 10.3389/fimmu.2025.1606607 (PMC12644057; doi:10.3389/fimmu.2025.1606607)
Supplement: Supplementary file 10 [file Table8.docx]

| **Supplementary Table 8. The subcellular localization of identified DEPs in comparison group.** | | | |
| --- | --- | --- | --- |
| **Seq ID** | **Subcellular location** | **Description** | **Gene name** |
| A1IGU3 | nucl | Rho guanine nucleotide exchange factor 37 | Arhgef37 |
| D3ZHA0 | cyto | Filamin-C | Flnc |
| D3ZHR2 | plas | ATP-binding cassette sub-family D member 1 | Abcd1 |
| F1LQ48 | nucl | Heterogeneous nuclear ribonucleoprotein L | Hnrnpl |
| O08701 | mito | Arginase-2, mitochondrial | Arg2 |
| O08836 | cyto | Immunoglobulin-binding protein 1 | Igbp1 |
| O70489 | extr | Lysosomal thioesterase PPT2 | Ppt2 |
| O70597 | mito | Peroxisomal membrane protein 11A | Pex11a |
| O88588 | nucl | Phosphofurin acidic cluster sorting protein 1 | Pacs1 |
| P04961 | cyto_nucl | Proliferating cell nuclear antigen | Pcna |
| P07150 | cyto | Annexin A1 OS=Rattus norvegicus | Anxa1 |
| P0C2C4 | cyto | 39S ribosomal protein L10, mitochondrial | Mrpl10 |
| P0C588 | plas | Metal transporter CNNM4 | Cnnm4 |
| P11466 | cyto | Peroxisomal carnitine O-octanoyltransferase | Crot |
| P15205 | nucl | Microtubule-associated protein 1B | Map1b |
| P16303 | extr | Carboxylesterase 1D | Ces1d |
| P17164 | extr | Tissue alpha-L-fucosidase | Fuca1 |
| P18211 | extr | Rano class II histocompatibility antigen, D-1 beta chain | RT1-Db |
| P20611 | extr | Lysosomal acid phosphatase | Acp2 |
| P23562 | plas | Band 3 anion transport protein | Slc4a1 |
| P24050 | cyto | 40S ribosomal protein S5 | Rps5 |
| P24464 | E.R. | Cytochrome P450 4A12 | Cyp4a12 |
| P25093 | cyto_nucl | Fumarylacetoacetase | Fah |
| P30904 | cyto_nucl | Macrophage migration inhibitory factor | Mif |
| P31214 | extr | 3-oxo-5-alpha-steroid 4-dehydrogenase 2 | Srd5a2 |
| P31503 | nucl | POU domain, class 2, transcription factor 1 (Fragment) | Pou2f1 |
| P32232 | cyto | Cystathionine beta-synthase | Cbs |
| P45479 | extr | Palmitoyl-protein thioesterase 1 | Ppt1 |
| P50878 | cyto | 60S ribosomal protein L4 | Rpl4 |
| P60825 | nucl | Cold-inducible RNA-binding protein | Cirbp |
| P62078 | extr | Mitochondrial import inner membrane translocase subunit Tim8 B | Timm8b |
| P70490 | extr | Lactadherin | Mfge8 |
| P70584 | mito | Short/branched chain specific acyl-CoA dehydrogenase, mitochondrial | Acadsb |
| P84039 | plas | Ectonucleotide pyrophosphatase/phosphodiesterase family member 5 | Enpp5 |
| P97590 | mito | Galectin-7 | Lgals7 |
| P97612 | extr | Fatty-acid amide hydrolase 1 | Faah |
| Q05820 | extr | Putative lysozyme C-2 | Lyz2 |
| Q32KJ6 | extr | N-acetylgalactosamine-6-sulfatase | Galns |
| Q3MHU5 | plas | Reticulophagy regulator 2 | Retreg2 |
| Q4QQW4 | cyto | Histone deacetylase 1 | Hdac1 |
| Q4TU93 | pero | C-type mannose receptor 2 | Mrc2 |
| Q4V7F5 | nucl | PIH1 domain-containing protein 1 | Pih1d1 |
| Q562C7 | nucl | Pumilio homolog 3 | Pum3 |
| Q5BKC6 | cyto | HSPB1-associated protein 1 | Hspbap1 |
| Q5FVG2 | nucl | Band 4.1-like protein 5 | Epb41l5 |
| sp\|Q5HZA9 | cyto | Transmembrane protein 126A | Tmem126a |
| Q5HZE4 | plas | Methylthioribose-1-phosphate isomerase | Mri1 |
| Q5I0D7 | mito | Xaa-Pro dipeptidase | Pepd |
| Q5I0H9 | extr | Protein disulfide-isomerase A5 | Pdia5 |
| Q5MYT7 | cyto | 2'-5'-oligoadenylate synthase 3 | Oas3 |
| Q5U204 | cyto | Ragulator complex protein LAMTOR3 | Lamtor3 |
| Q5U3Z3 | cyto | Isochorismatase domain-containing protein 2 | Isoc2 |
| Q5XI55 | cyto | Peptide-N(4)-(N-acetyl-beta-glucosaminyl)asparagine amidase | Ngly1 |
| Q5XIB2 | nucl | Spliceosome-associated protein CWC27 homolog | Cwc27 |
| Q5XID7 | mito | Armadillo repeat-containing X-linked protein 3 | Armcx3 |
| Q5XIE0 | cyto | Acidic leucine-rich nuclear phosphoprotein 32 family member E | Anp32e |
| Q62662 | mito | Tyrosine-protein kinase FRK | Frk |
| Q62931 | cyto | Golgi SNAP receptor complex member 1 | Gosr1 |
| Q63279 | mito | Keratin, type I cytoskeletal 19 | Krt19 |
| Q63525 | cyto | Nuclear migration protein nudC | Nudc |
| Q63619 | cyto | 5-demethoxyubiquinone hydroxylase, mitochondrial (Fragment) | Coq7 |
| Q64380 | mito | Sarcosine dehydrogenase, mitochondrial | Sardh |
| Q64550 | E.R. | UDP-glucuronosyltransferase 1-1 | Ugt1a1 |
| Q64566 | plas | Calcium-transporting ATPase type 2C member 1 | Atp2c1 |
| Q66H12 | extr | Alpha-N-acetylgalactosaminidase | Naga |
| Q66HG4 | cyto | Aldose 1-epimerase | Galm |
| Q68FU7 | mito | Ubiquinone biosynthesis monooxygenase COQ6, mitochondrial | Coq6 |
| Q68FX7 | nucl | THO complex subunit 5 homolog | Thoc5 |
| Q6AYC4 | cyto_nucl | Macrophage-capping protein | Capg |
| Q6AYS4 | extr | Plasma alpha-L-fucosidase | Fuca2 |
| Q6EV70 | extr | GDP-fucose protein O-fucosyltransferase 1 | Pofut1 |
| Q6IFV1 | nucl | Keratin, type I cytoskeletal 14 | Krt14 |
| Q6MGB6 | nucl | E3 ubiquitin-protein ligase RING1 | Ring1 |
| Q6TEK3 | plas | Vitamin K epoxide reductase complex subunit 1-like protein 1 | Vkorc1l1 |
| Q78EG7 | cyto | Protein tyrosine phosphatase type IVA 1 | Ptp4a1 |
| Q8CFC4 | extr | Histo-blood group ABO system transferase 2 | Abo2 |
| Q8CG45 | mito | Aflatoxin B1 aldehyde reductase member 2 | Akr7a2 |
| Q8CHJ1 | plas | Phosphatidylinositol glycan anchor biosynthesis class U protein | Pigu |
| Q8CJ11 | plas | Adhesion G-protein coupled receptor G2 | Adgrg2 |
| Q99068 | extr | Alpha-2-macroglobulin receptor-associated protein | Lrpap1 |
| Q99M63 | cyto | WD40 repeat-containing protein SMU1 | Smu1 |
| Q9EQN5 | nucl | DNA-binding protein SMUBP-2 | Ighmbp2 |
| Q9EQV6 | extr | Tripeptidyl-peptidase 1 | Tpp1 |
| Q9ER31 | extr | Ectonucleoside triphosphate diphosphohydrolase 6 | Entpd6 |
| Q9ET32 | E.R. | Histo-blood group ABO system transferase 1 | Abo |
| Q9JJ46 | plas | 3-beta-hydroxysteroid-Delta(8),Delta(7)-isomerase | Ebp |
| Q9JKW1 | cyto | Mitochondrial import inner membrane translocase subunit Tim22 | Timm22 |
| Q9QZI7 | nucl | tRNA selenocysteine 1-associated protein 1 | Trnau1ap |
| Q9R1B1 | extr | Mitochondrial import inner membrane translocase subunit Tim10 B | Timm10b |
| Q9R1T3 | extr | Cathepsin Z | Ctsz |
| Q9R1T5 | cyto | Aspartoacylase | Aspa |
| Q9WVK3 | cyto | Peroxisomal trans-2-enoyl-CoA reductase | Pecr |
| Q9Z122 | plas | Acyl-CoA 6-desaturase | Fads2 |
| Q9Z1L0 | cyto | Phosphatidylinositol 4,5-bisphosphate 3-kinase catalytic subunit beta isoform | Pik3cb |
| Q9Z339 | cyto | Glutathione S-transferase omega-1 | Gsto1 |
| A0A096MKF1 | cyto | Inositol hexakisphosphate and diphosphoinositol-pentakisphosphate kinase (Fragment) | Ppip5k2 |
| A0A096MKF8 | plas | Mediator complex subunit 12 | Med12 |
| A0A0G2JU12 | mito | Microsomal glutathione S-transferase 2 | Mgst2 |
| A0A0G2JU45 | mito | tRNA (guanine(37)-N1)-methyltransferase | Trmt5 |
| A0A0G2JV51 | nucl | RNA cytidine acetyltransferase | Nat10 |
| A0A0G2JVH5 | nucl | Helicase-like transcription factor | Hltf |
| A0A0G2JWD0 | plas | Prominin 1 | Prom1 |
| tr\|A0A0G2JY11 | plas | Tetraspanin | Tspan9 |
| A0A0G2K1B6 | nucl | Nuclear factor 1 | Nf1x |
| A0A0G2K1Q8 | plas | ATP-binding cassette subfamily A member 3 | Abca3 |
| A0A0G2K1Q9 | nucl | Erythrocyte membrane protein band 4.1-like 3 | Epb41l3 |
| A0A0G2K2P4 | E.R. | Cytochrome P450, family 2, subfamily t, polypeptide 1 | Cyp2t1 |
| A0A0G2K3D7 | nucl | Zinc finger and BTB domain-containing 1 | Zbtb1 |
| tr\|A0A0G2K402 | nucl | Myelin expression factor 2 | Myef2 |
| A0A0G2K4N5 | mito | 25-hydroxycholesterol 7-alpha-hydroxylase | Cyp7b1 |
| A0A0G2K8M7 | nucl | TPD52-like 1 | Tpd52l1 |
| A0A0G2KAP1 | extr | Endoplasmic reticulum oxidoreductase 1 beta | Ero1b |
| A0A140TAA1 | extr | Immunoglobulin superfamily, member 8 | Igsf8 |
| A0A1W2Q6H4 | cyto_nucl | Cytochrome P450, family 4, subfamily f, polypeptide 17 (Fragment) | Cyp4f17 |
| A3KNA0 | cyto | RNA helicase aquarius | Aqr |
| A9CMA7 | nucl | Cyclin T2 | Ccnt2 |
| B0BNB5 | nucl | LOC683983 protein | Nup43 |
| B0BNI2 | nucl | RCG47471, isoform CRA_a | Wdr74 |
| B1H2A6 | nucl | FMR1 autosomal homolog 2 | Fxr2 |
| B1WBY7 | E.R. | ER lipid raft-associated 1 | Erlin1 |
| B1WBY8 | nucl | Hdac2 protein (Fragment) | Hdac2 |
| B1WC35 | plas | Transmembrane protein 161A | Tmem161a |
| B2GUX7 | extr | Cellular repressor of E1A-stimulated genes (Predicted), isoform CRA_b | Creg1 |
| B2RYP8 | cyto | Gamma-tubulin complex component | Tubgcp2 |
| B4F7C2 | cyto_nucl | Tubulin beta chain | Tubb4a |
| B5DEI2 | plas | Amine oxidase | Lao1 |
| B5DEL5 | cyto | Kelch-like 9 (Drosophila) | Klhl9 |
| B5DF79 | extr | Rnaset2 protein | Rnaset2 |
| B5DFG9 | plas | Pik3ap1 protein | Pik3ap1 |
| D3Z9U8 | cyto | S100 calcium-binding protein A7-like 2 | S100a7l2 |
| D3ZBN3 | plas | Eph receptor A2 | Epha2 |
| D3ZCG2 | nucl | Kinesin family member 21A | Kif21a |
| D3ZCR4 | nucl | Protein phosphatase 4, regulatory subunit 3B | Ppp4r3b |
| D3ZCV0 | cyto_nucl | Actinin alpha 2 | Actn2 |
| D3ZD09 | extr | Cytochrome c oxidase subunit | Cox6b1 |
| D3ZEA0 | plas | Fibronectin type III domain containing 3a (Predicted), isoform CRA_a | Fndc3a |
| D3ZEL0 | nucl | Uncharacterized protein | LOC500684 |
| D3ZG54 | nucl | Uncharacterized protein |  |
| D3ZGW2 | nucl | AP-1 complex subunit gamma | Ap1g2 |
| D3ZJB2 | extr | Glycoprotein integral membrane 1 | Ginm1 |
| D3ZJF7 | nucl | Telomeric repeat-binding factor | Terf2 |
| D3ZJF9 | extr | Alpha-galactosidase | Gla |
| D3ZKR8 | extr | Protein kish | Tmem167a |
| D3ZML4 | cyto | Similar to RIKEN cDNA 5730502D15 gene (Predicted) | Trabd |
| D3ZMN2 | nucl | Histone PARylation factor 1 | Hpf1 |
| D3ZNK1 | cyto | Metaxin 3 | Mtx3 |
| D3ZPN5 | nucl | Mitochondrial poly(A) polymerase | Mtpap |
| D3ZQ77 | plas | Similar to HTGN29 protein; keratinocytes associated transmembrane protein 2, isoform CRA_a | RGD1310352 |
| D3ZR49 | mito | alpha-1,2-Mannosidase | Man1a2 |
| D3ZU51 | cyto | Ribonuclease P/MRP subunit p30 | Rpp30 |
| D3ZUC2 | nucl | Mov10 RISC complex RNA helicase | Mov10 |
| D3ZUL8 | nucl | Zinc finger CCHC-type-containing 8 | Zcchc8 |
| D3ZUX7 | mito | Acyl-CoA synthetase family member 3 | Acsf3 |
| D3ZV30 | cyto | DNA-directed RNA polymerase subunit beta | Polr3b |
| D3ZV54 | mito | PWP2, small subunit processome component | Pwp2 |
| D3ZW27 | cyto | Mitogen-activated protein kinase kinase kinase 5 | Map3k5 |
| D3ZWR1 | extr | 5', 3'-nucleotidase, cytosolic | Nt5c |
| D3ZY44 | cyto | Mitochondrial ribosomal protein S2 | Mrps2 |
| D3ZZE3 | plas | Armadillo-like helical domain-containing 3 | Armh3 |
| D4A2K1 | mito | 4-hydroxy-2-oxoglutarate aldolase 1 | Hoga1 |
| D4A2N2 | cyto | Inositol polyphosphate-5-phosphatase B | Inpp5b |
| D4A3P1 | nucl | Ubiquilin 4 | Ubqln4 |
| D4A4J0 | nucl | SPT16 homolog, facilitates chromatin-remodeling subunit | Supt16h |
| D4A4S6 | plas | Olfactory receptor 1507 | Olr1507 |
| D4A604 | plas | Phosphatidylinositol glycan anchor biosynthesis, class T | Pigt |
| D4A626 | plas | Calmin | Clmn |
| D4A997 | nucl | HIV TAT specific factor 1 (Predicted) | Htatsf1 |
| D4A9A3 | nucl | Centromere protein V | Cenpv |
| tr\|D4A9Q5 | extr | Carboxypeptidase M | Cpm |
| D4AA35 | mito | Acetylserotonin O-methyltransferase-like | Asmtl |
| D4AC65 | extr | Cytochrome c oxidase assembly factor 7 | Coa7 |
| D4AC85 | extr | Glutaminyl-peptide cyclotransferase | Qpct |
| D4ACK7 | plas | Cyclin and CBS domain divalent metal cation transport mediator 3 | Cnnm3 |
| D4AE02 | nucl | Uncharacterized protein |  |
| F1LQC8 | cyto | Cyclin-dependent kinase 7 | Cdk7 |
| F1LQI1 | mito | Hydroxyacyl glutathione hydrolase | Hagh |
| F1LR42 | cyto | RUN and FYVE domain-containing 1 | Rufy1 |
| F1LR52 | plas | ATP-binding cassette subfamily C member 4 | Abcc4 |
| F1LRH4 | extr | Laminin subunit gamma 2 | Lamc2 |
| F1LRQ6 | extr | CDC23 (Cell division cycle 23, yeast, homolog), isoform CRA_b | Cdc23 |
| F1LXT8 | cyto | Dynein, axonemal, heavy chain 6 | Dnah6 |
| F1LY14 | plas | Anoctamin | Ano9 |
| F1M365 | nucl | Integrator complex subunit 9-like | Ints9 |
| G3V6P6 | nucl | RNA binding motif protein 3, isoform CRA_a | Rbm3 |
| G3V757 | plas | Alpha-(1,3)-fucosyltransferase 4 | Fut4 |
| G3V8C0 | cyto | Dynactin subunit 5 | Dctn5 |
| M0R3V4 | extr | Myeloid-derived growth factor | Mydgf |
| M0R9N6 | cyto | 2 ' -5 ' oligoadenylate synthetase 1K | Oas1k |
| Q2TGK3 | plas | Palmitoyltransferase | Zdhhc3 |
| Q498C9 | nucl | RCG33491, isoform CRA_b | Zfp207 |
| Q4VBH2 | mito | tRNA nucleotidyl transferase 1 | Trnt1 |
| Q5EB90 | cysk | Polymerase (RNA) II (DNA directed) polypeptide C, 33kDa | Polr2c |
| Q5RKH2 | nucl | Galactokinase 1 | Galk1 |
| Q5U1W6 | cyto | MICOS complex subunit | Apool |
| Q5UT80 | plas | MHC class II antigen | BA1 |
| Q9ER28 | plas | Endothelial type gp91-phox | Cybb |
