# Supplementary material for: Mitogen-activated protein kinase pathway and four genes involved in the development of benign prostatic hyperplasia: in vivo and vitro validation
Source: Front Immunol. 2025 Nov 11;16:1606607. doi: 10.3389/fimmu.2025.1606607 (PMC12644057; doi:10.3389/fimmu.2025.1606607)
Supplement: Supplementary file 11 [file Table9.docx]

| **Supplementary Table 9. Network interaction analysis of protein-protein relationships of identified DEPs.** | | | | |
| --- | --- | --- | --- | --- |
| **Protein 1** | **Protein 2** | **Protein_cluster1** | **Protein_cluster2** | **Score** |
| Q5MYT7\|OAS3 | D3ZR49\|D3ZR49 | 10090.ENSMUSP00000035588 | 10090.ENSMUSP00000008907 | 165 |
| Q5MYT7\|OAS3 | Q5I0H9\|PDIA5 | 10090.ENSMUSP00000035588 | 10090.ENSMUSP00000023550 | 374 |
| Q5MYT7\|OAS3 | Q9QZI7\|TSAP1 | 10090.ENSMUSP00000035588 | 10090.ENSMUSP00000030730 | 161 |
| D4AC85\|D4AC85 | Q5MYT7\|OAS3 | 10090.ENSMUSP00000038732 | 10090.ENSMUSP00000035588 | 165 |
| B0BNI2\|B0BNI2 | Q9QZI7\|TSAP1 | 10090.ENSMUSP00000043315 | 10090.ENSMUSP00000030730 | 227 |
| B0BNI2\|B0BNI2 | Q5MYT7\|OAS3 | 10090.ENSMUSP00000043315 | 10090.ENSMUSP00000035588 | 222 |
| A0A0G2K1Q8\|A0A0G2K1Q8 | Q5I0H9\|PDIA5 | 10090.ENSMUSP00000045285 | 10090.ENSMUSP00000023550 | 169 |
| B0BNB5\|B0BNB5 | Q5MYT7\|OAS3 | 10090.ENSMUSP00000046732 | 10090.ENSMUSP00000035588 | 165 |
| B0BNB5\|B0BNB5 | B0BNI2\|B0BNI2 | 10090.ENSMUSP00000046732 | 10090.ENSMUSP00000043315 | 313 |
| A0A0G2KAP1\|A0A0G2KAP1 | Q5I0H9\|PDIA5 | 10090.ENSMUSP00000071864 | 10090.ENSMUSP00000023550 | 750 |
| A0A0G2KAP1\|A0A0G2KAP1 | Q5MYT7\|OAS3 | 10090.ENSMUSP00000071864 | 10090.ENSMUSP00000035588 | 433 |
| A0A0G2JWD0\|A0A0G2JWD0 | D3ZR49\|D3ZR49 | 10090.ENSMUSP00000073751 | 10090.ENSMUSP00000008907 | 163 |
| Q5I0D7\|PEPD | Q5MYT7\|OAS3 | 10090.ENSMUSP00000075683 | 10090.ENSMUSP00000035588 | 400 |
| Q8CHJ1\|PIGU | D3ZR49\|D3ZR49 | 10090.ENSMUSP00000076816 | 10090.ENSMUSP00000008907 | 175 |
| Q8CHJ1\|PIGU | Q5MYT7\|OAS3 | 10090.ENSMUSP00000076816 | 10090.ENSMUSP00000035588 | 224 |
| A0A0G2K1Q9\|A0A0G2K1Q9 | Q5I0D7\|PEPD | 10090.ENSMUSP00000079098 | 10090.ENSMUSP00000075683 | 158 |
| A0A0G2JU45\|A0A0G2JU45 | Q5MYT7\|OAS3 | 10090.ENSMUSP00000112121 | 10090.ENSMUSP00000035588 | 229 |
| A0A0G2JU45\|A0A0G2JU45 | B0BNI2\|B0BNI2 | 10090.ENSMUSP00000112121 | 10090.ENSMUSP00000043315 | 172 |
| A0A0G2JU45\|A0A0G2JU45 | B0BNB5\|B0BNB5 | 10090.ENSMUSP00000112121 | 10090.ENSMUSP00000046732 | 157 |
| Q5UT80\|Q5UT80 | P18211\|HB2D | 10116.ENSRNOP00000000523 | 10116.ENSRNOP00000000522 | 946 |
| Q2TGK3\|Q2TGK3 | D3ZV54\|D3ZV54 | 10116.ENSRNOP00000005817 | 10116.ENSRNOP00000001605 | 188 |
| G3V6P6\|G3V6P6 | Q498C9\|Q498C9 | 10116.ENSRNOP00000007367 | 10116.ENSRNOP00000000253 | 172 |
| G3V6P6\|G3V6P6 | Q62662\|FRK | 10116.ENSRNOP00000007367 | 10116.ENSRNOP00000000653 | 154 |
| G3V6P6\|G3V6P6 | D3ZV54\|D3ZV54 | 10116.ENSRNOP00000007367 | 10116.ENSRNOP00000001605 | 193 |
| G3V6P6\|G3V6P6 | A0A0G2K402\|A0A0G2K402 | 10116.ENSRNOP00000007367 | 10116.ENSRNOP00000007103 | 312 |
| Q5XI55\|NGLY1 | Q5BKC6\|HBAP1 | 10116.ENSRNOP00000008289 | 10116.ENSRNOP00000003060 | 159 |
| Q5RKH2\|Q5RKH2 | Q6IFV1\|K1C14 | 10116.ENSRNOP00000008525 | 10116.ENSRNOP00000005285 | 155 |
| Q4VBH2\|Q4VBH2 | D3ZCR4\|D3ZCR4 | 10116.ENSRNOP00000008527 | 10116.ENSRNOP00000005170 | 589 |
| P30904\|MIF | Q5RKH2\|Q5RKH2 | 10116.ENSRNOP00000008608 | 10116.ENSRNOP00000008525 | 183 |
| Q66HG4\|GALM | Q5RKH2\|Q5RKH2 | 10116.ENSRNOP00000009221 | 10116.ENSRNOP00000008525 | 995 |
| P31214\|S5A2 | Q66HG4\|GALM | 10116.ENSRNOP00000009254 | 10116.ENSRNOP00000009221 | 243 |
| B5DEI2\|B5DEI2 | P30904\|MIF | 10116.ENSRNOP00000009520 | 10116.ENSRNOP00000008608 | 900 |
| D3ZV30\|D3ZV30 | D3ZV54\|D3ZV54 | 10116.ENSRNOP00000009746 | 10116.ENSRNOP00000001605 | 661 |
| D3ZV30\|D3ZV30 | Q5RKH2\|Q5RKH2 | 10116.ENSRNOP00000009746 | 10116.ENSRNOP00000008525 | 270 |
| D3ZV30\|D3ZV30 | Q4VBH2\|Q4VBH2 | 10116.ENSRNOP00000009746 | 10116.ENSRNOP00000008527 | 213 |
| A0A140TAA1\|A0A140TAA1 | A0A0G2JY11\|A0A0G2JY11 | 10116.ENSRNOP00000010028 | 10116.ENSRNOP00000007328 | 183 |
| Q68FX7\|THOC5 | Q62662\|FRK | 10116.ENSRNOP00000011281 | 10116.ENSRNOP00000000653 | 437 |
| A3KNA0\|A3KNA0 | G3V6P6\|G3V6P6 | 10116.ENSRNOP00000011950 | 10116.ENSRNOP00000007367 | 220 |
| A3KNA0\|A3KNA0 | Q9ER31\|ENTP6 | 10116.ENSRNOP00000011950 | 10116.ENSRNOP00000009946 | 487 |
| A0A0G2JV51\|A0A0G2JV51 | D3ZV54\|D3ZV54 | 10116.ENSRNOP00000012094 | 10116.ENSRNOP00000001605 | 994 |
| A0A0G2JV51\|A0A0G2JV51 | F1LR42\|F1LR42 | 10116.ENSRNOP00000012094 | 10116.ENSRNOP00000004740 | 169 |
| A0A0G2JV51\|A0A0G2JV51 | G3V6P6\|G3V6P6 | 10116.ENSRNOP00000012094 | 10116.ENSRNOP00000007367 | 150 |
| A0A0G2JV51\|A0A0G2JV51 | D3ZV30\|D3ZV30 | 10116.ENSRNOP00000012094 | 10116.ENSRNOP00000009746 | 796 |
| A0A0G2JV51\|A0A0G2JV51 | A3KNA0\|A3KNA0 | 10116.ENSRNOP00000012094 | 10116.ENSRNOP00000011950 | 188 |
| D3ZBN3\|D3ZBN3 | Q62662\|FRK | 10116.ENSRNOP00000012328 | 10116.ENSRNOP00000000653 | 536 |
| D3ZBN3\|D3ZBN3 | F1LR42\|F1LR42 | 10116.ENSRNOP00000012328 | 10116.ENSRNOP00000004740 | 249 |
| D3ZBN3\|D3ZBN3 | G3V6P6\|G3V6P6 | 10116.ENSRNOP00000012328 | 10116.ENSRNOP00000007367 | 154 |
| P17164\|FUCO | Q5RKH2\|Q5RKH2 | 10116.ENSRNOP00000012455 | 10116.ENSRNOP00000008525 | 167 |
| P17164\|FUCO | G3V757\|G3V757 | 10116.ENSRNOP00000012455 | 10116.ENSRNOP00000012176 | 217 |
| Q4QQW4\|HDAC1 | Q6MGB6\|RING1 | 10116.ENSRNOP00000012854 | 10116.ENSRNOP00000000543 | 223 |
| Q4QQW4\|HDAC1 | Q62662\|FRK | 10116.ENSRNOP00000012854 | 10116.ENSRNOP00000000653 | 491 |
| Q4QQW4\|HDAC1 | D3ZV30\|D3ZV30 | 10116.ENSRNOP00000012854 | 10116.ENSRNOP00000009746 | 161 |
| A0A0G2K4N5\|A0A0G2K4N5 | Q9JJ46\|EBP | 10116.ENSRNOP00000013116 | 10116.ENSRNOP00000007015 | 416 |
| A0A0G2K4N5\|A0A0G2K4N5 | P24464\|CP4AC | 10116.ENSRNOP00000013116 | 10116.ENSRNOP00000012448 | 289 |
| P62078\|TIM8B | Q5U1W6\|Q5U1W6 | 10116.ENSRNOP00000013188 | 10116.ENSRNOP00000005997 | 168 |
| P62078\|TIM8B | Q9JKW1\|TIM22 | 10116.ENSRNOP00000013188 | 10116.ENSRNOP00000010779 | 772 |
| P50878\|RL4 | D3ZV54\|D3ZV54 | 10116.ENSRNOP00000013462 | 10116.ENSRNOP00000001605 | 510 |
| P50878\|RL4 | G3V6P6\|G3V6P6 | 10116.ENSRNOP00000013462 | 10116.ENSRNOP00000007367 | 262 |
| P50878\|RL4 | D3ZV30\|D3ZV30 | 10116.ENSRNOP00000013462 | 10116.ENSRNOP00000009746 | 892 |
| P50878\|RL4 | A0A0G2JV51\|A0A0G2JV51 | 10116.ENSRNOP00000013462 | 10116.ENSRNOP00000012094 | 507 |
| D3ZY44\|D3ZY44 | Q64380\|SARDH | 10116.ENSRNOP00000013548 | 10116.ENSRNOP00000009555 | 214 |
| D3ZY44\|D3ZY44 | D3ZV30\|D3ZV30 | 10116.ENSRNOP00000013548 | 10116.ENSRNOP00000009746 | 713 |
| D3ZY44\|D3ZY44 | P50878\|RL4 | 10116.ENSRNOP00000013548 | 10116.ENSRNOP00000013462 | 944 |
| P84039\|ENPP5 | A0A0G2K402\|A0A0G2K402 | 10116.ENSRNOP00000013703 | 10116.ENSRNOP00000007103 | 169 |
| P84039\|ENPP5 | Q9ER31\|ENTP6 | 10116.ENSRNOP00000013703 | 10116.ENSRNOP00000009946 | 247 |
| Q6EV70\|OFUT1 | G3V757\|G3V757 | 10116.ENSRNOP00000013730 | 10116.ENSRNOP00000012176 | 540 |
| D4AC65\|D4AC65 | Q62662\|FRK | 10116.ENSRNOP00000014401 | 10116.ENSRNOP00000000653 | 188 |
| D4AC65\|D4AC65 | D3ZBN3\|D3ZBN3 | 10116.ENSRNOP00000014401 | 10116.ENSRNOP00000012328 | 188 |
| Q68FU7\|COQ6 | Q6IFV1\|K1C14 | 10116.ENSRNOP00000014914 | 10116.ENSRNOP00000005285 | 640 |
| Q68FU7\|COQ6 | P30904\|MIF | 10116.ENSRNOP00000014914 | 10116.ENSRNOP00000008608 | 161 |
| Q68FU7\|COQ6 | P24464\|CP4AC | 10116.ENSRNOP00000014914 | 10116.ENSRNOP00000012448 | 317 |
| Q68FU7\|COQ6 | Q4QQW4\|HDAC1 | 10116.ENSRNOP00000014914 | 10116.ENSRNOP00000012854 | 286 |
| Q68FU7\|COQ6 | A0A0G2K4N5\|A0A0G2K4N5 | 10116.ENSRNOP00000014914 | 10116.ENSRNOP00000013116 | 317 |
| O08701\|ARGI2 | Q64380\|SARDH | 10116.ENSRNOP00000015083 | 10116.ENSRNOP00000009555 | 211 |
| O08701\|ARGI2 | D3ZV30\|D3ZV30 | 10116.ENSRNOP00000015083 | 10116.ENSRNOP00000009746 | 163 |
| O08701\|ARGI2 | Q4QQW4\|HDAC1 | 10116.ENSRNOP00000015083 | 10116.ENSRNOP00000012854 | 243 |
| D3ZJF9\|D3ZJF9 | A0A0G2JY11\|A0A0G2JY11 | 10116.ENSRNOP00000015494 | 10116.ENSRNOP00000007328 | 353 |
| D3ZJF9\|D3ZJF9 | Q66HG4\|GALM | 10116.ENSRNOP00000015494 | 10116.ENSRNOP00000009221 | 904 |
| P97612\|FAAH1 | Q5HZE4\|MTNA | 10116.ENSRNOP00000015961 | 10116.ENSRNOP00000003762 | 171 |
| P97612\|FAAH1 | O08701\|ARGI2 | 10116.ENSRNOP00000015961 | 10116.ENSRNOP00000015083 | 476 |
| B1H2A6\|B1H2A6 | A0A0G2JV51\|A0A0G2JV51 | 10116.ENSRNOP00000016163 | 10116.ENSRNOP00000012094 | 161 |
| D4A4J0\|D4A4J0 | D3ZCR4\|D3ZCR4 | 10116.ENSRNOP00000016288 | 10116.ENSRNOP00000005170 | 222 |
| D4A4J0\|D4A4J0 | D3ZV30\|D3ZV30 | 10116.ENSRNOP00000016288 | 10116.ENSRNOP00000009746 | 293 |
| D4A4J0\|D4A4J0 | P24464\|CP4AC | 10116.ENSRNOP00000016288 | 10116.ENSRNOP00000012448 | 277 |
| D4A4J0\|D4A4J0 | Q4QQW4\|HDAC1 | 10116.ENSRNOP00000016288 | 10116.ENSRNOP00000012854 | 472 |
| Q9Z339\|GSTO1 | Q5HZE4\|MTNA | 10116.ENSRNOP00000016851 | 10116.ENSRNOP00000003762 | 290 |
| Q9Z339\|GSTO1 | P62078\|TIM8B | 10116.ENSRNOP00000016851 | 10116.ENSRNOP00000013188 | 179 |
| Q9Z339\|GSTO1 | P97612\|FAAH1 | 10116.ENSRNOP00000016851 | 10116.ENSRNOP00000015961 | 179 |
| B1WBY7\|B1WBY7 | A0A0G2K4N5\|A0A0G2K4N5 | 10116.ENSRNOP00000017268 | 10116.ENSRNOP00000013116 | 229 |
| B5DF79\|B5DF79 | P17164\|FUCO | 10116.ENSRNOP00000017601 | 10116.ENSRNOP00000012455 | 539 |
| A0A0G2JU12\|A0A0G2JU12 | Q9Z339\|GSTO1 | 10116.ENSRNOP00000017785 | 10116.ENSRNOP00000016851 | 943 |
| Q5XIB2\|CWC27 | A3KNA0\|A3KNA0 | 10116.ENSRNOP00000017832 | 10116.ENSRNOP00000011950 | 242 |
| Q5XIB2\|CWC27 | Q4QQW4\|HDAC1 | 10116.ENSRNOP00000017832 | 10116.ENSRNOP00000012854 | 182 |
| Q5XIB2\|CWC27 | P50878\|RL4 | 10116.ENSRNOP00000017832 | 10116.ENSRNOP00000013462 | 191 |
| Q5XIB2\|CWC27 | D3ZY44\|D3ZY44 | 10116.ENSRNOP00000017832 | 10116.ENSRNOP00000013548 | 222 |
| B5DFG9\|B5DFG9 | Q62662\|FRK | 10116.ENSRNOP00000017938 | 10116.ENSRNOP00000000653 | 163 |
| B5DFG9\|B5DFG9 | D3ZBN3\|D3ZBN3 | 10116.ENSRNOP00000017938 | 10116.ENSRNOP00000012328 | 163 |
| P45479\|PPT1 | P17164\|FUCO | 10116.ENSRNOP00000017998 | 10116.ENSRNOP00000012455 | 204 |
| P45479\|PPT1 | B5DF79\|B5DF79 | 10116.ENSRNOP00000017998 | 10116.ENSRNOP00000017601 | 200 |
| D3ZUC2\|D3ZUC2 | Q6MGB6\|RING1 | 10116.ENSRNOP00000018061 | 10116.ENSRNOP00000000543 | 170 |
| D3ZUC2\|D3ZUC2 | G3V6P6\|G3V6P6 | 10116.ENSRNOP00000018061 | 10116.ENSRNOP00000007367 | 182 |
| D3ZUC2\|D3ZUC2 | B1H2A6\|B1H2A6 | 10116.ENSRNOP00000018061 | 10116.ENSRNOP00000016163 | 193 |
| D3ZUC2\|D3ZUC2 | D4A4J0\|D4A4J0 | 10116.ENSRNOP00000018061 | 10116.ENSRNOP00000016288 | 208 |
| Q9EQN5\|SMBP2 | Q6MGB6\|RING1 | 10116.ENSRNOP00000018487 | 10116.ENSRNOP00000000543 | 469 |
| Q9EQN5\|SMBP2 | Q62662\|FRK | 10116.ENSRNOP00000018487 | 10116.ENSRNOP00000000653 | 168 |
| Q9EQN5\|SMBP2 | G3V6P6\|G3V6P6 | 10116.ENSRNOP00000018487 | 10116.ENSRNOP00000007367 | 161 |
| Q9EQN5\|SMBP2 | A0A0G2JV51\|A0A0G2JV51 | 10116.ENSRNOP00000018487 | 10116.ENSRNOP00000012094 | 497 |
| Q9EQN5\|SMBP2 | D4A4J0\|D4A4J0 | 10116.ENSRNOP00000018487 | 10116.ENSRNOP00000016288 | 208 |
| Q6AYC4\|CAPG | B5DF79\|B5DF79 | 10116.ENSRNOP00000018562 | 10116.ENSRNOP00000017601 | 192 |
| P20611\|PPAL | P45479\|PPT1 | 10116.ENSRNOP00000018620 | 10116.ENSRNOP00000017998 | 159 |
| Q63279\|K1C19 | Q6IFV1\|K1C14 | 10116.ENSRNOP00000019133 | 10116.ENSRNOP00000005285 | 547 |
| Q63279\|K1C19 | Q9Z339\|GSTO1 | 10116.ENSRNOP00000019133 | 10116.ENSRNOP00000016851 | 153 |
| D4A604\|D4A604 | P84039\|ENPP5 | 10116.ENSRNOP00000019761 | 10116.ENSRNOP00000013703 | 185 |
| F1LQI1\|F1LQI1 | D3ZY44\|D3ZY44 | 10116.ENSRNOP00000020192 | 10116.ENSRNOP00000013548 | 186 |
| F1LQI1\|F1LQI1 | Q9Z339\|GSTO1 | 10116.ENSRNOP00000020192 | 10116.ENSRNOP00000016851 | 226 |
| F1LQI1\|F1LQI1 | P20611\|PPAL | 10116.ENSRNOP00000020192 | 10116.ENSRNOP00000018620 | 301 |
| D3ZUX7\|D3ZUX7 | Q5XI55\|NGLY1 | 10116.ENSRNOP00000020313 | 10116.ENSRNOP00000008289 | 153 |
| D3ZUX7\|D3ZUX7 | P24464\|CP4AC | 10116.ENSRNOP00000020313 | 10116.ENSRNOP00000012448 | 460 |
| D3ZUX7\|D3ZUX7 | A0A0G2K4N5\|A0A0G2K4N5 | 10116.ENSRNOP00000020313 | 10116.ENSRNOP00000013116 | 460 |
| D3ZUX7\|D3ZUX7 | O70597\|PX11A | 10116.ENSRNOP00000020313 | 10116.ENSRNOP00000020229 | 546 |
| Q6AYS4\|FUCO2 | Q5RKH2\|Q5RKH2 | 10116.ENSRNOP00000020946 | 10116.ENSRNOP00000008525 | 167 |
| Q6AYS4\|FUCO2 | G3V757\|G3V757 | 10116.ENSRNOP00000020946 | 10116.ENSRNOP00000012176 | 217 |
| Q6AYS4\|FUCO2 | P17164\|FUCO | 10116.ENSRNOP00000020946 | 10116.ENSRNOP00000012455 | 169 |
| P0C588\|CNNM4 | F1LQI1\|F1LQI1 | 10116.ENSRNOP00000021434 | 10116.ENSRNOP00000020192 | 155 |
| Q9WVK3\|PECR | Q9JJ46\|EBP | 10116.ENSRNOP00000021512 | 10116.ENSRNOP00000007015 | 335 |
| Q9WVK3\|PECR | Q66HG4\|GALM | 10116.ENSRNOP00000021512 | 10116.ENSRNOP00000009221 | 395 |
| Q9WVK3\|PECR | Q64380\|SARDH | 10116.ENSRNOP00000021512 | 10116.ENSRNOP00000009555 | 453 |
| Q9WVK3\|PECR | A0A0G2K4N5\|A0A0G2K4N5 | 10116.ENSRNOP00000021512 | 10116.ENSRNOP00000013116 | 286 |
| Q9WVK3\|PECR | O08701\|ARGI2 | 10116.ENSRNOP00000021512 | 10116.ENSRNOP00000015083 | 161 |
| Q9WVK3\|PECR | Q9Z339\|GSTO1 | 10116.ENSRNOP00000021512 | 10116.ENSRNOP00000016851 | 175 |
| Q9WVK3\|PECR | D4A604\|D4A604 | 10116.ENSRNOP00000021512 | 10116.ENSRNOP00000019761 | 156 |
| Q9WVK3\|PECR | O70597\|PX11A | 10116.ENSRNOP00000021512 | 10116.ENSRNOP00000020229 | 222 |
| Q9WVK3\|PECR | D3ZUX7\|D3ZUX7 | 10116.ENSRNOP00000021512 | 10116.ENSRNOP00000020313 | 503 |
| D4ACK7\|D4ACK7 | F1LQI1\|F1LQI1 | 10116.ENSRNOP00000021591 | 10116.ENSRNOP00000020192 | 155 |
| Q5EB90\|Q5EB90 | D3ZV54\|D3ZV54 | 10116.ENSRNOP00000021639 | 10116.ENSRNOP00000001605 | 307 |
| Q5EB90\|Q5EB90 | Q62931\|GOSR1 | 10116.ENSRNOP00000021639 | 10116.ENSRNOP00000005283 | 232 |
| Q5EB90\|Q5EB90 | Q5RKH2\|Q5RKH2 | 10116.ENSRNOP00000021639 | 10116.ENSRNOP00000008525 | 231 |
| Q5EB90\|Q5EB90 | Q4VBH2\|Q4VBH2 | 10116.ENSRNOP00000021639 | 10116.ENSRNOP00000008527 | 223 |
| Q5EB90\|Q5EB90 | Q64380\|SARDH | 10116.ENSRNOP00000021639 | 10116.ENSRNOP00000009555 | 153 |
| Q5EB90\|Q5EB90 | D3ZV30\|D3ZV30 | 10116.ENSRNOP00000021639 | 10116.ENSRNOP00000009746 | 960 |
| Q5EB90\|Q5EB90 | A0A0G2JV51\|A0A0G2JV51 | 10116.ENSRNOP00000021639 | 10116.ENSRNOP00000012094 | 293 |
| Q5EB90\|Q5EB90 | Q4QQW4\|HDAC1 | 10116.ENSRNOP00000021639 | 10116.ENSRNOP00000012854 | 251 |
| Q5EB90\|Q5EB90 | P50878\|RL4 | 10116.ENSRNOP00000021639 | 10116.ENSRNOP00000013462 | 943 |
| Q5EB90\|Q5EB90 | D3ZY44\|D3ZY44 | 10116.ENSRNOP00000021639 | 10116.ENSRNOP00000013548 | 891 |
| Q5EB90\|Q5EB90 | D4A4J0\|D4A4J0 | 10116.ENSRNOP00000021639 | 10116.ENSRNOP00000016288 | 938 |
| Q5EB90\|Q5EB90 | Q5XIB2\|CWC27 | 10116.ENSRNOP00000021639 | 10116.ENSRNOP00000017832 | 190 |
| Q5EB90\|Q5EB90 | D3ZUC2\|D3ZUC2 | 10116.ENSRNOP00000021639 | 10116.ENSRNOP00000018061 | 169 |
| Q5EB90\|Q5EB90 | Q9EQN5\|SMBP2 | 10116.ENSRNOP00000021639 | 10116.ENSRNOP00000018487 | 169 |
| P60825\|CIRBP | Q498C9\|Q498C9 | 10116.ENSRNOP00000021648 | 10116.ENSRNOP00000000253 | 172 |
| P60825\|CIRBP | Q62662\|FRK | 10116.ENSRNOP00000021648 | 10116.ENSRNOP00000000653 | 154 |
| P60825\|CIRBP | D3ZV54\|D3ZV54 | 10116.ENSRNOP00000021648 | 10116.ENSRNOP00000001605 | 193 |
| P60825\|CIRBP | A0A0G2K402\|A0A0G2K402 | 10116.ENSRNOP00000021648 | 10116.ENSRNOP00000007103 | 312 |
| P60825\|CIRBP | A3KNA0\|A3KNA0 | 10116.ENSRNOP00000021648 | 10116.ENSRNOP00000011950 | 220 |
| P60825\|CIRBP | A0A0G2JV51\|A0A0G2JV51 | 10116.ENSRNOP00000021648 | 10116.ENSRNOP00000012094 | 150 |
| P60825\|CIRBP | D3ZBN3\|D3ZBN3 | 10116.ENSRNOP00000021648 | 10116.ENSRNOP00000012328 | 154 |
| P60825\|CIRBP | Q4QQW4\|HDAC1 | 10116.ENSRNOP00000021648 | 10116.ENSRNOP00000012854 | 198 |
| P60825\|CIRBP | P50878\|RL4 | 10116.ENSRNOP00000021648 | 10116.ENSRNOP00000013462 | 262 |
| P60825\|CIRBP | D3ZUC2\|D3ZUC2 | 10116.ENSRNOP00000021648 | 10116.ENSRNOP00000018061 | 182 |
| P60825\|CIRBP | Q9EQN5\|SMBP2 | 10116.ENSRNOP00000021648 | 10116.ENSRNOP00000018487 | 161 |
| P16303\|CES1D | F1LQI1\|F1LQI1 | 10116.ENSRNOP00000021812 | 10116.ENSRNOP00000020192 | 209 |
| Q9Z1L0\|PK3CB | Q62662\|FRK | 10116.ENSRNOP00000022179 | 10116.ENSRNOP00000000653 | 764 |
| Q9Z1L0\|PK3CB | D3ZCR4\|D3ZCR4 | 10116.ENSRNOP00000022179 | 10116.ENSRNOP00000005170 | 154 |
| Q9Z1L0\|PK3CB | D3ZBN3\|D3ZBN3 | 10116.ENSRNOP00000022179 | 10116.ENSRNOP00000012328 | 585 |
| Q9Z1L0\|PK3CB | Q4QQW4\|HDAC1 | 10116.ENSRNOP00000022179 | 10116.ENSRNOP00000012854 | 204 |
| Q9Z1L0\|PK3CB | D4A4J0\|D4A4J0 | 10116.ENSRNOP00000022179 | 10116.ENSRNOP00000016288 | 177 |
| Q9Z1L0\|PK3CB | D3ZUC2\|D3ZUC2 | 10116.ENSRNOP00000022179 | 10116.ENSRNOP00000018061 | 256 |
| Q9Z1L0\|PK3CB | F1M365\|F1M365 | 10116.ENSRNOP00000022179 | 10116.ENSRNOP00000018071 | 150 |
| Q9Z1L0\|PK3CB | Q9EQN5\|SMBP2 | 10116.ENSRNOP00000022179 | 10116.ENSRNOP00000018487 | 301 |
| D3ZPN5\|D3ZPN5 | G3V6P6\|G3V6P6 | 10116.ENSRNOP00000022462 | 10116.ENSRNOP00000007367 | 157 |
| D3ZPN5\|D3ZPN5 | Q4VBH2\|Q4VBH2 | 10116.ENSRNOP00000022462 | 10116.ENSRNOP00000008527 | 276 |
| D3ZPN5\|D3ZPN5 | Q68FX7\|THOC5 | 10116.ENSRNOP00000022462 | 10116.ENSRNOP00000011281 | 182 |
| D3ZPN5\|D3ZPN5 | B1H2A6\|B1H2A6 | 10116.ENSRNOP00000022462 | 10116.ENSRNOP00000016163 | 383 |
| D3ZPN5\|D3ZPN5 | D3ZUC2\|D3ZUC2 | 10116.ENSRNOP00000022462 | 10116.ENSRNOP00000018061 | 176 |
| D3ZPN5\|D3ZPN5 | Q9EQN5\|SMBP2 | 10116.ENSRNOP00000022462 | 10116.ENSRNOP00000018487 | 176 |
| D3ZPN5\|D3ZPN5 | P60825\|CIRBP | 10116.ENSRNOP00000022462 | 10116.ENSRNOP00000021648 | 157 |
| Q5U3Z3\|ISOC2 | D3ZMN2\|D3ZMN2 | 10116.ENSRNOP00000022621 | 10116.ENSRNOP00000015130 | 217 |
| Q63619\|COQ7 | Q68FU7\|COQ6 | 10116.ENSRNOP00000022988 | 10116.ENSRNOP00000014914 | 878 |
| P15205\|MAP1B | Q63525\|NUDC | 10116.ENSRNOP00000023460 | 10116.ENSRNOP00000009933 | 204 |
| P15205\|MAP1B | B1H2A6\|B1H2A6 | 10116.ENSRNOP00000023460 | 10116.ENSRNOP00000016163 | 674 |
| P15205\|MAP1B | D4A604\|D4A604 | 10116.ENSRNOP00000023460 | 10116.ENSRNOP00000019761 | 156 |
| P07150\|ANXA1 | B5DF79\|B5DF79 | 10116.ENSRNOP00000023664 | 10116.ENSRNOP00000017601 | 175 |
| P07150\|ANXA1 | Q6AYC4\|CAPG | 10116.ENSRNOP00000023664 | 10116.ENSRNOP00000018562 | 246 |
| P07150\|ANXA1 | P20611\|PPAL | 10116.ENSRNOP00000023664 | 10116.ENSRNOP00000018620 | 200 |
| P07150\|ANXA1 | Q63279\|K1C19 | 10116.ENSRNOP00000023664 | 10116.ENSRNOP00000019133 | 155 |
| P07150\|ANXA1 | P16303\|CES1D | 10116.ENSRNOP00000023664 | 10116.ENSRNOP00000021812 | 160 |
| Q8CG45\|ARK72 | Q68FU7\|COQ6 | 10116.ENSRNOP00000024063 | 10116.ENSRNOP00000014914 | 181 |
| Q8CG45\|ARK72 | F1LQI1\|F1LQI1 | 10116.ENSRNOP00000024063 | 10116.ENSRNOP00000020192 | 169 |
| Q8CG45\|ARK72 | Q9WVK3\|PECR | 10116.ENSRNOP00000024063 | 10116.ENSRNOP00000021512 | 173 |
| D3ZCV0\|D3ZCV0 | Q62662\|FRK | 10116.ENSRNOP00000024098 | 10116.ENSRNOP00000000653 | 563 |
| D3ZCV0\|D3ZCV0 | Q68FU7\|COQ6 | 10116.ENSRNOP00000024098 | 10116.ENSRNOP00000014914 | 216 |
| D3ZCV0\|D3ZCV0 | Q6AYC4\|CAPG | 10116.ENSRNOP00000024098 | 10116.ENSRNOP00000018562 | 176 |
| D3ZCV0\|D3ZCV0 | Q9Z1L0\|PK3CB | 10116.ENSRNOP00000024098 | 10116.ENSRNOP00000022179 | 571 |
| G3V8C0\|G3V8C0 | P07150\|ANXA1 | 10116.ENSRNOP00000024367 | 10116.ENSRNOP00000023664 | 179 |
| B2RYP8\|B2RYP8 | Q62662\|FRK | 10116.ENSRNOP00000024573 | 10116.ENSRNOP00000000653 | 221 |
| B2RYP8\|B2RYP8 | B1WBY7\|B1WBY7 | 10116.ENSRNOP00000024573 | 10116.ENSRNOP00000017268 | 613 |
| A9CMA7\|A9CMA7 | Q62931\|GOSR1 | 10116.ENSRNOP00000024602 | 10116.ENSRNOP00000005283 | 198 |
| A9CMA7\|A9CMA7 | A0A0G2K402\|A0A0G2K402 | 10116.ENSRNOP00000024602 | 10116.ENSRNOP00000007103 | 301 |
| A9CMA7\|A9CMA7 | D4A4J0\|D4A4J0 | 10116.ENSRNOP00000024602 | 10116.ENSRNOP00000016288 | 900 |
| A9CMA7\|A9CMA7 | Q5EB90\|Q5EB90 | 10116.ENSRNOP00000024602 | 10116.ENSRNOP00000021639 | 903 |
| F1LQC8\|F1LQC8 | D3ZV30\|D3ZV30 | 10116.ENSRNOP00000025026 | 10116.ENSRNOP00000009746 | 304 |
| F1LQC8\|F1LQC8 | Q4QQW4\|HDAC1 | 10116.ENSRNOP00000025026 | 10116.ENSRNOP00000012854 | 299 |
| F1LQC8\|F1LQC8 | D4A4J0\|D4A4J0 | 10116.ENSRNOP00000025026 | 10116.ENSRNOP00000016288 | 221 |
| F1LQC8\|F1LQC8 | Q5XIB2\|CWC27 | 10116.ENSRNOP00000025026 | 10116.ENSRNOP00000017832 | 194 |
| F1LQC8\|F1LQC8 | Q5EB90\|Q5EB90 | 10116.ENSRNOP00000025026 | 10116.ENSRNOP00000021639 | 598 |
| F1LQC8\|F1LQC8 | A9CMA7\|A9CMA7 | 10116.ENSRNOP00000025026 | 10116.ENSRNOP00000024602 | 708 |
| Q64550\|UD11 | P24464\|CP4AC | 10116.ENSRNOP00000025045 | 10116.ENSRNOP00000012448 | 908 |
| Q64550\|UD11 | D3ZY44\|D3ZY44 | 10116.ENSRNOP00000025045 | 10116.ENSRNOP00000013548 | 209 |
| Q64550\|UD11 | Q9Z339\|GSTO1 | 10116.ENSRNOP00000025045 | 10116.ENSRNOP00000016851 | 194 |
| Q64550\|UD11 | A0A0G2JU12\|A0A0G2JU12 | 10116.ENSRNOP00000025045 | 10116.ENSRNOP00000017785 | 150 |
| Q64550\|UD11 | P16303\|CES1D | 10116.ENSRNOP00000025045 | 10116.ENSRNOP00000021812 | 923 |
| D3ZU51\|D3ZU51 | Q5EB90\|Q5EB90 | 10116.ENSRNOP00000025413 | 10116.ENSRNOP00000021639 | 189 |
| D3ZHR2\|ABCD1 | O70597\|PX11A | 10116.ENSRNOP00000025863 | 10116.ENSRNOP00000020229 | 351 |
| D3ZHR2\|ABCD1 | Q9WVK3\|PECR | 10116.ENSRNOP00000025863 | 10116.ENSRNOP00000021512 | 185 |
| Q9EQV6\|TPP1 | O70489\|PPT2 | 10116.ENSRNOP00000026280 | 10116.ENSRNOP00000000497 | 220 |
| Q9EQV6\|TPP1 | P17164\|FUCO | 10116.ENSRNOP00000026280 | 10116.ENSRNOP00000012455 | 250 |
| Q9EQV6\|TPP1 | P45479\|PPT1 | 10116.ENSRNOP00000026280 | 10116.ENSRNOP00000017998 | 725 |
| Q9EQV6\|TPP1 | P20611\|PPAL | 10116.ENSRNOP00000026280 | 10116.ENSRNOP00000018620 | 416 |
| Q9EQV6\|TPP1 | Q6AYS4\|FUCO2 | 10116.ENSRNOP00000026280 | 10116.ENSRNOP00000020946 | 540 |
| P24050\|RS5 | D3ZV54\|D3ZV54 | 10116.ENSRNOP00000026528 | 10116.ENSRNOP00000001605 | 584 |
| P24050\|RS5 | A0A0G2K402\|A0A0G2K402 | 10116.ENSRNOP00000026528 | 10116.ENSRNOP00000007103 | 228 |
| P24050\|RS5 | P30904\|MIF | 10116.ENSRNOP00000026528 | 10116.ENSRNOP00000008608 | 176 |
| P24050\|RS5 | D3ZV30\|D3ZV30 | 10116.ENSRNOP00000026528 | 10116.ENSRNOP00000009746 | 893 |
| P24050\|RS5 | Q63525\|NUDC | 10116.ENSRNOP00000026528 | 10116.ENSRNOP00000009933 | 201 |
| P24050\|RS5 | A0A0G2JV51\|A0A0G2JV51 | 10116.ENSRNOP00000026528 | 10116.ENSRNOP00000012094 | 639 |
| P24050\|RS5 | P62078\|TIM8B | 10116.ENSRNOP00000026528 | 10116.ENSRNOP00000013188 | 234 |
| P24050\|RS5 | P50878\|RL4 | 10116.ENSRNOP00000026528 | 10116.ENSRNOP00000013462 | 999 |
| P24050\|RS5 | D3ZY44\|D3ZY44 | 10116.ENSRNOP00000026528 | 10116.ENSRNOP00000013548 | 981 |
| P24050\|RS5 | Q5XIB2\|CWC27 | 10116.ENSRNOP00000026528 | 10116.ENSRNOP00000017832 | 215 |
| P24050\|RS5 | Q5EB90\|Q5EB90 | 10116.ENSRNOP00000026528 | 10116.ENSRNOP00000021639 | 933 |
| D4A3P1\|D4A3P1 | Q498C9\|Q498C9 | 10116.ENSRNOP00000026994 | 10116.ENSRNOP00000000253 | 260 |
| D4A3P1\|D4A3P1 | Q62662\|FRK | 10116.ENSRNOP00000026994 | 10116.ENSRNOP00000000653 | 155 |
| D4A3P1\|D4A3P1 | D3ZV54\|D3ZV54 | 10116.ENSRNOP00000026994 | 10116.ENSRNOP00000001605 | 265 |
| D4A3P1\|D4A3P1 | Q5BKC6\|HBAP1 | 10116.ENSRNOP00000026994 | 10116.ENSRNOP00000003060 | 175 |
| D4A3P1\|D4A3P1 | Q5HZE4\|MTNA | 10116.ENSRNOP00000026994 | 10116.ENSRNOP00000003762 | 208 |
| D4A3P1\|D4A3P1 | Q62931\|GOSR1 | 10116.ENSRNOP00000026994 | 10116.ENSRNOP00000005283 | 244 |
| D4A3P1\|D4A3P1 | Q2TGK3\|Q2TGK3 | 10116.ENSRNOP00000026994 | 10116.ENSRNOP00000005817 | 399 |
| D4A3P1\|D4A3P1 | Q5XI55\|NGLY1 | 10116.ENSRNOP00000026994 | 10116.ENSRNOP00000008289 | 438 |
| D4A3P1\|D4A3P1 | Q5RKH2\|Q5RKH2 | 10116.ENSRNOP00000026994 | 10116.ENSRNOP00000008525 | 185 |
| D4A3P1\|D4A3P1 | D3ZV30\|D3ZV30 | 10116.ENSRNOP00000026994 | 10116.ENSRNOP00000009746 | 279 |
| D4A3P1\|D4A3P1 | A0A0G2JV51\|A0A0G2JV51 | 10116.ENSRNOP00000026994 | 10116.ENSRNOP00000012094 | 275 |
| D4A3P1\|D4A3P1 | D3ZBN3\|D3ZBN3 | 10116.ENSRNOP00000026994 | 10116.ENSRNOP00000012328 | 155 |
| D4A3P1\|D4A3P1 | P24464\|CP4AC | 10116.ENSRNOP00000026994 | 10116.ENSRNOP00000012448 | 168 |
| D4A3P1\|D4A3P1 | Q4QQW4\|HDAC1 | 10116.ENSRNOP00000026994 | 10116.ENSRNOP00000012854 | 163 |
| D4A3P1\|D4A3P1 | A0A0G2K4N5\|A0A0G2K4N5 | 10116.ENSRNOP00000026994 | 10116.ENSRNOP00000013116 | 168 |
| D4A3P1\|D4A3P1 | P50878\|RL4 | 10116.ENSRNOP00000026994 | 10116.ENSRNOP00000013462 | 402 |
| D4A3P1\|D4A3P1 | D3ZY44\|D3ZY44 | 10116.ENSRNOP00000026994 | 10116.ENSRNOP00000013548 | 349 |
| D4A3P1\|D4A3P1 | D4AC65\|D4AC65 | 10116.ENSRNOP00000026994 | 10116.ENSRNOP00000014401 | 191 |
| D4A3P1\|D4A3P1 | Q68FU7\|COQ6 | 10116.ENSRNOP00000026994 | 10116.ENSRNOP00000014914 | 216 |
| D4A3P1\|D4A3P1 | D4A4J0\|D4A4J0 | 10116.ENSRNOP00000026994 | 10116.ENSRNOP00000016288 | 339 |
| D4A3P1\|D4A3P1 | Q9Z339\|GSTO1 | 10116.ENSRNOP00000026994 | 10116.ENSRNOP00000016851 | 157 |
| D4A3P1\|D4A3P1 | Q5XIB2\|CWC27 | 10116.ENSRNOP00000026994 | 10116.ENSRNOP00000017832 | 173 |
| D4A3P1\|D4A3P1 | P45479\|PPT1 | 10116.ENSRNOP00000026994 | 10116.ENSRNOP00000017998 | 158 |
| D4A3P1\|D4A3P1 | D3ZUC2\|D3ZUC2 | 10116.ENSRNOP00000026994 | 10116.ENSRNOP00000018061 | 150 |
| D4A3P1\|D4A3P1 | Q9EQN5\|SMBP2 | 10116.ENSRNOP00000026994 | 10116.ENSRNOP00000018487 | 150 |
| D4A3P1\|D4A3P1 | D4A604\|D4A604 | 10116.ENSRNOP00000026994 | 10116.ENSRNOP00000019761 | 193 |
| D4A3P1\|D4A3P1 | O70597\|PX11A | 10116.ENSRNOP00000026994 | 10116.ENSRNOP00000020229 | 154 |
| D4A3P1\|D4A3P1 | D3ZUX7\|D3ZUX7 | 10116.ENSRNOP00000026994 | 10116.ENSRNOP00000020313 | 189 |
| D4A3P1\|D4A3P1 | P0C588\|CNNM4 | 10116.ENSRNOP00000026994 | 10116.ENSRNOP00000021434 | 209 |
| D4A3P1\|D4A3P1 | D4ACK7\|D4ACK7 | 10116.ENSRNOP00000026994 | 10116.ENSRNOP00000021591 | 209 |
| D4A3P1\|D4A3P1 | Q5EB90\|Q5EB90 | 10116.ENSRNOP00000026994 | 10116.ENSRNOP00000021639 | 218 |
| D4A3P1\|D4A3P1 | Q8CG45\|ARK72 | 10116.ENSRNOP00000026994 | 10116.ENSRNOP00000024063 | 211 |
| D4A3P1\|D4A3P1 | D3ZCV0\|D3ZCV0 | 10116.ENSRNOP00000026994 | 10116.ENSRNOP00000024098 | 169 |
| D4A3P1\|D4A3P1 | B2RYP8\|B2RYP8 | 10116.ENSRNOP00000026994 | 10116.ENSRNOP00000024573 | 163 |
| D4A3P1\|D4A3P1 | P24050\|RS5 | 10116.ENSRNOP00000026994 | 10116.ENSRNOP00000026528 | 416 |
| D3ZHA0\|FLNC | Q62662\|FRK | 10116.ENSRNOP00000027237 | 10116.ENSRNOP00000000653 | 153 |
| D3ZHA0\|FLNC | Q66HG4\|GALM | 10116.ENSRNOP00000027237 | 10116.ENSRNOP00000009221 | 198 |
| D3ZHA0\|FLNC | Q9Z339\|GSTO1 | 10116.ENSRNOP00000027237 | 10116.ENSRNOP00000016851 | 153 |
| D3ZHA0\|FLNC | Q6AYC4\|CAPG | 10116.ENSRNOP00000027237 | 10116.ENSRNOP00000018562 | 169 |
| D3ZHA0\|FLNC | Q9Z1L0\|PK3CB | 10116.ENSRNOP00000027237 | 10116.ENSRNOP00000022179 | 172 |
| D3ZHA0\|FLNC | D3ZCV0\|D3ZCV0 | 10116.ENSRNOP00000027237 | 10116.ENSRNOP00000024098 | 174 |
| D3ZHA0\|FLNC | D4A3P1\|D4A3P1 | 10116.ENSRNOP00000027237 | 10116.ENSRNOP00000026994 | 169 |
| F1LQ48\|HNRPL | G3V6P6\|G3V6P6 | 10116.ENSRNOP00000027425 | 10116.ENSRNOP00000007367 | 171 |
| F1LQ48\|HNRPL | Q5EB90\|Q5EB90 | 10116.ENSRNOP00000027425 | 10116.ENSRNOP00000021639 | 900 |
| F1LQ48\|HNRPL | P60825\|CIRBP | 10116.ENSRNOP00000027425 | 10116.ENSRNOP00000021648 | 171 |
| F1LQ48\|HNRPL | D4A3P1\|D4A3P1 | 10116.ENSRNOP00000027425 | 10116.ENSRNOP00000026994 | 188 |
| P97590\|LEG7 | G3V757\|G3V757 | 10116.ENSRNOP00000027620 | 10116.ENSRNOP00000012176 | 281 |
| P97590\|LEG7 | P17164\|FUCO | 10116.ENSRNOP00000027620 | 10116.ENSRNOP00000012455 | 223 |
| P97590\|LEG7 | Q6AYS4\|FUCO2 | 10116.ENSRNOP00000027620 | 10116.ENSRNOP00000020946 | 190 |
| O88588\|PACS1 | O08701\|ARGI2 | 10116.ENSRNOP00000027632 | 10116.ENSRNOP00000015083 | 154 |
| D3ZJF7\|D3ZJF7 | F1M365\|F1M365 | 10116.ENSRNOP00000027695 | 10116.ENSRNOP00000018071 | 286 |
| D3ZJF7\|D3ZJF7 | F1LQC8\|F1LQC8 | 10116.ENSRNOP00000027695 | 10116.ENSRNOP00000025026 | 243 |
| Q9Z122\|FADS2 | P18211\|HB2D | 10116.ENSRNOP00000027756 | 10116.ENSRNOP00000000522 | 179 |
| Q9Z122\|FADS2 | D3ZCR4\|D3ZCR4 | 10116.ENSRNOP00000027756 | 10116.ENSRNOP00000005170 | 200 |
| Q9Z122\|FADS2 | Q9JJ46\|EBP | 10116.ENSRNOP00000027756 | 10116.ENSRNOP00000007015 | 188 |
| Q9Z122\|FADS2 | Q64380\|SARDH | 10116.ENSRNOP00000027756 | 10116.ENSRNOP00000009555 | 302 |
| Q9Z122\|FADS2 | Q9Z339\|GSTO1 | 10116.ENSRNOP00000027756 | 10116.ENSRNOP00000016851 | 278 |
| Q9Z122\|FADS2 | D3ZHR2\|ABCD1 | 10116.ENSRNOP00000027756 | 10116.ENSRNOP00000025863 | 900 |
| Q4V7F5\|PIHD1 | Q9Z1L0\|PK3CB | 10116.ENSRNOP00000028022 | 10116.ENSRNOP00000022179 | 181 |
| P23562\|B3AT | Q6IFV1\|K1C14 | 10116.ENSRNOP00000028445 | 10116.ENSRNOP00000005285 | 183 |
| P23562\|B3AT | D4A3P1\|D4A3P1 | 10116.ENSRNOP00000028445 | 10116.ENSRNOP00000026994 | 159 |
| Q5XIE0\|AN32E | Q4QQW4\|HDAC1 | 10116.ENSRNOP00000028744 | 10116.ENSRNOP00000012854 | 218 |
| Q5XIE0\|AN32E | D4A4J0\|D4A4J0 | 10116.ENSRNOP00000028744 | 10116.ENSRNOP00000016288 | 194 |
| P04961\|PCNA | Q62662\|FRK | 10116.ENSRNOP00000028887 | 10116.ENSRNOP00000000653 | 434 |
| P04961\|PCNA | Q6IFV1\|K1C14 | 10116.ENSRNOP00000028887 | 10116.ENSRNOP00000005285 | 239 |
| P04961\|PCNA | Q4VBH2\|Q4VBH2 | 10116.ENSRNOP00000028887 | 10116.ENSRNOP00000008527 | 188 |
| P04961\|PCNA | D3ZV30\|D3ZV30 | 10116.ENSRNOP00000028887 | 10116.ENSRNOP00000009746 | 222 |
| P04961\|PCNA | D3ZBN3\|D3ZBN3 | 10116.ENSRNOP00000028887 | 10116.ENSRNOP00000012328 | 195 |
| P04961\|PCNA | Q4QQW4\|HDAC1 | 10116.ENSRNOP00000028887 | 10116.ENSRNOP00000012854 | 602 |
| P04961\|PCNA | P50878\|RL4 | 10116.ENSRNOP00000028887 | 10116.ENSRNOP00000013462 | 170 |
| P04961\|PCNA | D3ZY44\|D3ZY44 | 10116.ENSRNOP00000028887 | 10116.ENSRNOP00000013548 | 206 |
| P04961\|PCNA | Q68FU7\|COQ6 | 10116.ENSRNOP00000028887 | 10116.ENSRNOP00000014914 | 173 |
| P04961\|PCNA | D4A4J0\|D4A4J0 | 10116.ENSRNOP00000028887 | 10116.ENSRNOP00000016288 | 154 |
| P04961\|PCNA | D3ZUC2\|D3ZUC2 | 10116.ENSRNOP00000028887 | 10116.ENSRNOP00000018061 | 169 |
| P04961\|PCNA | Q9EQN5\|SMBP2 | 10116.ENSRNOP00000028887 | 10116.ENSRNOP00000018487 | 169 |
| P04961\|PCNA | Q63279\|K1C19 | 10116.ENSRNOP00000028887 | 10116.ENSRNOP00000019133 | 221 |
| P04961\|PCNA | Q5EB90\|Q5EB90 | 10116.ENSRNOP00000028887 | 10116.ENSRNOP00000021639 | 337 |
| P04961\|PCNA | Q9Z1L0\|PK3CB | 10116.ENSRNOP00000028887 | 10116.ENSRNOP00000022179 | 365 |
| P04961\|PCNA | P07150\|ANXA1 | 10116.ENSRNOP00000028887 | 10116.ENSRNOP00000023664 | 196 |
| P04961\|PCNA | F1LQC8\|F1LQC8 | 10116.ENSRNOP00000028887 | 10116.ENSRNOP00000025026 | 256 |
| P04961\|PCNA | D4A3P1\|D4A3P1 | 10116.ENSRNOP00000028887 | 10116.ENSRNOP00000026994 | 410 |
| P04961\|PCNA | Q5XIE0\|AN32E | 10116.ENSRNOP00000028887 | 10116.ENSRNOP00000028744 | 516 |
| Q9ER28\|Q9ER28 | Q6AYC4\|CAPG | 10116.ENSRNOP00000031063 | 10116.ENSRNOP00000018562 | 171 |
| Q9ER28\|Q9ER28 | Q9Z1L0\|PK3CB | 10116.ENSRNOP00000031063 | 10116.ENSRNOP00000022179 | 201 |
| D3ZCG2\|D3ZCG2 | B2RYP8\|B2RYP8 | 10116.ENSRNOP00000031911 | 10116.ENSRNOP00000024573 | 183 |
| D3ZCG2\|D3ZCG2 | D4A3P1\|D4A3P1 | 10116.ENSRNOP00000031911 | 10116.ENSRNOP00000026994 | 227 |
| Q5HZA9\|T126A | P62078\|TIM8B | 10116.ENSRNOP00000032681 | 10116.ENSRNOP00000013188 | 521 |
| F1LR52\|F1LR52 | Q62662\|FRK | 10116.ENSRNOP00000033826 | 10116.ENSRNOP00000000653 | 208 |
| F1LR52\|F1LR52 | D3ZV30\|D3ZV30 | 10116.ENSRNOP00000033826 | 10116.ENSRNOP00000009746 | 215 |
| F1LR52\|F1LR52 | D3ZBN3\|D3ZBN3 | 10116.ENSRNOP00000033826 | 10116.ENSRNOP00000012328 | 222 |
| F1LR52\|F1LR52 | Q5EB90\|Q5EB90 | 10116.ENSRNOP00000033826 | 10116.ENSRNOP00000021639 | 163 |
| F1LR52\|F1LR52 | Q64550\|UD11 | 10116.ENSRNOP00000033826 | 10116.ENSRNOP00000025045 | 349 |
| F1LR52\|F1LR52 | D3ZHR2\|ABCD1 | 10116.ENSRNOP00000033826 | 10116.ENSRNOP00000025863 | 477 |
| F1LR52\|F1LR52 | P24050\|RS5 | 10116.ENSRNOP00000033826 | 10116.ENSRNOP00000026528 | 152 |
| F1LR52\|F1LR52 | D4A3P1\|D4A3P1 | 10116.ENSRNOP00000033826 | 10116.ENSRNOP00000026994 | 177 |
| Q5XID7\|ARMX3 | P97612\|FAAH1 | 10116.ENSRNOP00000033859 | 10116.ENSRNOP00000015961 | 197 |
| Q5XID7\|ARMX3 | Q32KJ6\|GALNS | 10116.ENSRNOP00000033859 | 10116.ENSRNOP00000019528 | 221 |
| Q562C7\|PUM3 | D3ZV54\|D3ZV54 | 10116.ENSRNOP00000034834 | 10116.ENSRNOP00000001605 | 938 |
| Q562C7\|PUM3 | G3V6P6\|G3V6P6 | 10116.ENSRNOP00000034834 | 10116.ENSRNOP00000007367 | 178 |
| Q562C7\|PUM3 | D3ZV30\|D3ZV30 | 10116.ENSRNOP00000034834 | 10116.ENSRNOP00000009746 | 363 |
| Q562C7\|PUM3 | A0A0G2JV51\|A0A0G2JV51 | 10116.ENSRNOP00000034834 | 10116.ENSRNOP00000012094 | 931 |
| Q562C7\|PUM3 | P50878\|RL4 | 10116.ENSRNOP00000034834 | 10116.ENSRNOP00000013462 | 438 |
| Q562C7\|PUM3 | D3ZUC2\|D3ZUC2 | 10116.ENSRNOP00000034834 | 10116.ENSRNOP00000018061 | 163 |
| Q562C7\|PUM3 | Q5EB90\|Q5EB90 | 10116.ENSRNOP00000034834 | 10116.ENSRNOP00000021639 | 190 |
| Q562C7\|PUM3 | P60825\|CIRBP | 10116.ENSRNOP00000034834 | 10116.ENSRNOP00000021648 | 178 |
| F1LRQ6\|F1LRQ6 | Q62662\|FRK | 10116.ENSRNOP00000035675 | 10116.ENSRNOP00000000653 | 171 |
| F1LRQ6\|F1LRQ6 | Q5HZE4\|MTNA | 10116.ENSRNOP00000035675 | 10116.ENSRNOP00000003762 | 877 |
| F1LRQ6\|F1LRQ6 | Q9Z1L0\|PK3CB | 10116.ENSRNOP00000035675 | 10116.ENSRNOP00000022179 | 155 |
| F1LRQ6\|F1LRQ6 | F1LQC8\|F1LQC8 | 10116.ENSRNOP00000035675 | 10116.ENSRNOP00000025026 | 247 |
| F1LRQ6\|F1LRQ6 | D4A3P1\|D4A3P1 | 10116.ENSRNOP00000035675 | 10116.ENSRNOP00000026994 | 151 |
| F1LRQ6\|F1LRQ6 | D3ZJF7\|D3ZJF7 | 10116.ENSRNOP00000035675 | 10116.ENSRNOP00000027695 | 301 |
| F1LRQ6\|F1LRQ6 | Q4V7F5\|PIHD1 | 10116.ENSRNOP00000035675 | 10116.ENSRNOP00000028022 | 581 |
| F1LRQ6\|F1LRQ6 | D3ZCG2\|D3ZCG2 | 10116.ENSRNOP00000035675 | 10116.ENSRNOP00000031911 | 152 |
| D3ZGW2\|D3ZGW2 | Q64380\|SARDH | 10116.ENSRNOP00000036180 | 10116.ENSRNOP00000009555 | 193 |
| D3ZGW2\|D3ZGW2 | D4ACK7\|D4ACK7 | 10116.ENSRNOP00000036180 | 10116.ENSRNOP00000021591 | 220 |
| D3ZGW2\|D3ZGW2 | P15205\|MAP1B | 10116.ENSRNOP00000036180 | 10116.ENSRNOP00000023460 | 330 |
| D3ZGW2\|D3ZGW2 | Q64550\|UD11 | 10116.ENSRNOP00000036180 | 10116.ENSRNOP00000025045 | 161 |
| D3ZGW2\|D3ZGW2 | D4A3P1\|D4A3P1 | 10116.ENSRNOP00000036180 | 10116.ENSRNOP00000026994 | 234 |
| F1LRH4\|F1LRH4 | Q5UT80\|Q5UT80 | 10116.ENSRNOP00000037099 | 10116.ENSRNOP00000000523 | 185 |
| F1LRH4\|F1LRH4 | D3ZCV0\|D3ZCV0 | 10116.ENSRNOP00000037099 | 10116.ENSRNOP00000024098 | 221 |
| F1LRH4\|F1LRH4 | P97590\|LEG7 | 10116.ENSRNOP00000037099 | 10116.ENSRNOP00000027620 | 165 |
| D4AA35\|D4AA35 | D3ZY44\|D3ZY44 | 10116.ENSRNOP00000038215 | 10116.ENSRNOP00000013548 | 269 |
| D4AA35\|D4AA35 | P0C588\|CNNM4 | 10116.ENSRNOP00000038215 | 10116.ENSRNOP00000021434 | 228 |
| D4AA35\|D4AA35 | D4ACK7\|D4ACK7 | 10116.ENSRNOP00000038215 | 10116.ENSRNOP00000021591 | 223 |
| Q8CJ11\|AGRG2 | Q6MGB6\|RING1 | 10116.ENSRNOP00000039239 | 10116.ENSRNOP00000000543 | 161 |
| Q8CJ11\|AGRG2 | D3ZBN3\|D3ZBN3 | 10116.ENSRNOP00000039239 | 10116.ENSRNOP00000012328 | 164 |
| Q8CJ11\|AGRG2 | Q5XIE0\|AN32E | 10116.ENSRNOP00000039239 | 10116.ENSRNOP00000028744 | 152 |
| P32232\|CBS | Q62662\|FRK | 10116.ENSRNOP00000039968 | 10116.ENSRNOP00000000653 | 164 |
| P32232\|CBS | Q5HZE4\|MTNA | 10116.ENSRNOP00000039968 | 10116.ENSRNOP00000003762 | 247 |
| P32232\|CBS | Q4VBH2\|Q4VBH2 | 10116.ENSRNOP00000039968 | 10116.ENSRNOP00000008527 | 154 |
| P32232\|CBS | D3ZV30\|D3ZV30 | 10116.ENSRNOP00000039968 | 10116.ENSRNOP00000009746 | 175 |
| P32232\|CBS | D3ZBN3\|D3ZBN3 | 10116.ENSRNOP00000039968 | 10116.ENSRNOP00000012328 | 164 |
| P32232\|CBS | A0A0G2K4N5\|A0A0G2K4N5 | 10116.ENSRNOP00000039968 | 10116.ENSRNOP00000013116 | 353 |
| P32232\|CBS | O08701\|ARGI2 | 10116.ENSRNOP00000039968 | 10116.ENSRNOP00000015083 | 244 |
| P32232\|CBS | Q32KJ6\|GALNS | 10116.ENSRNOP00000039968 | 10116.ENSRNOP00000019528 | 239 |
| P32232\|CBS | D3ZUX7\|D3ZUX7 | 10116.ENSRNOP00000039968 | 10116.ENSRNOP00000020313 | 210 |
| P32232\|CBS | Q5EB90\|Q5EB90 | 10116.ENSRNOP00000039968 | 10116.ENSRNOP00000021639 | 154 |
| P32232\|CBS | D4A3P1\|D4A3P1 | 10116.ENSRNOP00000039968 | 10116.ENSRNOP00000026994 | 317 |
| P32232\|CBS | Q9ER28\|Q9ER28 | 10116.ENSRNOP00000039968 | 10116.ENSRNOP00000031063 | 181 |
| Q8CFC4\|BGAT2 | G3V757\|G3V757 | 10116.ENSRNOP00000039997 | 10116.ENSRNOP00000012176 | 903 |
| Q8CFC4\|BGAT2 | P17164\|FUCO | 10116.ENSRNOP00000039997 | 10116.ENSRNOP00000012455 | 389 |
| Q8CFC4\|BGAT2 | P97590\|LEG7 | 10116.ENSRNOP00000039997 | 10116.ENSRNOP00000027620 | 353 |
| D4A9Q5\|D4A9Q5 | B5DF79\|B5DF79 | 10116.ENSRNOP00000040541 | 10116.ENSRNOP00000017601 | 248 |
| D4A9Q5\|D4A9Q5 | Q32KJ6\|GALNS | 10116.ENSRNOP00000040541 | 10116.ENSRNOP00000019528 | 302 |
| P31503\|PO2F1 | Q2TGK3\|Q2TGK3 | 10116.ENSRNOP00000044416 | 10116.ENSRNOP00000005817 | 194 |
| P31503\|PO2F1 | P31214\|S5A2 | 10116.ENSRNOP00000044416 | 10116.ENSRNOP00000009254 | 154 |
| P31503\|PO2F1 | D3ZV30\|D3ZV30 | 10116.ENSRNOP00000044416 | 10116.ENSRNOP00000009746 | 900 |
| P31503\|PO2F1 | A3KNA0\|A3KNA0 | 10116.ENSRNOP00000044416 | 10116.ENSRNOP00000011950 | 167 |
| P31503\|PO2F1 | D3ZBN3\|D3ZBN3 | 10116.ENSRNOP00000044416 | 10116.ENSRNOP00000012328 | 176 |
| P31503\|PO2F1 | Q99068\|AMRP | 10116.ENSRNOP00000044416 | 10116.ENSRNOP00000012665 | 175 |
| D4A2K1\|D4A2K1 | D3ZV30\|D3ZV30 | 10116.ENSRNOP00000047674 | 10116.ENSRNOP00000009746 | 161 |
| D4A2K1\|D4A2K1 | Q9WVK3\|PECR | 10116.ENSRNOP00000047674 | 10116.ENSRNOP00000021512 | 251 |
| D4A2K1\|D4A2K1 | Q5EB90\|Q5EB90 | 10116.ENSRNOP00000047674 | 10116.ENSRNOP00000021639 | 171 |
| D4A2K1\|D4A2K1 | Q5U3Z3\|ISOC2 | 10116.ENSRNOP00000047674 | 10116.ENSRNOP00000022621 | 225 |
| D4A2K1\|D4A2K1 | P24050\|RS5 | 10116.ENSRNOP00000047674 | 10116.ENSRNOP00000026528 | 223 |
| D4A2K1\|D4A2K1 | F1LR52\|F1LR52 | 10116.ENSRNOP00000047674 | 10116.ENSRNOP00000033826 | 170 |
| A0A0G2K3D7\|A0A0G2K3D7 | Q62662\|FRK | 10116.ENSRNOP00000047831 | 10116.ENSRNOP00000000653 | 156 |
| A0A0G2K3D7\|A0A0G2K3D7 | D3ZBN3\|D3ZBN3 | 10116.ENSRNOP00000047831 | 10116.ENSRNOP00000012328 | 156 |
| A0A0G2K3D7\|A0A0G2K3D7 | Q4QQW4\|HDAC1 | 10116.ENSRNOP00000047831 | 10116.ENSRNOP00000012854 | 319 |
| A0A0G2K3D7\|A0A0G2K3D7 | F1LQI1\|F1LQI1 | 10116.ENSRNOP00000047831 | 10116.ENSRNOP00000020192 | 193 |
| A0A0G2K3D7\|A0A0G2K3D7 | O70597\|PX11A | 10116.ENSRNOP00000047831 | 10116.ENSRNOP00000020229 | 200 |
| A0A0G2K3D7\|A0A0G2K3D7 | D3ZCV0\|D3ZCV0 | 10116.ENSRNOP00000047831 | 10116.ENSRNOP00000024098 | 178 |
| A0A0G2K3D7\|A0A0G2K3D7 | F1LRH4\|F1LRH4 | 10116.ENSRNOP00000047831 | 10116.ENSRNOP00000037099 | 202 |
| D3ZD09\|D3ZD09 | D4A3P1\|D4A3P1 | 10116.ENSRNOP00000048723 | 10116.ENSRNOP00000026994 | 195 |
| Q9R1T5\|ACY2 | B5DEI2\|B5DEI2 | 10116.ENSRNOP00000050760 | 10116.ENSRNOP00000009520 | 900 |
| Q9R1T5\|ACY2 | P20611\|PPAL | 10116.ENSRNOP00000050760 | 10116.ENSRNOP00000018620 | 223 |
| D3ZW27\|D3ZW27 | Q62662\|FRK | 10116.ENSRNOP00000051338 | 10116.ENSRNOP00000000653 | 169 |
| D3ZW27\|D3ZW27 | D3ZBN3\|D3ZBN3 | 10116.ENSRNOP00000051338 | 10116.ENSRNOP00000012328 | 166 |
| D3ZW27\|D3ZW27 | Q4QQW4\|HDAC1 | 10116.ENSRNOP00000051338 | 10116.ENSRNOP00000012854 | 254 |
| D3ZW27\|D3ZW27 | Q9Z1L0\|PK3CB | 10116.ENSRNOP00000051338 | 10116.ENSRNOP00000022179 | 285 |
| D3ZW27\|D3ZW27 | P04961\|PCNA | 10116.ENSRNOP00000051338 | 10116.ENSRNOP00000028887 | 160 |
| D3ZW27\|D3ZW27 | Q9ER28\|Q9ER28 | 10116.ENSRNOP00000051338 | 10116.ENSRNOP00000031063 | 283 |
| D3ZW27\|D3ZW27 | D3ZCG2\|D3ZCG2 | 10116.ENSRNOP00000051338 | 10116.ENSRNOP00000031911 | 152 |
| D3ZW27\|D3ZW27 | A0A0G2K3D7\|A0A0G2K3D7 | 10116.ENSRNOP00000051338 | 10116.ENSRNOP00000047831 | 154 |
| M0R9N6\|M0R9N6 | Q62662\|FRK | 10116.ENSRNOP00000054081 | 10116.ENSRNOP00000000653 | 172 |
| M0R9N6\|M0R9N6 | D3ZV54\|D3ZV54 | 10116.ENSRNOP00000054081 | 10116.ENSRNOP00000001605 | 414 |
| M0R9N6\|M0R9N6 | Q5BKC6\|HBAP1 | 10116.ENSRNOP00000054081 | 10116.ENSRNOP00000003060 | 183 |
| M0R9N6\|M0R9N6 | Q5HZE4\|MTNA | 10116.ENSRNOP00000054081 | 10116.ENSRNOP00000003762 | 341 |
| M0R9N6\|M0R9N6 | F1LR42\|F1LR42 | 10116.ENSRNOP00000054081 | 10116.ENSRNOP00000004740 | 437 |
| M0R9N6\|M0R9N6 | D3ZCR4\|D3ZCR4 | 10116.ENSRNOP00000054081 | 10116.ENSRNOP00000005170 | 155 |
| M0R9N6\|M0R9N6 | Q62931\|GOSR1 | 10116.ENSRNOP00000054081 | 10116.ENSRNOP00000005283 | 414 |
| M0R9N6\|M0R9N6 | Q2TGK3\|Q2TGK3 | 10116.ENSRNOP00000054081 | 10116.ENSRNOP00000005817 | 218 |
| M0R9N6\|M0R9N6 | Q5XI55\|NGLY1 | 10116.ENSRNOP00000054081 | 10116.ENSRNOP00000008289 | 547 |
| M0R9N6\|M0R9N6 | Q5RKH2\|Q5RKH2 | 10116.ENSRNOP00000054081 | 10116.ENSRNOP00000008525 | 262 |
| M0R9N6\|M0R9N6 | D3ZV30\|D3ZV30 | 10116.ENSRNOP00000054081 | 10116.ENSRNOP00000009746 | 505 |
| M0R9N6\|M0R9N6 | Q9ER31\|ENTP6 | 10116.ENSRNOP00000054081 | 10116.ENSRNOP00000009946 | 150 |
| M0R9N6\|M0R9N6 | A0A0G2JV51\|A0A0G2JV51 | 10116.ENSRNOP00000054081 | 10116.ENSRNOP00000012094 | 461 |
| M0R9N6\|M0R9N6 | D3ZBN3\|D3ZBN3 | 10116.ENSRNOP00000054081 | 10116.ENSRNOP00000012328 | 155 |
| M0R9N6\|M0R9N6 | P24464\|CP4AC | 10116.ENSRNOP00000054081 | 10116.ENSRNOP00000012448 | 250 |
| M0R9N6\|M0R9N6 | Q4QQW4\|HDAC1 | 10116.ENSRNOP00000054081 | 10116.ENSRNOP00000012854 | 328 |
| M0R9N6\|M0R9N6 | A0A0G2K4N5\|A0A0G2K4N5 | 10116.ENSRNOP00000054081 | 10116.ENSRNOP00000013116 | 382 |
| M0R9N6\|M0R9N6 | P50878\|RL4 | 10116.ENSRNOP00000054081 | 10116.ENSRNOP00000013462 | 488 |
| M0R9N6\|M0R9N6 | D3ZY44\|D3ZY44 | 10116.ENSRNOP00000054081 | 10116.ENSRNOP00000013548 | 349 |
| M0R9N6\|M0R9N6 | P84039\|ENPP5 | 10116.ENSRNOP00000054081 | 10116.ENSRNOP00000013703 | 200 |
| M0R9N6\|M0R9N6 | D4AC65\|D4AC65 | 10116.ENSRNOP00000054081 | 10116.ENSRNOP00000014401 | 191 |
| M0R9N6\|M0R9N6 | Q68FU7\|COQ6 | 10116.ENSRNOP00000054081 | 10116.ENSRNOP00000014914 | 216 |
| M0R9N6\|M0R9N6 | O08701\|ARGI2 | 10116.ENSRNOP00000054081 | 10116.ENSRNOP00000015083 | 183 |
| M0R9N6\|M0R9N6 | D4A4J0\|D4A4J0 | 10116.ENSRNOP00000054081 | 10116.ENSRNOP00000016288 | 489 |
| M0R9N6\|M0R9N6 | Q9Z339\|GSTO1 | 10116.ENSRNOP00000054081 | 10116.ENSRNOP00000016851 | 163 |
| M0R9N6\|M0R9N6 | Q5XIB2\|CWC27 | 10116.ENSRNOP00000054081 | 10116.ENSRNOP00000017832 | 173 |
| M0R9N6\|M0R9N6 | D3ZUC2\|D3ZUC2 | 10116.ENSRNOP00000054081 | 10116.ENSRNOP00000018061 | 271 |
| M0R9N6\|M0R9N6 | Q9EQN5\|SMBP2 | 10116.ENSRNOP00000054081 | 10116.ENSRNOP00000018487 | 150 |
| M0R9N6\|M0R9N6 | D4A604\|D4A604 | 10116.ENSRNOP00000054081 | 10116.ENSRNOP00000019761 | 307 |
| M0R9N6\|M0R9N6 | F1LQI1\|F1LQI1 | 10116.ENSRNOP00000054081 | 10116.ENSRNOP00000020192 | 202 |
| M0R9N6\|M0R9N6 | O70597\|PX11A | 10116.ENSRNOP00000054081 | 10116.ENSRNOP00000020229 | 243 |
| M0R9N6\|M0R9N6 | D3ZUX7\|D3ZUX7 | 10116.ENSRNOP00000054081 | 10116.ENSRNOP00000020313 | 349 |
| M0R9N6\|M0R9N6 | P0C588\|CNNM4 | 10116.ENSRNOP00000054081 | 10116.ENSRNOP00000021434 | 353 |
| M0R9N6\|M0R9N6 | Q9WVK3\|PECR | 10116.ENSRNOP00000054081 | 10116.ENSRNOP00000021512 | 189 |
| M0R9N6\|M0R9N6 | D4ACK7\|D4ACK7 | 10116.ENSRNOP00000054081 | 10116.ENSRNOP00000021591 | 353 |
| M0R9N6\|M0R9N6 | Q5EB90\|Q5EB90 | 10116.ENSRNOP00000054081 | 10116.ENSRNOP00000021639 | 218 |
| M0R9N6\|M0R9N6 | D3ZCV0\|D3ZCV0 | 10116.ENSRNOP00000054081 | 10116.ENSRNOP00000024098 | 169 |
| M0R9N6\|M0R9N6 | B2RYP8\|B2RYP8 | 10116.ENSRNOP00000054081 | 10116.ENSRNOP00000024573 | 209 |
| M0R9N6\|M0R9N6 | D3ZU51\|D3ZU51 | 10116.ENSRNOP00000054081 | 10116.ENSRNOP00000025413 | 213 |
| M0R9N6\|M0R9N6 | P24050\|RS5 | 10116.ENSRNOP00000054081 | 10116.ENSRNOP00000026528 | 505 |
| M0R9N6\|M0R9N6 | D4A3P1\|D4A3P1 | 10116.ENSRNOP00000054081 | 10116.ENSRNOP00000026994 | 578 |
| M0R9N6\|M0R9N6 | D3ZHA0\|FLNC | 10116.ENSRNOP00000054081 | 10116.ENSRNOP00000027237 | 169 |
| M0R9N6\|M0R9N6 | D3ZJF7\|D3ZJF7 | 10116.ENSRNOP00000054081 | 10116.ENSRNOP00000027695 | 181 |
| M0R9N6\|M0R9N6 | Q4V7F5\|PIHD1 | 10116.ENSRNOP00000054081 | 10116.ENSRNOP00000028022 | 169 |
| M0R9N6\|M0R9N6 | P04961\|PCNA | 10116.ENSRNOP00000054081 | 10116.ENSRNOP00000028887 | 558 |
| M0R9N6\|M0R9N6 | Q9ER28\|Q9ER28 | 10116.ENSRNOP00000054081 | 10116.ENSRNOP00000031063 | 188 |
| M0R9N6\|M0R9N6 | D3ZCG2\|D3ZCG2 | 10116.ENSRNOP00000054081 | 10116.ENSRNOP00000031911 | 169 |
| M0R9N6\|M0R9N6 | F1LR52\|F1LR52 | 10116.ENSRNOP00000054081 | 10116.ENSRNOP00000033826 | 312 |
| M0R9N6\|M0R9N6 | Q562C7\|PUM3 | 10116.ENSRNOP00000054081 | 10116.ENSRNOP00000034834 | 203 |
| M0R9N6\|M0R9N6 | F1LRQ6\|F1LRQ6 | 10116.ENSRNOP00000054081 | 10116.ENSRNOP00000035675 | 206 |
| M0R9N6\|M0R9N6 | D3ZGW2\|D3ZGW2 | 10116.ENSRNOP00000054081 | 10116.ENSRNOP00000036180 | 368 |
| M0R9N6\|M0R9N6 | P32232\|CBS | 10116.ENSRNOP00000054081 | 10116.ENSRNOP00000039968 | 482 |
| M0R9N6\|M0R9N6 | A0A0G2K3D7\|A0A0G2K3D7 | 10116.ENSRNOP00000054081 | 10116.ENSRNOP00000047831 | 216 |
| M0R9N6\|M0R9N6 | D3ZD09\|D3ZD09 | 10116.ENSRNOP00000054081 | 10116.ENSRNOP00000048723 | 299 |
| M0R9N6\|M0R9N6 | D3ZW27\|D3ZW27 | 10116.ENSRNOP00000054081 | 10116.ENSRNOP00000051338 | 177 |
| O08836\|IGBP1 | D3ZCR4\|D3ZCR4 | 10116.ENSRNOP00000054708 | 10116.ENSRNOP00000005170 | 156 |
| O08836\|IGBP1 | P30904\|MIF | 10116.ENSRNOP00000054708 | 10116.ENSRNOP00000008608 | 183 |
| O08836\|IGBP1 | Q63525\|NUDC | 10116.ENSRNOP00000054708 | 10116.ENSRNOP00000009933 | 206 |
| O08836\|IGBP1 | Q9Z1L0\|PK3CB | 10116.ENSRNOP00000054708 | 10116.ENSRNOP00000022179 | 355 |
| O08836\|IGBP1 | D4A3P1\|D4A3P1 | 10116.ENSRNOP00000054708 | 10116.ENSRNOP00000026994 | 205 |
| O08836\|IGBP1 | M0R9N6\|M0R9N6 | 10116.ENSRNOP00000054708 | 10116.ENSRNOP00000054081 | 309 |
| D3ZEL0\|D3ZEL0 | G3V6P6\|G3V6P6 | 10116.ENSRNOP00000055154 | 10116.ENSRNOP00000007367 | 249 |
| D3ZEL0\|D3ZEL0 | Q66HG4\|GALM | 10116.ENSRNOP00000055154 | 10116.ENSRNOP00000009221 | 351 |
| D3ZEL0\|D3ZEL0 | A3KNA0\|A3KNA0 | 10116.ENSRNOP00000055154 | 10116.ENSRNOP00000011950 | 441 |
| D3ZEL0\|D3ZEL0 | P60825\|CIRBP | 10116.ENSRNOP00000055154 | 10116.ENSRNOP00000021648 | 249 |
| D3ZG54\|D3ZG54 | Q62662\|FRK | 10116.ENSRNOP00000055573 | 10116.ENSRNOP00000000653 | 168 |
| D3ZG54\|D3ZG54 | Q66HG4\|GALM | 10116.ENSRNOP00000055573 | 10116.ENSRNOP00000009221 | 395 |
| D3ZG54\|D3ZG54 | Q64380\|SARDH | 10116.ENSRNOP00000055573 | 10116.ENSRNOP00000009555 | 437 |
| D3ZG54\|D3ZG54 | D3ZBN3\|D3ZBN3 | 10116.ENSRNOP00000055573 | 10116.ENSRNOP00000012328 | 205 |
| D3ZG54\|D3ZG54 | P24464\|CP4AC | 10116.ENSRNOP00000055573 | 10116.ENSRNOP00000012448 | 181 |
| D3ZG54\|D3ZG54 | A0A0G2K4N5\|A0A0G2K4N5 | 10116.ENSRNOP00000055573 | 10116.ENSRNOP00000013116 | 155 |
| D3ZG54\|D3ZG54 | D3ZY44\|D3ZY44 | 10116.ENSRNOP00000055573 | 10116.ENSRNOP00000013548 | 177 |
| D3ZG54\|D3ZG54 | O08701\|ARGI2 | 10116.ENSRNOP00000055573 | 10116.ENSRNOP00000015083 | 161 |
| D3ZG54\|D3ZG54 | B5DF79\|B5DF79 | 10116.ENSRNOP00000055573 | 10116.ENSRNOP00000017601 | 169 |
| D3ZG54\|D3ZG54 | D3ZUX7\|D3ZUX7 | 10116.ENSRNOP00000055573 | 10116.ENSRNOP00000020313 | 526 |
| D3ZG54\|D3ZG54 | P0C588\|CNNM4 | 10116.ENSRNOP00000055573 | 10116.ENSRNOP00000021434 | 167 |
| D3ZG54\|D3ZG54 | Q9WVK3\|PECR | 10116.ENSRNOP00000055573 | 10116.ENSRNOP00000021512 | 247 |
| D3ZG54\|D3ZG54 | D4ACK7\|D4ACK7 | 10116.ENSRNOP00000055573 | 10116.ENSRNOP00000021591 | 167 |
| D3ZG54\|D3ZG54 | Q9Z1L0\|PK3CB | 10116.ENSRNOP00000055573 | 10116.ENSRNOP00000022179 | 159 |
| D3ZG54\|D3ZG54 | Q8CG45\|ARK72 | 10116.ENSRNOP00000055573 | 10116.ENSRNOP00000024063 | 173 |
| D3ZG54\|D3ZG54 | D3ZCV0\|D3ZCV0 | 10116.ENSRNOP00000055573 | 10116.ENSRNOP00000024098 | 185 |
| D3ZG54\|D3ZG54 | D4A3P1\|D4A3P1 | 10116.ENSRNOP00000055573 | 10116.ENSRNOP00000026994 | 510 |
| D3ZG54\|D3ZG54 | P04961\|PCNA | 10116.ENSRNOP00000055573 | 10116.ENSRNOP00000028887 | 155 |
| D3ZG54\|D3ZG54 | F1LR52\|F1LR52 | 10116.ENSRNOP00000055573 | 10116.ENSRNOP00000033826 | 254 |
| D3ZG54\|D3ZG54 | D4AA35\|D4AA35 | 10116.ENSRNOP00000055573 | 10116.ENSRNOP00000038215 | 157 |
| D3ZG54\|D3ZG54 | P32232\|CBS | 10116.ENSRNOP00000055573 | 10116.ENSRNOP00000039968 | 163 |
| D3ZG54\|D3ZG54 | D4A2K1\|D4A2K1 | 10116.ENSRNOP00000055573 | 10116.ENSRNOP00000047674 | 255 |
| D3ZG54\|D3ZG54 | M0R9N6\|M0R9N6 | 10116.ENSRNOP00000055573 | 10116.ENSRNOP00000054081 | 510 |
| A0A1W2Q6H4\|A0A1W2Q6H4 | A0A0G2K4N5\|A0A0G2K4N5 | 10116.ENSRNOP00000057034 | 10116.ENSRNOP00000013116 | 288 |
| A0A1W2Q6H4\|A0A1W2Q6H4 | Q68FU7\|COQ6 | 10116.ENSRNOP00000057034 | 10116.ENSRNOP00000014914 | 317 |
| A0A1W2Q6H4\|A0A1W2Q6H4 | D3ZUX7\|D3ZUX7 | 10116.ENSRNOP00000057034 | 10116.ENSRNOP00000020313 | 460 |
| A0A1W2Q6H4\|A0A1W2Q6H4 | B2RYP8\|B2RYP8 | 10116.ENSRNOP00000057034 | 10116.ENSRNOP00000024573 | 153 |
| A0A1W2Q6H4\|A0A1W2Q6H4 | D4A3P1\|D4A3P1 | 10116.ENSRNOP00000057034 | 10116.ENSRNOP00000026994 | 168 |
| A0A1W2Q6H4\|A0A1W2Q6H4 | M0R9N6\|M0R9N6 | 10116.ENSRNOP00000057034 | 10116.ENSRNOP00000054081 | 250 |
| A0A1W2Q6H4\|A0A1W2Q6H4 | D3ZG54\|D3ZG54 | 10116.ENSRNOP00000057034 | 10116.ENSRNOP00000055573 | 155 |
| D3ZNK1\|D3ZNK1 | Q5HZE4\|MTNA | 10116.ENSRNOP00000058474 | 10116.ENSRNOP00000003762 | 697 |
| D3ZNK1\|D3ZNK1 | Q9JKW1\|TIM22 | 10116.ENSRNOP00000058474 | 10116.ENSRNOP00000010779 | 353 |
| D3ZNK1\|D3ZNK1 | P62078\|TIM8B | 10116.ENSRNOP00000058474 | 10116.ENSRNOP00000013188 | 402 |
| D3ZNK1\|D3ZNK1 | D4A3P1\|D4A3P1 | 10116.ENSRNOP00000058474 | 10116.ENSRNOP00000026994 | 210 |
| D3ZNK1\|D3ZNK1 | M0R9N6\|M0R9N6 | 10116.ENSRNOP00000058474 | 10116.ENSRNOP00000054081 | 198 |
| Q05820\|LYSC2 | Q6IFV1\|K1C14 | 10116.ENSRNOP00000059657 | 10116.ENSRNOP00000005285 | 207 |
| Q05820\|LYSC2 | Q5RKH2\|Q5RKH2 | 10116.ENSRNOP00000059657 | 10116.ENSRNOP00000008525 | 183 |
| Q05820\|LYSC2 | G3V757\|G3V757 | 10116.ENSRNOP00000059657 | 10116.ENSRNOP00000012176 | 186 |
| A0A0G2JVH5\|A0A0G2JVH5 | Q62662\|FRK | 10116.ENSRNOP00000060341 | 10116.ENSRNOP00000000653 | 160 |
| A0A0G2JVH5\|A0A0G2JVH5 | A0A0G2K402\|A0A0G2K402 | 10116.ENSRNOP00000060341 | 10116.ENSRNOP00000007103 | 156 |
| A0A0G2JVH5\|A0A0G2JVH5 | Q4VBH2\|Q4VBH2 | 10116.ENSRNOP00000060341 | 10116.ENSRNOP00000008527 | 157 |
| A0A0G2JVH5\|A0A0G2JVH5 | D3ZV30\|D3ZV30 | 10116.ENSRNOP00000060341 | 10116.ENSRNOP00000009746 | 455 |
| A0A0G2JVH5\|A0A0G2JVH5 | D3ZBN3\|D3ZBN3 | 10116.ENSRNOP00000060341 | 10116.ENSRNOP00000012328 | 160 |
| A0A0G2JVH5\|A0A0G2JVH5 | Q4QQW4\|HDAC1 | 10116.ENSRNOP00000060341 | 10116.ENSRNOP00000012854 | 361 |
| A0A0G2JVH5\|A0A0G2JVH5 | D4A4J0\|D4A4J0 | 10116.ENSRNOP00000060341 | 10116.ENSRNOP00000016288 | 483 |
| A0A0G2JVH5\|A0A0G2JVH5 | Q5EB90\|Q5EB90 | 10116.ENSRNOP00000060341 | 10116.ENSRNOP00000021639 | 322 |
| A0A0G2JVH5\|A0A0G2JVH5 | Q9Z1L0\|PK3CB | 10116.ENSRNOP00000060341 | 10116.ENSRNOP00000022179 | 177 |
| A0A0G2JVH5\|A0A0G2JVH5 | D4A3P1\|D4A3P1 | 10116.ENSRNOP00000060341 | 10116.ENSRNOP00000026994 | 197 |
| A0A0G2JVH5\|A0A0G2JVH5 | P04961\|PCNA | 10116.ENSRNOP00000060341 | 10116.ENSRNOP00000028887 | 337 |
| A0A0G2JVH5\|A0A0G2JVH5 | M0R9N6\|M0R9N6 | 10116.ENSRNOP00000060341 | 10116.ENSRNOP00000054081 | 412 |
| Q66H12\|NAGAB | A0A0G2JY11\|A0A0G2JY11 | 10116.ENSRNOP00000060590 | 10116.ENSRNOP00000007328 | 353 |
| Q66H12\|NAGAB | P17164\|FUCO | 10116.ENSRNOP00000060590 | 10116.ENSRNOP00000012455 | 241 |
| Q66H12\|NAGAB | Q6AYS4\|FUCO2 | 10116.ENSRNOP00000060590 | 10116.ENSRNOP00000020946 | 199 |
| Q66H12\|NAGAB | P97590\|LEG7 | 10116.ENSRNOP00000060590 | 10116.ENSRNOP00000027620 | 150 |
| P70490\|MFGM | Q05820\|LYSC2 | 10116.ENSRNOP00000060643 | 10116.ENSRNOP00000059657 | 900 |
| P0C2C4\|RM10 | D4A604\|D4A604 | 10116.ENSRNOP00000061129 | 10116.ENSRNOP00000019761 | 177 |
| D4A626\|D4A626 | Q6AYC4\|CAPG | 10116.ENSRNOP00000061235 | 10116.ENSRNOP00000018562 | 153 |
| D4A626\|D4A626 | P15205\|MAP1B | 10116.ENSRNOP00000061235 | 10116.ENSRNOP00000023460 | 199 |
| D4A626\|D4A626 | D4A3P1\|D4A3P1 | 10116.ENSRNOP00000061235 | 10116.ENSRNOP00000026994 | 169 |
| D4A626\|D4A626 | M0R9N6\|M0R9N6 | 10116.ENSRNOP00000061235 | 10116.ENSRNOP00000054081 | 169 |
| A0A0G2K2P4\|A0A0G2K2P4 | P24464\|CP4AC | 10116.ENSRNOP00000061482 | 10116.ENSRNOP00000012448 | 313 |
| A0A0G2K2P4\|A0A0G2K2P4 | Q68FU7\|COQ6 | 10116.ENSRNOP00000061482 | 10116.ENSRNOP00000014914 | 317 |
| A0A0G2K2P4\|A0A0G2K2P4 | D4A4J0\|D4A4J0 | 10116.ENSRNOP00000061482 | 10116.ENSRNOP00000016288 | 557 |
| A0A0G2K2P4\|A0A0G2K2P4 | D3ZUX7\|D3ZUX7 | 10116.ENSRNOP00000061482 | 10116.ENSRNOP00000020313 | 460 |
| A0A0G2K2P4\|A0A0G2K2P4 | D4A3P1\|D4A3P1 | 10116.ENSRNOP00000061482 | 10116.ENSRNOP00000026994 | 168 |
| A0A0G2K2P4\|A0A0G2K2P4 | M0R9N6\|M0R9N6 | 10116.ENSRNOP00000061482 | 10116.ENSRNOP00000054081 | 168 |
| A0A0G2K2P4\|A0A0G2K2P4 | D3ZG54\|D3ZG54 | 10116.ENSRNOP00000061482 | 10116.ENSRNOP00000055573 | 229 |
| A0A0G2K2P4\|A0A0G2K2P4 | A0A1W2Q6H4\|A0A1W2Q6H4 | 10116.ENSRNOP00000061482 | 10116.ENSRNOP00000057034 | 236 |
| D4A997\|D4A997 | Q6MGB6\|RING1 | 10116.ENSRNOP00000061583 | 10116.ENSRNOP00000000543 | 479 |
| D4A997\|D4A997 | G3V6P6\|G3V6P6 | 10116.ENSRNOP00000061583 | 10116.ENSRNOP00000007367 | 215 |
| D4A997\|D4A997 | P60825\|CIRBP | 10116.ENSRNOP00000061583 | 10116.ENSRNOP00000021648 | 215 |
| D4A997\|D4A997 | A9CMA7\|A9CMA7 | 10116.ENSRNOP00000061583 | 10116.ENSRNOP00000024602 | 679 |
| D4A997\|D4A997 | F1LQC8\|F1LQC8 | 10116.ENSRNOP00000061583 | 10116.ENSRNOP00000025026 | 307 |
| D4A997\|D4A997 | M0R9N6\|M0R9N6 | 10116.ENSRNOP00000061583 | 10116.ENSRNOP00000054081 | 150 |
| D4A2N2\|D4A2N2 | Q62662\|FRK | 10116.ENSRNOP00000061755 | 10116.ENSRNOP00000000653 | 266 |
| D4A2N2\|D4A2N2 | Q64380\|SARDH | 10116.ENSRNOP00000061755 | 10116.ENSRNOP00000009555 | 181 |
| D4A2N2\|D4A2N2 | Q9Z1L0\|PK3CB | 10116.ENSRNOP00000061755 | 10116.ENSRNOP00000022179 | 223 |
| D4A2N2\|D4A2N2 | D3ZCV0\|D3ZCV0 | 10116.ENSRNOP00000061755 | 10116.ENSRNOP00000024098 | 218 |
| D4A2N2\|D4A2N2 | D4A3P1\|D4A3P1 | 10116.ENSRNOP00000061755 | 10116.ENSRNOP00000026994 | 193 |
| D4A2N2\|D4A2N2 | D3ZHA0\|FLNC | 10116.ENSRNOP00000061755 | 10116.ENSRNOP00000027237 | 287 |
| D4A2N2\|D4A2N2 | D3ZGW2\|D3ZGW2 | 10116.ENSRNOP00000061755 | 10116.ENSRNOP00000036180 | 388 |
| D4A2N2\|D4A2N2 | Q8CJ11\|AGRG2 | 10116.ENSRNOP00000061755 | 10116.ENSRNOP00000039239 | 159 |
| D4A2N2\|D4A2N2 | M0R9N6\|M0R9N6 | 10116.ENSRNOP00000061755 | 10116.ENSRNOP00000054081 | 361 |
| D4A2N2\|D4A2N2 | D4A626\|D4A626 | 10116.ENSRNOP00000061755 | 10116.ENSRNOP00000061235 | 203 |
| B5DEL5\|B5DEL5 | D3ZCR4\|D3ZCR4 | 10116.ENSRNOP00000061990 | 10116.ENSRNOP00000005170 | 210 |
| B5DEL5\|B5DEL5 | Q62931\|GOSR1 | 10116.ENSRNOP00000061990 | 10116.ENSRNOP00000005283 | 237 |
| B5DEL5\|B5DEL5 | Q4VBH2\|Q4VBH2 | 10116.ENSRNOP00000061990 | 10116.ENSRNOP00000008527 | 232 |
| B5DEL5\|B5DEL5 | D3ZW27\|D3ZW27 | 10116.ENSRNOP00000061990 | 10116.ENSRNOP00000051338 | 193 |
| D3ZEA0\|D3ZEA0 | P0C588\|CNNM4 | 10116.ENSRNOP00000062773 | 10116.ENSRNOP00000021434 | 351 |
| D3ZEA0\|D3ZEA0 | D4ACK7\|D4ACK7 | 10116.ENSRNOP00000062773 | 10116.ENSRNOP00000021591 | 384 |
| P11466\|OCTC | F1LR42\|F1LR42 | 10116.ENSRNOP00000063856 | 10116.ENSRNOP00000004740 | 199 |
| P11466\|OCTC | Q9JJ46\|EBP | 10116.ENSRNOP00000063856 | 10116.ENSRNOP00000007015 | 204 |
| P11466\|OCTC | P24464\|CP4AC | 10116.ENSRNOP00000063856 | 10116.ENSRNOP00000012448 | 158 |
| P11466\|OCTC | A0A0G2JU12\|A0A0G2JU12 | 10116.ENSRNOP00000063856 | 10116.ENSRNOP00000017785 | 190 |
| P11466\|OCTC | D4A604\|D4A604 | 10116.ENSRNOP00000063856 | 10116.ENSRNOP00000019761 | 175 |
| P11466\|OCTC | O70597\|PX11A | 10116.ENSRNOP00000063856 | 10116.ENSRNOP00000020229 | 252 |
| P11466\|OCTC | Q9WVK3\|PECR | 10116.ENSRNOP00000063856 | 10116.ENSRNOP00000021512 | 194 |
| P11466\|OCTC | Q9Z122\|FADS2 | 10116.ENSRNOP00000063856 | 10116.ENSRNOP00000027756 | 185 |
| P11466\|OCTC | Q9R1T5\|ACY2 | 10116.ENSRNOP00000063856 | 10116.ENSRNOP00000050760 | 240 |
| M0R3V4\|M0R3V4 | P30904\|MIF | 10116.ENSRNOP00000064027 | 10116.ENSRNOP00000008608 | 167 |
| M0R3V4\|M0R3V4 | P24050\|RS5 | 10116.ENSRNOP00000064027 | 10116.ENSRNOP00000026528 | 159 |
| Q9R1B1\|T10B | Q63525\|NUDC | 10116.ENSRNOP00000064088 | 10116.ENSRNOP00000009933 | 195 |
| Q9R1B1\|T10B | Q9JKW1\|TIM22 | 10116.ENSRNOP00000064088 | 10116.ENSRNOP00000010779 | 772 |
| Q9R1B1\|T10B | P62078\|TIM8B | 10116.ENSRNOP00000064088 | 10116.ENSRNOP00000013188 | 933 |
| Q9R1B1\|T10B | P24050\|RS5 | 10116.ENSRNOP00000064088 | 10116.ENSRNOP00000026528 | 199 |
| Q9R1B1\|T10B | D3ZD09\|D3ZD09 | 10116.ENSRNOP00000064088 | 10116.ENSRNOP00000048723 | 239 |
| Q9R1B1\|T10B | D3ZNK1\|D3ZNK1 | 10116.ENSRNOP00000064088 | 10116.ENSRNOP00000058474 | 223 |
| Q9ET32\|BGAT1 | G3V757\|G3V757 | 10116.ENSRNOP00000065701 | 10116.ENSRNOP00000012176 | 903 |
| Q9ET32\|BGAT1 | P17164\|FUCO | 10116.ENSRNOP00000065701 | 10116.ENSRNOP00000012455 | 284 |
| Q9ET32\|BGAT1 | P97590\|LEG7 | 10116.ENSRNOP00000065701 | 10116.ENSRNOP00000027620 | 353 |
| Q9ET32\|BGAT1 | Q8CFC4\|BGAT2 | 10116.ENSRNOP00000065701 | 10116.ENSRNOP00000039997 | 901 |
| Q9R1T3\|CATZ | G3V757\|G3V757 | 10116.ENSRNOP00000066523 | 10116.ENSRNOP00000012176 | 154 |
| Q9R1T3\|CATZ | B5DF79\|B5DF79 | 10116.ENSRNOP00000066523 | 10116.ENSRNOP00000017601 | 341 |
| Q9R1T3\|CATZ | Q6AYC4\|CAPG | 10116.ENSRNOP00000066523 | 10116.ENSRNOP00000018562 | 254 |
| Q9R1T3\|CATZ | P20611\|PPAL | 10116.ENSRNOP00000066523 | 10116.ENSRNOP00000018620 | 246 |
| Q9R1T3\|CATZ | Q9WVK3\|PECR | 10116.ENSRNOP00000066523 | 10116.ENSRNOP00000021512 | 196 |
| Q9R1T3\|CATZ | Q9ER28\|Q9ER28 | 10116.ENSRNOP00000066523 | 10116.ENSRNOP00000031063 | 177 |
| Q9R1T3\|CATZ | Q9R1T5\|ACY2 | 10116.ENSRNOP00000066523 | 10116.ENSRNOP00000050760 | 150 |
| Q78EG7\|TP4A1 | Q6MGB6\|RING1 | 10116.ENSRNOP00000067713 | 10116.ENSRNOP00000000543 | 230 |
| Q78EG7\|TP4A1 | Q62662\|FRK | 10116.ENSRNOP00000067713 | 10116.ENSRNOP00000000653 | 402 |
| Q78EG7\|TP4A1 | D3ZUC2\|D3ZUC2 | 10116.ENSRNOP00000067713 | 10116.ENSRNOP00000018061 | 212 |
| Q78EG7\|TP4A1 | P60825\|CIRBP | 10116.ENSRNOP00000067713 | 10116.ENSRNOP00000021648 | 200 |
| Q78EG7\|TP4A1 | Q63619\|COQ7 | 10116.ENSRNOP00000067713 | 10116.ENSRNOP00000022988 | 190 |
| Q78EG7\|TP4A1 | P07150\|ANXA1 | 10116.ENSRNOP00000067713 | 10116.ENSRNOP00000023664 | 161 |
| Q78EG7\|TP4A1 | D4A2N2\|D4A2N2 | 10116.ENSRNOP00000067713 | 10116.ENSRNOP00000061755 | 153 |
